# A Comprehensive Analysis of the Association Between *SNCA* Polymorphisms and the Risk of Parkinson's Disease

**DOI:** 10.3389/fnmol.2018.00391

**Published:** 2018-10-25

**Authors:** Yuan Zhang, Li Shu, Qiying Sun, Hongxu Pan, Jifeng Guo, Beisha Tang

**Affiliations:** ^1^Department of Neurology, Xiangya Hospital, Central South University, Changsha, China; ^2^Department of Geriatrics, Xiangya Hospital, Central South University, Changsha, China; ^3^National Clinical Research Center for Geriatric Disorders, Changsha, China; ^4^Key Laboratory of Hunan Province in Neurodegenerative Disorders, Central South University, Changsha, China; ^5^Parkinson's Disease Center of Beijing Institute for Brain Disorders, Beijing, China; ^6^Collaborative Innovation Center for Brain Science, Shanghai, China; ^7^Collaborative Innovation Center for Genetics and Development, Shanghai, China; ^8^Center for Medical Genetics, School of Life Sciences, Central South University, Changsha, China

**Keywords:** Parkinson's disease, *SNCA*, Asian, European, meta-analysis

## Abstract

**Background:** Various studies have reported associations between synuclein alpha (*SNCA*) polymorphisms and Parkinson's disease (PD) risk. However, the results are inconsistent. We conducted a comprehensive meta-analysis of the associations between *SNCA* single-nucleotide polymorphisms (SNPs) and PD risk in overall populations and subpopulations by ethnicity.

**Methods:** Standard meta-analysis was conducted according to our protocol with a cutoff point of *p* < 0.05. To find the most relevant *SNCA* SNPs, we used a cutoff point of *p* < 1 × 10^−5^ in an analysis based on the allele model. In the subgroup analysis by ethnicity, we divided the overall populations into five ethnic groups. We conducted further analysis on the most relevant SNPs using dominant and recessive models to identify the contributions of heterozygotes and homozygotes regarding each SNP.

**Results:** In our comprehensive meta-analysis, 24,075 cases and 22,877 controls from 36 articles were included. We included 16 variants in the meta-analysis and found 12 statistically significant variants with *p* < 0.05. After narrowing down the variants using the *p* < 1 × 10^−5^ cutoff, in overall populations, seven SNPs increased the risk of PD (rs2736990, rs356220, rs356165, rs181489, rs356219, rs11931074, and rs2737029, with odds ratios [ORs] of 1.22–1.38) and one SNP decreased the risk (rs356186, with an OR of 0.77). In the East Asian group, rs2736990 and rs11931074 increased the risk (with ORs of 1.22–1.34). In the European group, five SNPs increased the risk (rs356219, rs181489, rs2737029, rs356165, and rs11931074, with ORs of 1.26–1.37) while one SNP decreased the risk (rs356186, with an OR of 0.77). The heterozygotes and homozygotes contributed differently depending on the variant.

**Conclusions:** In summary, we found eight *SNCA* SNPs associated with PD risk, which had obvious differences between ethnicities. Seven SNPs increased the risk of PD and one SNP decreased the risk in the overall populations. In the East Asian group, rs2736990 and rs11931074 increased the risk. In the European group, rs356219, rs181489, rs2737029, rs356165, and rs11931074 increased the risk while rs356186 decreased the risk. Variants with the highest ORs and allele frequencies in our analysis should be given priority when carrying out genetic screening.

## Introduction

Parkinson's disease (PD) is a common neurodegenerative disease. Bradykinesia, resting tremor, rigidity and postural instability are the prominent motor features of PD, and they are caused by dopamine-containing neuron loss in the substantia nigra. Another pathological characteristic is Lewy body accumulation in surviving neurons (Coppede, 2012; Rochet et al., 2012), the major component of which is synuclein alpha (SNCA) (Chartier-Harlin et al., 2004). The mechanism of PD remains elusive. Genetics, environmental factors, aging and their interactions are considered to be major factors that influence how the disease develops. Regarding the genetic factors, *SNCA, LRRK2*, and *DJ-1* have been found to be associated with PD risk (Tang et al., 2006; Guo et al., 2010; Wang et al., 2010; Lv et al., 2012).

*SNCA* was first found to be associated with familial autosomal dominant PD (ADPD) in 1997 (Coppede, 2012). In addition to pathogenic *SNCA* point mutations or multiplications (Chartier-Harlin et al., 2004), single-nucleotide polymorphisms (SNPs) can affect disease risks by affecting gene expression (Deng and Yuan, 2014). Since the Rep1 263-bp allele was shown to be associated with PD risk, SNPs such as rs356219, rs356165, rs11931074, rs7684318 etc., have been shown to be associated with PD risks in Caucasian or Asian populations (Mizuta et al., 2006; Ross et al., 2007; Myhre et al., 2008). Heterogeneities were observed among risk variants of *SNCA* from various populations. For example, rs356221, rs3822086, and rs11931074 were risk variants in East Asian populations while rs356186, rs2736990 were risk variants in European populations (Chung et al., 2011; Trotta et al., 2012; Wu-Chou et al., 2013; Chen et al., 2015). Genome-wide association studies (GWAS) had discovered multiple risk loci in PD and *SNCA* was among the top hits associated with PD risk (Davis et al., 2016). Several variants such as rs8180209, rs3756063, rs356165 had been found in GWAS of PD cohorts (Wei et al., 2016; Foo et al., 2017). In a large-scale meta-analysis of GWAS data, polymorphisms rs7681154 in *SNCA* also been proved to be related to risk of PD (Nalls et al., 2014). However, the results of these original studies in specific geographical areas were inconsistent and they were based on limited sample sizes. In 2015, a meta-analysis demonstrated that *SNCA* SNPs such as rs356186, rs356219, rs894278, rs2583988, and rs2619363 were common risk variants (Chartier-Harlin et al., 2004). However, the meta-analysis set a cutoff point of *p* < 0.05, which was too high to evaluate many variants at the same time without adjusting *p*-values by the numbers of variants. Moreover, the ethnic differences between different areas were not clearly stated. With the publication of more articles, the role of these variants plus other candidate variants in the risk of PD needed to be clarified, so we conducted a comprehensive analysis on the association between *SNCA* SNPs and the risk of PD in the overall populations and subpopulations by ethnicity.

## Materials and methods

### Inclusion criteria

We performed our meta-analysis based on PICOS (participants, interventions, controls, outcomes and studies) rules. The following inclusion criteria were used:

Participants: all PD patients were diagnosed based on the UK Parkinson's Disease Society Brain Bank (UKBB) Clinical Diagnostic Criteria or other accepted criteria (Hughes et al., 1992).

Interventions: genetic analysis was carried out by PCR-based methods or other accepted methods.

Controls: all controls were reported controls that had no obvious neurological diseases.

Outcomes: all participants were clearly reported the status of responsive genotypes, including homozygotes and heterozygotes.

Studies: all studies were case-control or cohort studies.

### Literature search

Three English language databases were used: Medline in PubMed, Embase in Ovid and Cochrane databases. In addition, two Chinese databases were used: CNKI and Wanfang databases. The date of the search was Dec 1, 2017. The keywords were listed separately as follows: “*SNCA*,” “*PARK1*,” “α-synuclein,” “PD,” “Parkinso^*^/$,” “polymorphism,” “SNP,” “variant,” “genetic.”

### Data extraction

The data extraction was carried out by two researchers. A third researcher was asked to resolve any disputes. The first author, publication year, ethnicity, country, gene, variants, number of cases, and controls and responsive genotypes' carriers among the cases and controls were retrieved, and they are shown in Table 1 and Supplementary Table 1. The ethnic groups comprised five groups: European, East Asian, Latino, West Asian and Mixed (i.e., at least two ethnicities) (Risch et al., 2002). Newcastle-Ottawa Scale (NOS) scores (Stang, 2010) were used to evaluate the quality of all the studies.

**Table 1:** Thirty six original articles included in the meta-analysis.

**Year and first author**	**Ethnicity**	**Country**	**Total NO. of PD/controls**	**NOS**
2006Mizuta I (Mizuta et al., 2006)	East Asian	Japan	882/938	7
2008Mizuta I (Mizuta et al., 2008)	East Asian	Japan	1403/1941	8
2010Chang XL (Chang et al., 2011)	East Asian	China	636/510	9
2010Hu FY (Hu et al., 2010)	East Asian	China	330/300	7
2010Yu L (Yu et al., 2010)	East Asian	China	332/300	8
2012Gan R(Gan et al., 2012)	East Asian	China	189/189	6
2012Hu Y (Hu et al., 2012)	East Asian	China	110/136	7
2012Li NN (Li et al., 2013)	East Asian	China	685/569	7
2012Miyake Y (Miyake et al., 2012)	East Asian	Japan	229/357	8
2012Pan F (Pan et al., 2012)	East Asian	China	403/315	7
2013Liu B (Liu B. et al., 2013)	East Asian	China	116/95	6
2013Liu J (Liu J. et al., 2013)	East Asian	China	989/748	7
2013Pan F (Pan et al., 2013)	East Asian	China	515/450	8
2013Wu-Chou YH (Wu-Chou et al., 2013)	East Asian	China	633/473	8
2014Guo XY (Guo et al., 2014)	East Asian	China	1011/721	7
2015Chen YP (Chen et al., 2015)	East Asian	China	1276/846	7
2015Guo JF (Guo et al., 2015)	East Asian	China	1061/1066	8
2015Wu GP (Wu et al., 2015)	East Asian	China	120/100	6
2016Fang J (Fang et al., 2016)	East Asian	China	583/553	8
2017Chen WJ (Chen et al., 2017)	East Asian	China	210/251	7
2007Goris A (Goris et al., 2007)	European	UK	659/2176	7
2007Parsian AJ (Parsian et al., 2007)	European	America	521/215	8
2007Ross OA (Ross et al., 2007)	European	Ireland	186/186	9
2007Winkler S (Winkler et al., 2007)	European	Germany	397/270	7
2008Myhre R (Myhre et al., 2008)	European	Norway	236/236	8
2008Westerlund M (Westerlund et al., 2008)	European	Sweden	290/313	9
2009Chung SJ (Chung et al., 2011)	European	America	1103/1103	7
2009Pankratz N (Pankratz et al., 2009)	European	America	445/335	7
2010Mata IF (Mata et al., 2010)	European	America	685/673	8
2011Elbaz A (Elbaz et al., 2011)	European	Multi-country	5302/4161	7
2012Cardo LF (Cardo et al., 2012)	European	Spain	1135/772	6
2012Trotta L (Trotta et al., 2012)	European	Italy	904/891	6
2013Emelyanov A (Emelyanov et al., 2013)	European	Russia	224/308	7
2017Campêlo CL (Campelo et al., 2017)	Latino	Brazil	104/98	7
2014Lee PC (Lee et al., 2015)	Mixed	America	533/700	8
2016Shahmohammadibeni N (Shahmohammadibeni et al., 2016)	West Asian	Iran	520/520	7

*NO., number; NOS, Newcastle-Ottawa Scale scores*.

### Statistical analysis

Meta-analyses were carried out using Revman 5.3 software. Pooled analyses of variants (involving at least four original articles) were conducted in the overall populations or subpopulations by ethnicity. The pooled analyses were conducted using the allele model. *P* < 0.05 was considered to represent statistically significant differences in the allele model. Dominant and recessive models were applied to analyze the contributions of heterozygotes and homozygotes concerning each variant that had a significant difference in the allele model (*p* < 1 × 10^−5^). The significance of each of the dominant and recessive models was also reflected by *p* < 1 × 10^−5^. Allele frequencies (AFs) were calculated in the overall populations and subpopulations by ethnicity. Pooled odds ratios (ORs) and 95% CIs were calculated to assess the significance of the results. *Q* and *I*^2^ statistics were used to demonstrate the heterogeneity of the analysis. If *Q* statistic *p* > 0.1 or *I*^2^ ≤ 50%, a fixed-effects model were used for the analysis. Otherwise, a random-effects model was applied. Sensitivity analysis was carried out by deleting each original article one at a time. Publication bias was assessed based on the symmetry of the funnel plot.

## Results

### Characteristics of the studies

**Figure 1:**
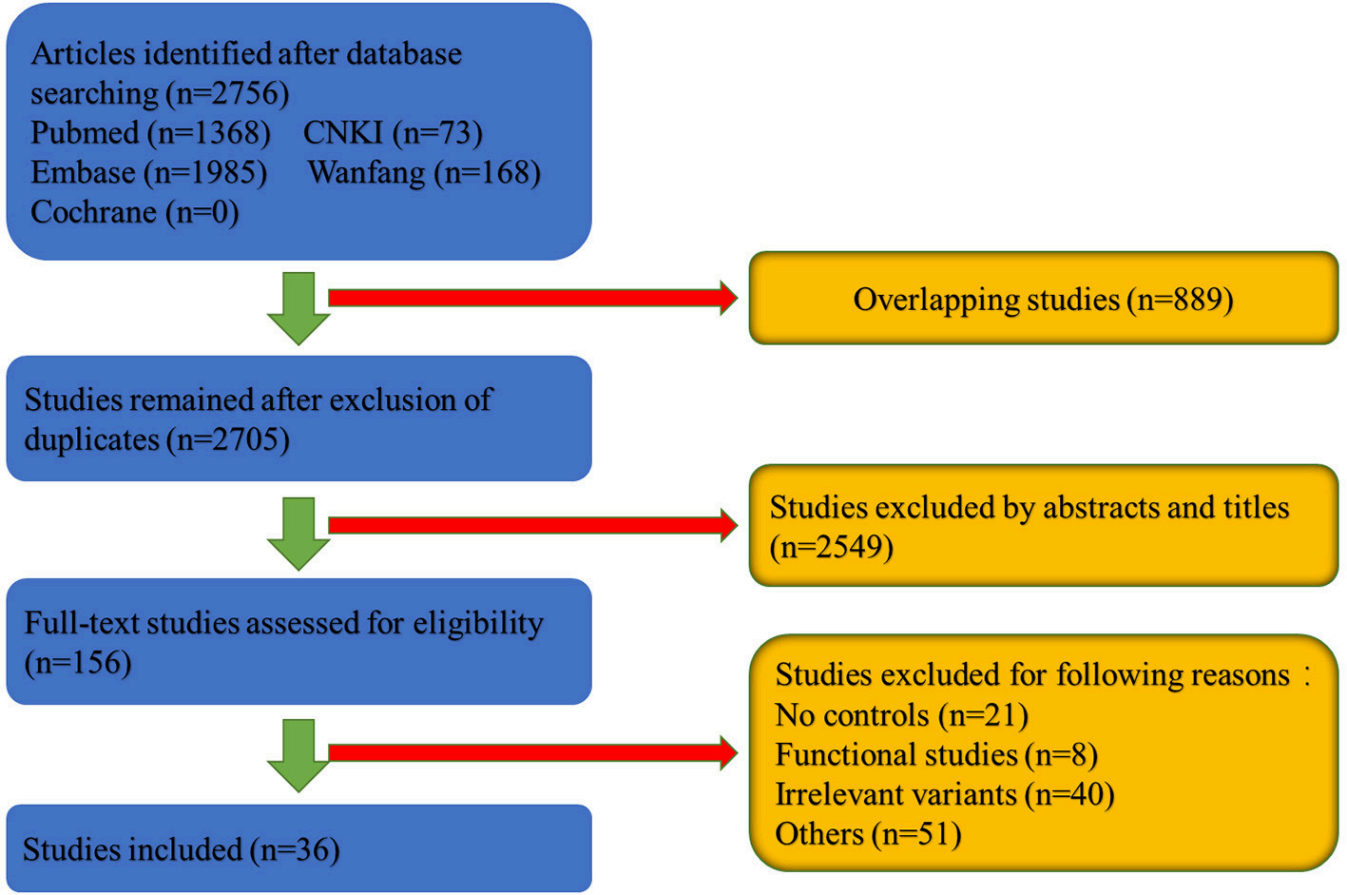
Flowchart of the meta-analysis of *SNCA*.

As can be shown in the flowchart in Figure 1, 2,756 articles were retrieved in the databases search. We excluded 889 overlapping studies and 2,549 articles after reviewing the titles and abstracts. The last step involved excluding 120 articles after full-text review because of lack of controls, functional studies and so on. Finally, 36 articles involving 24,075 cases and 22,877 controls were included in the meta-analysis (Table 1 and Supplementary Table 1).

### Relevant *SNCA* SNPs in the overall populations and subpopulations by ethnicity

**Figure 2:**
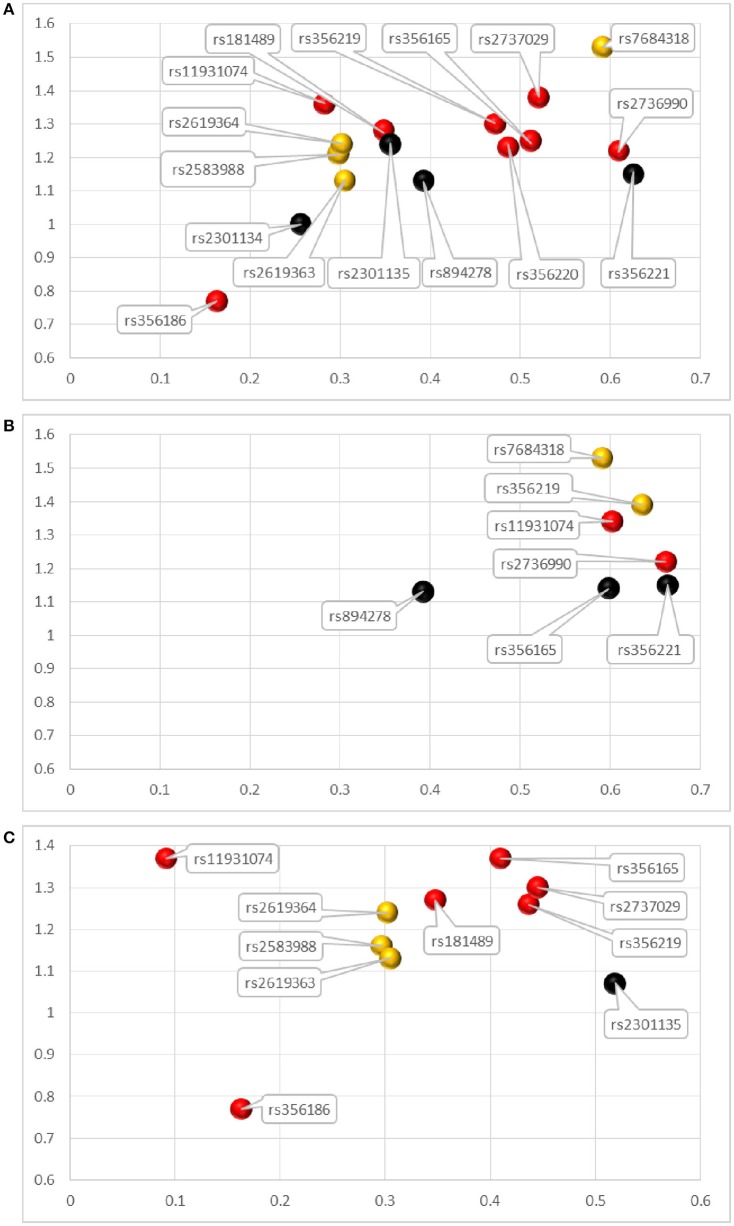
Attributions for *SNCA* variants analyzed in the meta-analysis. **(A–C)** represent the *SNCA* variants conducted pooled analysis in the overall populations, East Asian group and European group, respectively. The horizonal axis represents the allele frequencies (AFs) of variants and vertical axis represents the odds ratios (ORs). The red bubbles represent the most recommended SNPs for the genetic screening of *SNCA*, with *p*< 1 × 10^−5^. The orange bubbles represent the recommended SNPs, with *p* < 0.05. The black bubbles represent SNPs not recommended for genetic screening of *SNCA*, with no meaningful *p-*values.

As can be seen in Table 2, 16 SNPs were included in the meta-analysis. Among them, 12 variants were significant at *p* < 0.05 (rs181489, rs356165, rs356186, rs356219, rs356220, rs2583988, rs2619363, rs2619364, rs2736990, rs2737029, rs7684318, and rs11931074) (Supplementary Figure 1), which we defined as recommended *SNCA* SNPs for genetic screening (Figure 2). We narrowed down the SNPs to the most relevant based on *p* < 1 × 10^−5^. Eight SNPs (rs181489, rs356165, rs356186, rs356219, rs356220, rs2736990, rs2737029, and rs11931074) met this standard and we defined them as the most recommended *SNCA* SNPs (Figure 2). Of these eight SNPs, seven increased the risk of PD (rs2736990, rs356220, rs356165, rs181489, rs356219, rs11931074, and rs2737029 (with ORs of 1.22–1.38 and AFs of 0.6092, 0.4865, 0.5117, 0.3486, 0.4723, 0.2825, and 0.5205, separately) and one decreased the risk of PD (rs356186, with an OR of 0.77 and an AF of 0.1629) in the overall populations. In the subgroup analysis by ethnicity, rs2736990 and rs11931074 increased the risk of PD (with ORs of 1.22 and 1.34, and AFs of 0.6618 and 0.6023) in the East Asian group. Most of the research on *SNCA* variants in East Asians was conducted in China. We carried out a pooled analysis in the Chinese group and found similar results to that in the East Asian group (Supplementary Table 2). In the European group, five SNPs (rs356219, rs181489, rs2737029, rs356165, and rs11931074) increased the risk of PD (with ORs of 1.26–1.37, and AFs of 0.4367, 0.3479, 0.4451, 0.4098, and 0.0917, respectively) while one SNP decreased the risk of PD (rs356186, with an OR of 0.77 and an AF of 0.1629).

**Table 2:** Meta-analysis of the risk alleles of *SNCA* in each group.

**Variants**	**Alleles and related data**		**In total**	**East Asian**	**European**	Mixed^*^	**Latino**	West Asian^*^
**rs181489**	**T**	OR (95%CI)	**1.28 [1.17, 1.40]**	–	**1.27 [1.15, 1.39]**	–	–	–
		*p*-value	<**0.00001**	–	<**0.00001**	–	–	–
		AF (P)	**0.3486**	–	**0.3479**	0.359	–	–
		AF (C)	**0.2974**	–	**0.299**	0.2792	–	–
		AF (G)	**0.2531**	0.0006	**0.3071**	–	0.1866	–
**rs356165**	**G**	OR (95%CI)	**1.25 [1.15, 1.37]**	1.14 [0.97, 1.34]	**1.37 [1.25, 1.50]**	–	–	–
		*p*-value	<**0.00001**	0.11	<**0.00001**	–	–	–
		AF (P)	**0.5117**	0.5982	**0.4098**	0.4512	–	–
		AF (C)	**0.4631**	0.5489	**0.3399**	0.3979	–	–
		AF (G)	**0.4601**	0.5293	**0.3654**	–	0.4688	–
**rs356186**	**A**	OR (95%CI)	**0.77 [0.70, 0.86]**	–	**0.77 [0.70, 0.86]**	–	–	–
		*p*-value	<**0.00001**	–	<**0.00001**	–	–	–
		AF (P)	**0.1629**	–	**0.1629**	–	–	–
		AF (C)	**0.2008**	–	**0.2008**	–	–	–
		AF (G)	**0.194**	0.1559	**0.2**	–	0.244	–
**rs356219**	**G**	OR (95%CI)	**1.30 [1.23, 1.38]**	1.39 [1.15, 1.68]	**1.26 [1.19, 1.34]**	–	–	–
		*p*-value	<**0.00001**	0.0007	<**0.00001**	–	–	–
		AF (P)	**0.4723**	0.6356	**0.4367**	0.452	0.6058	–
		AF (C)	**0.4038**	0.555	**0.3776**	0.3483	0.4745	–
		AF (G)	**0.4549**	0.5276	**0.3654**	–	0.4677	–
**rs356220**	**T**	OR (95%CI)	**1.23 [1.14, 1.33]**	–	–			–
		*p-*value	<**0.00001**	–	–			–
		AF (P)	**0.4865**	0.5907	0.4181	–	–	0.4154
		AF (C)	**0.4363**	0.5543	0.3715	–	–	0.3442
		AF (G)	**0.4543**	0.5224	0.3608	–	0.4513	–
rs356221	A	OR (95%CI)	1.15 [0.94, 1.40]	1.15 [0.85, 1.55]	–	–	–	–
		*p*-value	0.16	0.37	–	–	–	–
		AF (P)	0.6257	0.664	0.4879	–	–	–
		AF (C)	0.5963	0.628	0.459	–	–	–
		AF (G)	0.5591	0.605	0.4584	–	0.5252	–
rs894278	G	OR (95%CI)	1.13 [0.87, 1.46]	1.13 [0.87, 1.46]	–	–	–	–
		*p*-value	0.37	0.37	–	–	–	–
		AF (P)	0.3926	0.3926	–	–	–	–
		AF (C)	0.3626	0.3626	–	–	–	–
		AF (G)	0.1479	0.368	0.0482	–	0.1878	–
rs2301134	A	OR (95%CI)	1.00 [0.74, 1.34]	–	–	–	–	–
		*p*-value	0.97	–	–	–	–	–
		AF (P)	0.2559	0.1245	0.5312	–	–	–
		AF (C)	0.2454	0.1301	0.5066	–	–	–
		AF (G)	0.4248	0.1582	0.4935	–	0.4079	–
rs2301135	G	OR (95%CI)	1.24 [0.87, 1.76]	–	1.07 [0.98, 1.17]	–	–	–
		*p*-value	0.23	–	0.13	–	–	–
		AF (P)	0.3559	0.0873	0.5188	–	–	–
		AF (C)	0.3581	0.0799	0.502	–	–	–
		AF (G)	0.4179	0.1591	0.4944	–	0.406	–
rs2583988	T	OR (95%CI)	1.21 [1.08, 1.35]	–	1.16 [1.04, 1.31]	–	–	–
		*p*-value	0.001	–	0.009	–	–	–
		AF (P)	0.2981	–	0.2968	–	0.2885	–
		AF (C)	0.2648	–	0.2682	–	0.1939	–
		AF (G)	0.1982	0.1495	0.2676	–	0.1495	–
rs2619363	T	OR (95%CI)	1.13 [1.02, 1.24]	–	1.13 [1.02, 1.24]	–	–	–
		*p*-value	0.01	–	0.01	–	–	–
		AF (P)	0.3051	–	0.3051	–	–	–
		AF (C)	0.2802	–	0.2802	–	–	–
		AF (G)	0.1887	0.0006	0.2674	–	0.1504	–
rs2619364	G	OR (95%CI)	1.24 [1.11, 1.40]	–	1.24 [1.11, 1.40]	–	–	–
		*p*-value	0.0003	–	0.0003	–	–	–
		AF (P)	0.302	–	0.302	–	–	–
		AF (C)	0.2587	–	0.2587	–	–	–
		AF (G)	0.1891	0.0006	0.2676	–	0.1495	–
**rs2736990**	**G**	OR (95%CI)	**1.22 [1.13, 1.31]**	**1.22 [1.11, 1.35]**	–	–	–	–
		*p*-value	<**0.00001**	<**0.0001**	–	–	–	–
		AF (P)	**0.6092**	**0.6618**	0.5101	–	0.6587	–
		AF (C)	**0.5602**	**0.6191**	0.47	–	0.5255	–
		AF (G)	**0.554**	**0.6117**	0.457	–	0.524	–
**rs2737029**	**G**	OR (95%CI)	**1.38 [1.20, 1.59]**	–	**1.30 [1.16, 1.45]**	–	–	–
		*p*-value	<**0.00001**	–	<**0.00001**	–	–	–
		AF (P)	**0.5205**	0.6842	0.4451	–	–	–
		AF (C)	**0.4478**	0.5756	0.3839	–	–	–
		AF (G)	**0.4505**	0.5437	0.3903	–	–	–
rs7684318	C	OR (95%CI)	1.53 [1.22, 1.91]	1.53 [1.22, 1.91]	–	–	–	–
		*p*-value	0.0003	0.0003	–	–	–	–
		AF (P)	0.5913	0.5913	–	–	–	–
		AF (C)	0.5229	0.5229	–	–	–	–
		AF (G)	0.1912	0.5318	0.057	–	0.2818	–
**rs11931074**	**T**	OR (95%CI)	**1.36 [1.29, 1.44]**	**1.34 [1.26, 1.44]**	**1.37 [1.24, 1.52]**	–	–	–
		*p*-value	<**0.00001**	<**0.00001**	<**0.00001**	–	–	–
		AF (P)	**0.2825**	**0.6023**	**0.0917**	0.1008	0.2932	0.325
		AF (C)	**0.2501**	**0.5297**	**0.069**	0.076	0.2296	0.2404
		AF (G)	**0.2344**	**0.5281**	**0.0623**	–	0.2936	–

*The bold characters and numbers represent the most recommended variants and corresponding relevant data (p < 1 × 10^−5^)*.

AF, allele frequency; P, patients; C, controls; G, GnomAD database; OR, odds ratio; CI, confidence interval;

* *no related AF in the GnomAD database*.

### Contributions of heterozygotes and homozygotes regarding the relevant SNPs

Regarding the relevant eight *SNCA* SNPs, we explored the contributions of heterozygotes and homozygotes concerning each variant using dominant and recessive models (Table 3 and Supplementary Figures 2, 3). In the overall populations, there were significant differences in the dominant models for rs356186 and rs2737029, which indicated that heterozygotes contributed to the importance of these variants (ORs: 0.74 and 1.52, separately). In the recessive models, rs2736990, rs356220, rs356165, and rs181489 had significant differences, which demonstrated the contributions of homozygotes concerning these variants (ORs: 1.3–1.58). Rs356219 and rs11931074 had significant differences in both models, which demonstrated the importance of both heterozygotes and homozygotes (ORs: 1.34, 1.52; 1.43, and 1.53, separately).

**Table 3:** Meta-analysis of the different models of *SNCA* variants in each group.

**Variants**	**Allele (1/2)**	**Data**	**In total**		**European**		**East Asian**	
			**DM**	**RM**	**DM**	**RM**	**DM**	**RM**
rs181489	T/C	OR[95%CI]	1.29 [1.15, 1.45]	**1.58 [1.39, 1.79]**	1.26 [1.12, 1.42]	**1.58 [1.38, 1.80]**	–	–
		*p*-value	< 0.0001	<**0.00001**	0.0001	<**0.00001**	–	–
rs356165	G/A	OR[95%CI]	1.27 [1.11, 1.46]	**1.48 [1.35, 1.61]**	**1.40 [1.24, 1.59]**	**1.71 [1.43, 2.05]**	1.08 [0.81, 1.45]	**1.42 [1.27, 1.58]**
		*p*-value	0.0004	<**0.00001**	<**0.00001**	<**0.00001**	0.59	<**0.00001**
rs356186	A/G	OR[95%CI]	**0.74 [0.66, 0.83]**	0.76 [0.57, 1.03]	–	–	**0.74 [0.66, 0.83]**	0.76 [0.57, 1.03]
		*p*-value	<**0.00001**	0.08	–	–	<**0.00001**	0.08
rs356219	G/A	OR[95%CI]	**1.34 [1.27, 1.41]**	**1.52 [1.42, 1.62]**	**1.30 [1.22, 1.38]**	**1.47 [1.36, 1.59]**	**1.55 [1.31, 1.83]**	**1.60 [1.40, 1.82]**
		*p*-value	<**0.00001**	<**0.00001**	<**0.00001**	<**0.00001**	<**0.00001**	<**0.00001**
rs356220	T/C	OR[95%CI]	1.20 [1.07, 1.35]	**1.48 [1.30, 1.68]**	–	–	–	–
		*p*-value	0.002	<**0.00001**	–	–	–	–
rs2736990	G/A	OR[95%CI]	1.30 [1.14, 1.49]	**1.30 [1.16, 1.45]**	–	–	1.41 [1.16, 1.71]	1.25 [1.09, 1.43]
		*p*-value	0.0001	<**0.00001**	–	–	0.0006	0.001
rs2737029	G/A	OR[95%CI]	**1.52 [1.27, 1.82]**	1.53 [1.25, 1.87]	1.42 [1.20, 1.69]	1.37 [1.16, 1.62]	–	–
		*p*-value	<**0.00001**	< 0.0001	< 0.0001	0.0003	–	–
rs11931074	T/G	OR[95%CI]	**1.43 [1.33, 1.55]**	**1.53 [1.39, 1.69]**	**1.39 [1.25, 1.55]**	1.64 [1.03, 2.62]	**1.43 [1.27, 1.62]**	**1.52 [1.37, 1.68]**
		*p*-value	<**0.00001**	<**0.00001**	<**0.00001**	0.04	<**0.00001**	<**0.00001**

*The results are presented as OR (95% CI). The bold characters or numbers represent the statistically significant variants and corresponding relevant data*.

*DM, dominant model; RM, recessive model; Allele 1, allele of corresponding variant analyzed in Table 2; OR, odds ratio; CI, confidence interval*.

Regarding the ethnic groups, there were also different results in the dominant and recessive models concerning each variant, which indicated the different contributions of heterozygotes and homozygotes. In the European groups, rs11931074 had a significant difference in the dominant model while rs181489 had a significant difference in the recessive model (ORs: 1.39 and 1.58 separately). Rs356165 and rs356219 had significance differences in both models (ORs: 1.4, 1.71; 1.3 and 1.47). In the East Asian groups, rs356186 had a significant difference in the dominant model while rs356165 had a significant difference in the recessive model (ORs: 0.74 and 1.42). Rs356219 and rs11931074 had significant differences in both models (ORs: 1.55, 1.6; 1.43 and 1.52).

### Sensitivity analysis

The sensitivity analyses were carried out by sequentially deleting each original article. No obvious changes were demonstrated by the pooled OR of each meta-analysis, which indicated that the results were stable.

### Publication bias

Publication bias was investigated using funnel plots in Revman 5.3. Most of the meta-analysis had no publication bias based on the symmetrical shapes of the plots (Supplementary Figures 4–6).

## Discussion

In our meta-analysis, we systematically analyzed the relationship between *SNCA* SNPs and the risk of PD. Our meta-analysis was a comprehensive pooled analysis of *SNCA* SNPs associated with PD risk in the overall populations and subpopulations by ethnicity.

Several SNPs with significant differences were found in our meta-analysis. We found seven SNPs increased the risk of PD (rs2736990, rs356220, rs356165, rs181489, rs356219, rs11931074, and rs2737029) and one decreased the risk of PD (rs356186) in the overall populations. Significant ethnic differences were observed in our meta-analysis. In the East Asian group, rs2736990 and rs11931074 increased the risk of PD. In the European group, five SNPs (rs356219, rs181489, rs2737029, rs356165, and rs11931074) increased the risk of PD while one (rs356186) decreased the risk of PD.

In 2005, genome-wide association studies (GWASs) identified genes such as *SNCA* and *MAPT* as genes involved in PD. In GWAS, *p* < 5 × 10^−8^ was always considered to be the appropriate statistically significant threshold in genome-wide analyses (Nalls et al., 2014). In routine meta-analysis, the statistically significant *p-*value has been defined as < 0.05. Using this cutoff point, we identified 12 significant *SNCA* variants associated with PD risk, which we defined as recommended *SNCA* variants for genetic screening (Figure 2). Just like GWASs that adjusted the *p-*values using the number of whole-genome bases, we considered that when evaluating the PD risks of variants simultaneously, it is wise to use a lower *p-*value as a cutoff point to reduce the false-positive rate and declare variants of a gene to be statistically significant. Therefore, we narrowed down the 12 positive variants using the cutoff point of *p* < 1 × 10^−5^ which was the minimum *p-*value offered in the Revman 5.3 software. Using this cutoff point, we identified eight SNPs as being the most important *SNCA* variants, which we defined as the most recommended *SNCA* variants for genetic screening (Figure 2). Additionally, we explored the contributions of heterozygotes and homozygotes concerning each variant, which could provide evidence for genotype targeting in genetic screening.

In the overall populations, the eight most recommended SNPs (based on our analysis defined by *p-*values) ranked from smallest to largest contribution to PD risk were rs2736990, rs356220, rs356165, rs181489, rs356219, rs11931074, and rs2737029 (with ORs of 1.22–1.38). In the East Asian group, rs2736990 and rs11931074 increased the risk of PD (with ORs of 1.22 and 1.34, separately). In the European group, rs356219, rs181489, rs2737029, rs356165, and rs11931074 increased the risk of PD (with ORs of 1.26–1.37). Rs356186 decreased the risk of PD in the overall populations and the European group (with an OR of 0.77). The ORs were convincing measures of effect size estimates of variants, which represented the variants' contributions to PD risk. In our analysis, our most recommended *SNCA* SNPs only have moderate effect size estimates (with OR < 1.5), which reflected their relatively low contributions to the risk of PD when compared with *GBA* variants (Lill, 2016). Although the ORs associated with *SNCA* SNPs were relatively low, previous research has suggested that functional *SNCA* SNPs can affect *SNCA* expression through epigenetic modification (Soldner et al., 2016), which may allow them to be used as potential therapeutic targets and to contribute to precise treatment strategy design (Carlson, 1990). These polymorphisms in *SNCA* may also possibly affect the risk of PD by damaging the normal function *of* α-synuclein by affecting synaptic activity through modulating the release of synaptic vesicle (Surguchev and Surguchov, 2017). Substitution of amino acids ofα-synuclein changed the gene expression levels including genes functioning in apoptosis, transcription process, membrane proteins, etc. (Baptista et al., 2003). Further functional researches were needed to find the molecular mechanisms in our recommended risk variants of *SNCA*.

**Figure 3:**
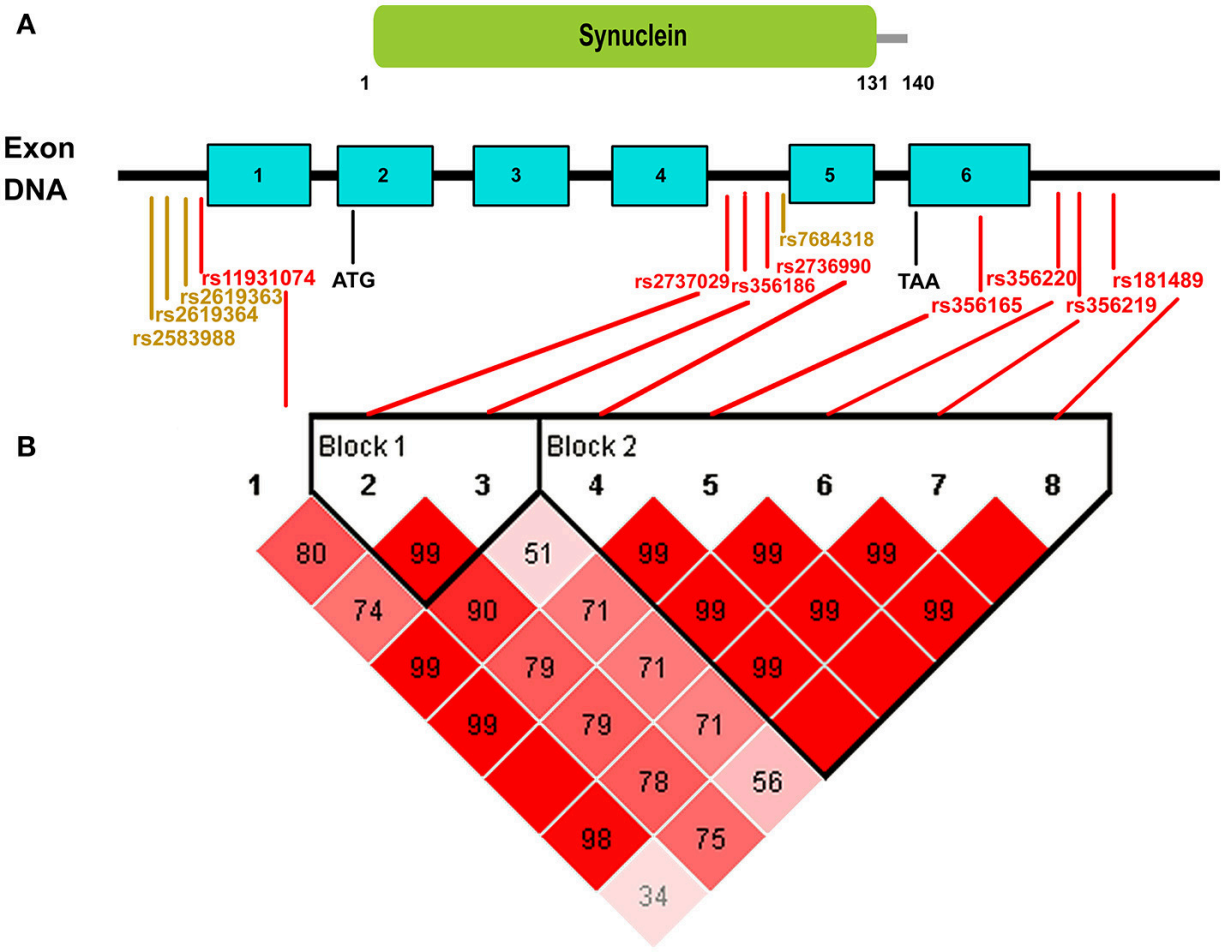
Schematic of the distribution of positive variants in our meta-analysis across the whole *SNCA* gene. **(A)** ATG indicates the start codon and TAA indicates the end codon. *SNCA* contains six exons and all of the positive SNPs (red markers represent the SNPs with *p* < 1 × 10^−5^ and orange markers represent the SNPs with *p* < 0.05) in our analysis were in non-coding regions of *SNCA*. **(B)** In terms of the haplotype analysis with public data from 1000 Genomes Project, the 8 red significant SNPs could be divided into two blocks: the first block contained variants rs2737029 and rs356186, the second block contained variants rs2736990, rs356165, rs356220, rs356219, and rs181489. The variants in the same block indicated that any of the two variants exposed LD.

When developing genetic screening strategies in the future, not only do we need to focus on the most recommended *SNCA* variants in our analysis, but we should also give priority to screening for important risk SNPs that have the highest ORs and AFs in our analysis (Figure 2). From the Figure 2, we can clearly see that among the most recommended SNPs, rs356165, rs2737029, and rs2736990 in the overall populations, rs356165, rs356219, and rs2737029 in the European group, and rs2736990 and rs11931074 in the Asian group had both ORs and AFs that ranked high in our analysis. Risk variants with low AFs should also be paid attention to because these variants are not easily screened out without large-sample research. Additionally, in developing screening strategies, we should also pay attention to SNPs that share the same haplotype in a specific ethnic group. We performed haplotype analysis for the 8 statistically significant variants with *p* < 1 × 10^−5^ in our results. Because we could not extract specific data for both patients and controls from the publications included in our manuscript, public data from 1000 Genomes project (http://www.internationalgenome.org/home) and Haploview software were used for haplotype analysis. The 8 variants could be divided into 2 blocks (Figure 3): the first block was variants rs2737029 and rs356186, the second block was variants rs2736990, rs356165, rs356220, rs356219, and rs181489, which indicate that any of the two variants exposed LD. The haplotype analysis demonstrated a kind of interaction between variants that the variants were in LD and the interaction commonly existed in the same gene and in the close distance. The tagging variants in the haplotype can represent the other variants. For example, a haplotype in the 3′ region of *SNCA* contains rs356219, rs356220, rs356165, and rs356203 in the Caucasian population (Pankratz et al., 2009). When we choose SNPs as markers for genetic screening, we should select relevant representative SNPs that are found in different haplotypes across the entire gene (Myhre et al., 2008).

It has been reported that there were other common interactions among variants in different genes associated with the risk of developing PD. In 2015, Guo et al. (2015) analyzed 16 SNPs in eight genes and/or loci in a large Chinese cohort and found Rep1, rs356165, and rs11931074 in *SNCA* gene, G2385R in *LRRK2* gene, rs4698412 in *BST1* gene, rs1564282 in *PARK17*, and L444P in *GBA* gene have an independent and combined significant relationship with PD. Wang et al. (2012) reported that Rep1 and rs356219 in *SNCA*, rs242562 and rs2435207 in *MAPT*, L444P in *GBA*, rs4273468 in *BST1*, rs823144 in PARK16 significantly modified the *LRRK2*-related risk for PD and the patients' ages at onset (AAOs) in a Chinese cohort consisting of 2013 sporadic PD patients and 1971 controls. Moreover, clinical research has demonstrated that patients with *SNCA* variants had deteriorated cognitive functions (Myhre et al., 2008). In some *SNCA* variant carriers, a more tremor-predominant phenotype and a slower rate of motor progression were also shown to be distinct features (Cooper et al., 2017). When selecting *SNCA* variants for genetic screening, it is important to pay attention to the associated unique clinical features in carriers in order to facilitate the estimation of disease prognosis, the selection of optimum symptomatic treatments and the stratification of patients in clinical trials.

Limitations in our pooled analysis were inevitable. First, some unadjusted factors may have caused bias. For instance, differences among original articles in methods, onset age and gender of cases and controls might have confounded the pooled results. With more original articles, we could deal with this problem using detailed subgroup analysis. Second, the original studies in our analysis were cross-sectional studies. Due to the lack of longitudinal and multicenter studies, it is hard to define *SNCA* variants as independent risk factors of PD. Third, the sample sizes of some SNP analyses were not large enough to reach precise results. More research is needed in the future.

## Conclusion

In summary, we observed eight SNPs that were most associated with PD risk, and there were obvious ethnic differences. Seven SNPs (rs2736990, rs356220, rs356165, rs181489, rs356219, rs11931074, and rs2737029) increased the risk of PD and one decreased the risk of PD in the overall populations. In the East Asian group, rs2736990 and rs11931074 increased the risk of PD. In the European group, five SNPs (rs356219, rs181489, rs2737029, rs356165, and rs11931074) increased the risk of PD while one decreased the risk of PD. Variants with relatively high ORs and AFs in our analysis should be given priority when carrying out genetic screening.

## Author contributions

YZ, LS, and BT conceived and designed the experiments and wrote the manuscript. YZ, LS, and QS performed the experiments. YZ, LS, QS, and BT analyzed the data. HP and JG reference collection and data management.

### Conflict of interest statement

The authors declare that the research was conducted in the absence of any commercial or financial relationships that could be construed as a potential conflict of interest.
