# Supplementary material for: A Comprehensive Analysis of the Association Between SNCA Polymorphisms and the Risk of Parkinson's Disease
Source: Front Mol Neurosci. 2018 Oct 25;11:391. doi: 10.3389/fnmol.2018.00391 (PMC6209653; doi:10.3389/fnmol.2018.00391)
Supplement: Supplementary file 1 [file Table_1.DOCX]

Supplementary Material

A comprehensive analysis of the association between *SNCA* polymorphisms with the risk of Parkinson’s disease

**Yuan Zhang^1 †^, Li Shu^1 †^, Qiying Sun^2,3,4^, Hongxu Pan^1^, Jifeng Guo^1,3,4,6,7,8^, Beisha Tang^1, 2,3,4,5,6,7,8*^**

**^†^** These authors have contributed equally to this work and are co-first authors.

^*^ Correspondence: Beisha Tang [bstang7398@163.com](mailto:bstang7398@163.com)

**Supplementary Table 1**. Genotypes’ attributions of 16 *SNCA* variants in all publications included.

(1)

| Year | Authors | Variants | TT(cases) | TC(cases) | CC(cases) | TT(controls) | TC(controls) | CC(controls) |
| --- | --- | --- | --- | --- | --- | --- | --- | --- |
| 2007 | Ross OA | rs181489 | 28 | 81 | 73 | 23 | 68 | 93 |
| 2007 | Winkler S | rs181489 | 70 | 145 | 180 | 21 | 114 | 134 |
| 2011 | Elbaz A(1) | rs181489 | 99 | 402 | 400 | 58 | 307 | 320 |
| 2011 | Elbaz A(2) | rs181489 | 59 | 221 | 184 | 26 | 135 | 172 |
| 2011 | Elbaz A(3) | rs181489 | 15 | 65 | 80 | 17 | 68 | 91 |
| 2011 | Elbaz A(4) | rs181489 | 143 | 444 | 452 | 61 | 265 | 318 |
| 2011 | Elbaz A(5) | rs181489 | 80 | 233 | 290 | 56 | 228 | 240 |
| 2011 | Elbaz A(6) | rs181489 | 47 | 144 | 158 | 30 | 135 | 171 |
| 2011 | Elbaz A(7) | rs181489 | 57 | 245 | 244 | 11 | 61 | 65 |
| 2011 | Elbaz A(8) | rs181489 | 64 | 193 | 163 | 28 | 160 | 174 |
| 2011 | Elbaz A(9) | rs181489 | 58 | 147 | 140 | 38 | 157 | 229 |
| 2011 | Elbaz A(10) | rs181489 | 50 | 170 | 156 | 32 | 137 | 191 |

(2)

| Year | Authors | Variants | GG(cases) | GA(cases) | AA(cases) | GG(controls) | GA(controls) | AA(controls) |
| --- | --- | --- | --- | --- | --- | --- | --- | --- |
| 2006 | Mizuta I | rs356165 | 379 | 399 | 89 | 289 | 482 | 159 |
| 2010 | Hu FY | rs356165 | 86 | 173 | 71 | 73 | 163 | 64 |
| 2013 | Liu B | rs356165 | 36 | 49 | 31 | 31 | 48 | 16 |
| 2013 | Wu-Chou YH | rs356165 | 204 | 322 | 100 | 121 | 275 | 77 |
| 2015 | Guo JF | rs356165 | 330 | 524 | 165 | 256 | 560 | 214 |
| 2017 | Chen WJ | rs356165 | 78 | 97 | 35 | 93 | 126 | 32 |
| 2007 | Ross OA | rs356165 | 41 | 86 | 53 | 30 | 77 | 74 |
| 2007 | Winkler S | rs356165 | 78 | 171 | 142 | 29 | 122 | 118 |
| 2008 | Myhre R | rs356165 | 47 | 96 | 67 | 29 | 102 | 86 |
| 2009 | Pankratz N | rs356165 | 91 | 212 | 142 | 42 | 172 | 121 |
| 2012 | Cardo LF | rs356165 | 171 | 514 | 450 | 78 | 317 | 377 |
| 2014 | Lee PC | rs356165 | 107 | 285 | 161 | 109 | 339 | 252 |

(3)

| Year | Authors | Variants | AA(cases) | AG(cases) | GG(cases) | AA(controls) | AG(controls) | GG(controls) |
| --- | --- | --- | --- | --- | --- | --- | --- | --- |
| 2007 | Ross OA | rs356186 | 8 | 52 | 123 | 6 | 79 | 97 |
| 2007 | Winkler S | rs356186 | 13 | 103 | 280 | 12 | 82 | 175 |
| 2012 | Trotta L | rs356186 | 25 | 252 | 627 | 45 | 298 | 548 |
| 2009 | Chung SJ | rs356186 | 35 | 267 | 781 | 36 | 317 | 730 |

(4)

| Year | Authors | Variants | GG(cases) | GA(cases) | AA(cases) | GG(controls) | GA(controls) | AA(controls) |
| --- | --- | --- | --- | --- | --- | --- | --- | --- |
| 2012 | Gan R | rs356219 | 69 | 89 | 31 | 59 | 82 | 48 |
| 2012 | Li NN | rs356219 | 312 | 301 | 72 | 148 | 312 | 109 |
| 2012 | Miyake Y | rs356219 | 99 | 98 | 32 | 126 | 175 | 56 |
| 2012 | Pan F | rs356219 | 170 | 179 | 54 | 102 | 142 | 71 |
| 2013 | Wu-Chou YH | rs356219 | 226 | 300 | 107 | 139 | 221 | 84 |
| 2007 | Goris A | rs356219 | 116 | 282 | 251 | 277 | 977 | 869 |
| 2008 | Myhre R | rs356219 | 44 | 94 | 65 | 27 | 106 | 85 |
| 2008 | Westerlund M | rs356219 | 42 | 114 | 89 | 34 | 115 | 101 |
| 2009 | Pankratz N | rs356219 | 91 | 212 | 142 | 42 | 172 | 121 |
| 2010 | Mata IF | rs356219 | 137 | 340 | 205 | 78 | 311 | 275 |
| 2011 | Botta-Orfila T | rs356219 | 132 | 355 | 270 | 71 | 323 | 314 |
| 2011 | Elbaz A(1) | rs356219 | 150 | 458 | 302 | 102 | 337 | 255 |
| 2011 | Elbaz A(2) | rs356219 | 88 | 236 | 147 | 46 | 156 | 157 |
| 2011 | Elbaz A(3) | rs356219 | 17 | 39 | 37 | 25 | 80 | 74 |
| 2011 | Elbaz A(4) | rs356219 | 217 | 518 | 342 | 86 | 313 | 272 |
| 2011 | Elbaz A(5) | rs356219 | 124 | 279 | 198 | 84 | 262 | 180 |
| 2011 | Elbaz A(6) | rs356219 | 64 | 150 | 127 | 40 | 162 | 136 |
| 2011 | Elbaz A(7) | rs356219 | 96 | 283 | 183 | 20 | 70 | 51 |
| 2011 | Elbaz A(8) | rs356219 | 90 | 208 | 152 | 56 | 196 | 152 |
| 2011 | Elbaz A(9) | rs356219 | 77 | 179 | 97 | 106 | 195 | 127 |
| 2011 | Wider C | rs356219 | 307 | 478 | 235 | 241 | 507 | 347 |
| 2012 | Trotta L | rs356219 | 162 | 422 | 320 | 132 | 405 | 354 |
| 2013 | Emelyanov A | rs356219 | 30 | 107 | 87 | 28 | 126 | 154 |
| 2017 | Campêlo CL | rs356219 | 38 | 50 | 16 | 21 | 51 | 26 |
| 2011 | Elbaz A(10) | rs356219 | 80 | 179 | 116 | 44 | 160 | 152 |

(5)

| Year | Authors | Variants | TT(cases) | TC(cases) | CC(cases) | TT(controls) | TC(controls) | CC(controls) |
| --- | --- | --- | --- | --- | --- | --- | --- | --- |
| 2012 | Miyake Y | rs356220 | 100 | 97 | 32 | 126 | 174 | 57 |
| 2014 | Guo XY | rs356220 | 343 | 482 | 186 | 193 | 383 | 145 |
| 2009 | Pankratz N | rs356220 | 91 | 212 | 142 | 42 | 172 | 121 |
| 2012 | Trotta L | rs356220 | 156 | 422 | 326 | 123 | 409 | 359 |
| 2016 | Shahmohammadibeni N | rs356220 | 93 | 246 | 181 | 52 | 254 | 214 |

(6)

| Year | Authors | Variants | AA(cases) | AT(cases) | TT(cases) | AA(controls) | AT(controls) | TT(controls) |
| --- | --- | --- | --- | --- | --- | --- | --- | --- |
| 2006 | Mizuta I | rs356221 | 431 | 369 | 73 | 360 | 449 | 123 |
| 2013 | Wu-Chou YH | rs356221 | 275 | 282 | 69 | 172 | 240 | 61 |
| 2016 | Fang J | rs356221 | 210 | 282 | 91 | 231 | 244 | 78 |
| 2007 | Ross OA | rs356221 | 55 | 84 | 45 | 40 | 89 | 53 |
| 2007 | Winkler S | rs356221 | 99 | 174 | 123 | 56 | 133 | 80 |

(7)

| Year | Authors | Variants | TT(cases) | TG(cases) | GG(cases) | TT(controls) | TG(controls) | GG(controls) |
| --- | --- | --- | --- | --- | --- | --- | --- | --- |
| 2006 | Mizuta I | rs894278 | 275 | 438 | 156 | 375 | 441 | 117 |
| 2010 | Chang XL | rs894278 | 231 | 221 | 52 | 240 | 281 | 102 |
| 2013 | Liu B | rs894278 | 43 | 49 | 24 | 46 | 39 | 10 |
| 2013 | Liu J | rs894278 | 313 | 415 | 125 | 332 | 299 | 97 |
| 2015 | Wu GP | rs894278 | 42 | 55 | 23 | 35 | 44 | 21 |

(8)

| Year | Authors | Variants | GG(cases) | GA(cases) | AA(cases) | GG(controls) | GA(controls) | AA(controls) |
| --- | --- | --- | --- | --- | --- | --- | --- | --- |
| 2013 | Wu-Chou YH | rs2301134 | 492 | 127 | 7 | 339 | 127 | 7 |
| 2016 | Fang J | rs2301134 | 437 | 132 | 14 | 436 | 108 | 9 |
| 2007 | Ross OA | rs2301134 | 47 | 90 | 45 | 43 | 80 | 60 |
| 2007 | Winkler S | rs2301134 | 88 | 181 | 126 | 72 | 137 | 61 |

(9)

| Year | Authors | Variants | GG(cases) | CG(cases) | CC(cases) | GG(controls) | CG(controls) | CC(controls) |
| --- | --- | --- | --- | --- | --- | --- | --- | --- |
| 2013 | Wu-Chou YH | rs2301135 | 4 | 125 | 497 | 13 | 128 | 332 |
| 2016 | Fang J | rs2301135 | 39 | 0 | 544 | 5 | 0 | 548 |
| 2008 | Myhre R | rs2301135 | 68 | 116 | 49 | 67 | 109 | 60 |
| 2009 | Chung SJ | rs2301135 | 306 | 520 | 256 | 286 | 550 | 246 |
| 2010 | Mata IF | rs2301135 | 169 | 347 | 163 | 150 | 327 | 189 |

(10)

| Year | Authors | Variants | TT(cases) | TC(cases) | CC(cases) | TT(controls) | TC(controls) | CC(controls) |
| --- | --- | --- | --- | --- | --- | --- | --- | --- |
| 2007 | Ross OA | rs2583988 | 18 | 69 | 96 | 18 | 64 | 103 |
| 2007 | Winkler S | rs2583988 | 50 | 147 | 199 | 18 | 93 | 158 |
| 2008 | Myhre R | rs2583988 | 23 | 88 | 120 | 21 | 95 | 120 |
| 2011 | Elbaz A(1) | rs2583988 | 1 | 387 | 464 | 56 | 276 | 371 |
| 2011 | Elbaz A(2) | rs2583988 | 43 | 203 | 234 | 19 | 128 | 218 |
| 2011 | Elbaz A(3) | rs2583988 | 12 | 60 | 91 | 15 | 59 | 105 |
| 2011 | Elbaz A(4) | rs2583988 | 120 | 440 | 526 | 49 | 258 | 364 |
| 2011 | Elbaz A(5) | rs2583988 | 74 | 219 | 309 | 57 | 219 | 250 |
| 2011 | Elbaz A(6) | rs2583988 | 33 | 129 | 186 | 27 | 101 | 208 |
| 2011 | Elbaz A(7) | rs2583988 | 53 | 251 | 258 | 8 | 61 | 74 |
| 2011 | Elbaz A(8) | rs2583988 | 45 | 190 | 213 | 34 | 154 | 212 |
| 2011 | Elbaz A(9) | rs2583988 | 38 | 158 | 150 | 33 | 157 | 243 |
| 2012 | Trotta L | rs2583988 | 87 | 383 | 434 | 67 | 354 | 470 |
| 2017 | Campêlo CL | rs2583988 | 11 | 38 | 55 | 1 | 36 | 61 |
| 2011 | Elbaz A(10) | rs2583988 | 40 | 166 | 172 | 22 | 124 | 215 |

(11)

| Year | Authors | Variants | TT(cases) | GT(cases) | GG(cases) | TT(controls) | GT(controls) | GG(controls) |
| --- | --- | --- | --- | --- | --- | --- | --- | --- |
| 2007 | Parsian AJ | rs2619363 | 50 | 195 | 234 | 23 | 81 | 104 |
| 2007 | Ross OA | rs2619363 | 18 | 70 | 95 | 18 | 63 | 102 |
| 2007 | Winkler S | rs2619363 | 51 | 145 | 200 | 18 | 94 | 157 |
| 2008 | Myhre R | rs2619363 | 23 | 90 | 122 | 20 | 95 | 121 |
| 2009 | Chung SJ | rs2619363 | 117 | 433 | 535 | 87 | 445 | 553 |

(12)

| Year | Authors | Variants | GG(cases) | AG(cases) | AA(cases) | GG(controls) | AG(controls) | AA(controls) |
| --- | --- | --- | --- | --- | --- | --- | --- | --- |
| 2007 | Ross OA | rs2619364 | 18 | 71 | 96 | 18 | 64 | 103 |
| 2007 | Winkler S | rs2619364 | 49 | 148 | 199 | 18 | 93 | 157 |
| 2008 | Myhre R | rs2619364 | 25 | 88 | 118 | 24 | 94 | 118 |
| 2010 | Mata IF | rs2619364 | 68 | 273 | 337 | 36 | 256 | 370 |

(13)

| Year | Authors | Variants | GG(cases) | GA(cases) | AA(cases) | GG(controls) | GA(controls) | AA(controls) |
| --- | --- | --- | --- | --- | --- | --- | --- | --- |
| 2012 | Gan R | rs2736990 | 80 | 87 | 22 | 79 | 78 | 32 |
| 2012 | Miyake Y | rs2736990 | 111 | 97 | 21 | 149 | 162 | 46 |
| 2013 | Pan F | rs2736990 | 230 | 230 | 55 | 180 | 196 | 74 |
| 2014 | Guo XY | rs2736990 | 429 | 459 | 123 | 259 | 356 | 106 |
| 2009 | Chung SJ | rs2736990 | 278 | 550 | 256 | 227 | 565 | 292 |
| 2017 | Campêlo CL | rs2736990 | 47 | 43 | 14 | 24 | 55 | 19 |

(14)

| Year | Authors | Variants | GG(cases) | AG(cases) | AA(cases) | GG(controls) | AG(controls) | AA(controls) |
| --- | --- | --- | --- | --- | --- | --- | --- | --- |
| 2006 | Mizuta I | rs2737029 | 402 | 377 | 84 | 297 | 480 | 156 |
| 2008 | Westerlund M | rs2737029 | 54 | 145 | 89 | 38 | 136 | 126 |
| 2010 | Mata IF | rs2737029 | 140 | 338 | 206 | 102 | 300 | 269 |
| 2012 | Trotta L | rs2737029 | 182 | 435 | 287 | 148 | 417 | 326 |

(15)

| Year | Authors | Variants | TT(cases) | TC(cases) | CC(cases) | TT(controls) | TC(controls) | CC(controls) |
| --- | --- | --- | --- | --- | --- | --- | --- | --- |
| 2010 | Yu L | rs7684318 | 176 | 139 | 17 | 225 | 70 | 5 |
| 2012 | Miyake Y | rs7684318 | 31 | 99 | 99 | 57 | 173 | 127 |
| 2013 | Liu B | rs7684318 | 14 | 57 | 30 | 22 | 47 | 25 |
| 2008 | Mizuta I | rs7684318 | 152 | 620 | 598 | 350 | 928 | 618 |

(16)

| Year | Authors | Variants | TT(cases) | TG(cases) | GG(cases) | TT(controls) | TG(controls) | GG(controls) |
| --- | --- | --- | --- | --- | --- | --- | --- | --- |
| 2012 | Hu Y | rs11931074 | 49 | 41 | 20 | 32 | 83 | 21 |
| 2013 | Liu J | rs11931074 | 268 | 324 | 115 | 190 | 342 | 162 |
| 2013 | Wu-Chou YH | rs11931074 | 229 | 301 | 103 | 129 | 228 | 87 |
| 2015 | Chen YP | rs11931074 | 464 | 600 | 205 | 225 | 437 | 178 |
| 2015 | Guo JF | rs11931074 | 341 | 532 | 148 | 262 | 545 | 225 |
| 2015 | Wu GP | rs11931074 | 49 | 52 | 19 | 43 | 42 | 15 |
| 2007 | Ross OA | rs11931074 | 1 | 30 | 154 | 2 | 16 | 165 |
| 2007 | Winkler S | rs11931074 | 15 | 164 | 909 | 4 | 77 | 592 |
| 2011 | Elbaz A(1) | rs11931074 | 6 | 140 | 773 | 4 | 94 | 608 |
| 2011 | Elbaz A(2) | rs11931074 | 5 | 82 | 392 | 1 | 52 | 308 |
| 2011 | Elbaz A(3) | rs11931074 | 2 | 35 | 125 | 0 | 28 | 152 |
| 2011 | Elbaz A(4) | rs11931074 | 15 | 164 | 909 | 4 | 77 | 592 |
| 2011 | Elbaz A(5) | rs11931074 | 5 | 126 | 461 | 2 | 78 | 439 |
| 2011 | Elbaz A(6) | rs11931074 | 2 | 49 | 296 | 3 | 39 | 297 |
| 2011 | Elbaz A(7) | rs11931074 | 4 | 94 | 463 | 1 | 18 | 124 |
| 2011 | Elbaz A(8) | rs11931074 | 2 | 75 | 374 | 2 | 58 | 345 |
| 2011 | Elbaz A(9) | rs11931074 | 2 | 65 | 291 | 3 | 49 | 392 |
| 2017 | Campêlo CL | rs11931074 | 11 | 39 | 54 | 8 | 29 | 61 |
| 2011 | Elbaz A(10) | rs11931074 | 3 | 70 | 304 | 4 | 47 | 311 |
| 2016 | Shahmohammadibeni N | rs11931074 | 56 | 226 | 238 | 32 | 186 | 302 |
