# Supplementary material for: A Comprehensive Analysis of the Association Between SNCA Polymorphisms and the Risk of Parkinson's Disease
Source: Front Mol Neurosci. 2018 Oct 25;11:391. doi: 10.3389/fnmol.2018.00391 (PMC6209653; doi:10.3389/fnmol.2018.00391)
Supplement: Supplementary file 2 [file Table_2.DOCX]

Supplementary Material

A comprehensive analysis of the association between *SNCA* polymorphisms with the risk of Parkinson’s disease

**Yuan Zhang^1 †^, Li Shu^1 †^, Qiying Sun^2,3,4^, Hongxu Pan^1^, Jifeng Guo^1,3,4,6,7,8^, Beisha Tang^1, 2,3,4,5,6,7,8*^**

**^†^** These authors have contributed equally to this work and are co-first authors.

^*^ Correspondence: Beisha Tang [bstang7398@163.com](mailto:bstang7398@163.com)

**Supplementary Table 2.** The meta-analysis of variants’ risk alleles and different models for *SNCA* in Chinese

Abbreviations: DM: dominant model; RM: recessive model; Allele 1: the analyzed allele of corresponding variant in Table 2. The results were presented in OR (95%CI). OR: odd ratio, 95%CI: 95% confidence interval. The bold characters or numbers represented the statistically significant variants and its relevant data.

| Variants | Allele | Data | Risk Alleles | DM | RM |
| --- | --- | --- | --- | --- | --- |
| rs356165 | G | OR[95%CI] | 1.08 [0.93, 1.25] | **-** | **-** |
|  |  | *p* value | 0.32 | **-** | **-** |
| rs356219 | G | OR[95%CI] | **1.43 [1.14, 1.79]** | 0.88 [0.69, 1.12] | **1.56 [1.11, 2.20]** |
|  |  | *p* value | **0.002** | 0.29 | **0.01** |
| rs894278 | G | OR[95%CI] | 1.08 [0.77, 1.51] | 1.13 [0.91, 1.40] | 1.00 [0.62, 1.61] |
|  |  | *p* value | 0.67 | 0.28 | 0.99 |
| rs2736990 | G | OR[95%CI] | **1.22 [1.10, 1.35]** | 0.98 [0.80, 1.18] | **1.24 [1.07, 1.43]** |
|  |  | *p* value | **0.0002** | 0.80 | **0.004** |
| rs11931074 | T | OR[95%CI] | **1.34 [1.26, 1.44]** | **0.86 [0.79, 0.95]** | **1.52 [1.37, 1.68]** |
|  |  | *p* value | **<0.00001** | **0.002** | **<0.00001** |
