# Supplementary material for: A Comprehensive Analysis of the Association Between SNCA Polymorphisms and the Risk of Parkinson's Disease
Source: Front Mol Neurosci. 2018 Oct 25;11:391. doi: 10.3389/fnmol.2018.00391 (PMC6209653; doi:10.3389/fnmol.2018.00391)
Supplement: Supplementary file 3 [file Image_1.PDF]

Supplementary Material

A comprehensive analysis of the association between *SNCA* polymorphisms with the risk of Parkinson's disease

Yuan Zhang[1 †], Li Shu[1 †], Qiying Sun[2,3,4], Hongxu Pan[1], Jifeng Guo[1,3,4,6,7,8], Beisha Tang[1, 2,3,4,5,6,7,8*]

[†] These authors have contributed equally to this work and are co-first authors.

[*] **Correspondence: Beisha Tang bstang7398@163.com**

(A)

Study or Subgroup	Experimental Events	Experimental Total	Control Events	Control Total	Weight	Odds Ratio M-H, Random, 95% CI
**1.1.1 European**						
2007Ross OA	137	364	114	368	5.6%	1.34 [0.99, 1.83]
2007Winkler S	285	790	156	538	7.7%	1.38 [1.09, 1.75]
2011Elbaz A(1)	600	1802	423	1370	11.5%	1.12 [0.96, 1.30]
2011Elbaz A(2)	339	928	187	666	8.5%	1.47 [1.19, 1.83]
2011Elbaz A(3)	95	320	102	352	5.0%	1.03 [0.74, 1.44]
2011Elbaz A(4)	730	2078	387	1288	11.6%	1.26 [1.09, 1.46]
2011Elbaz A(5)	393	1206	340	1048	10.2%	1.01 [0.84, 1.20]
2011Elbaz A(6)	238	698	195	672	8.0%	1.27 [1.01, 1.59]
2011Elbaz A(7)	359	1092	83	274	6.1%	1.13 [0.85, 1.50]
2011Elbaz A(8)	321	840	216	724	8.7%	1.45 [1.18, 1.80]
2011Elbaz A(9)	263	690	233	848	8.5%	1.63 [1.31, 2.02]
**Subtotal (95% CI)**		**10808**		**8148**	**91.7%**	**1.27 [1.15, 1.39]**
Total events	3760		2436			
Heterogeneity: Tau² = 0.01; Chi² = 20.51, df = 10 (P = 0.02); I² = 51%						
Test for overall effect: Z = 5.00 (P < 0.00001)						
**1.1.2 Mixed**						
2011Elbaz A(10)	270	752	201	720	8.3%	1.45 [1.16, 1.80]
**Subtotal (95% CI)**		**752**		**720**	**8.3%**	**1.45 [1.16, 1.80]**
Total events	270		201			
Heterogeneity: Not applicable						
Test for overall effect: Z = 3.28 (P = 0.001)						
**Total (95% CI)**		**11560**		**8868**	**100.0%**	**1.28 [1.17, 1.40]**
Total events	4030		2637			
Heterogeneity: Tau² = 0.01; Chi² = 21.98, df = 11 (P = 0.02); I² = 50%						
Test for overall effect: Z = 5.52 (P < 0.00001)						
Test for subgroup differences: Chi² = 1.17, df = 1 (P = 0.28), I² = 14.3%						

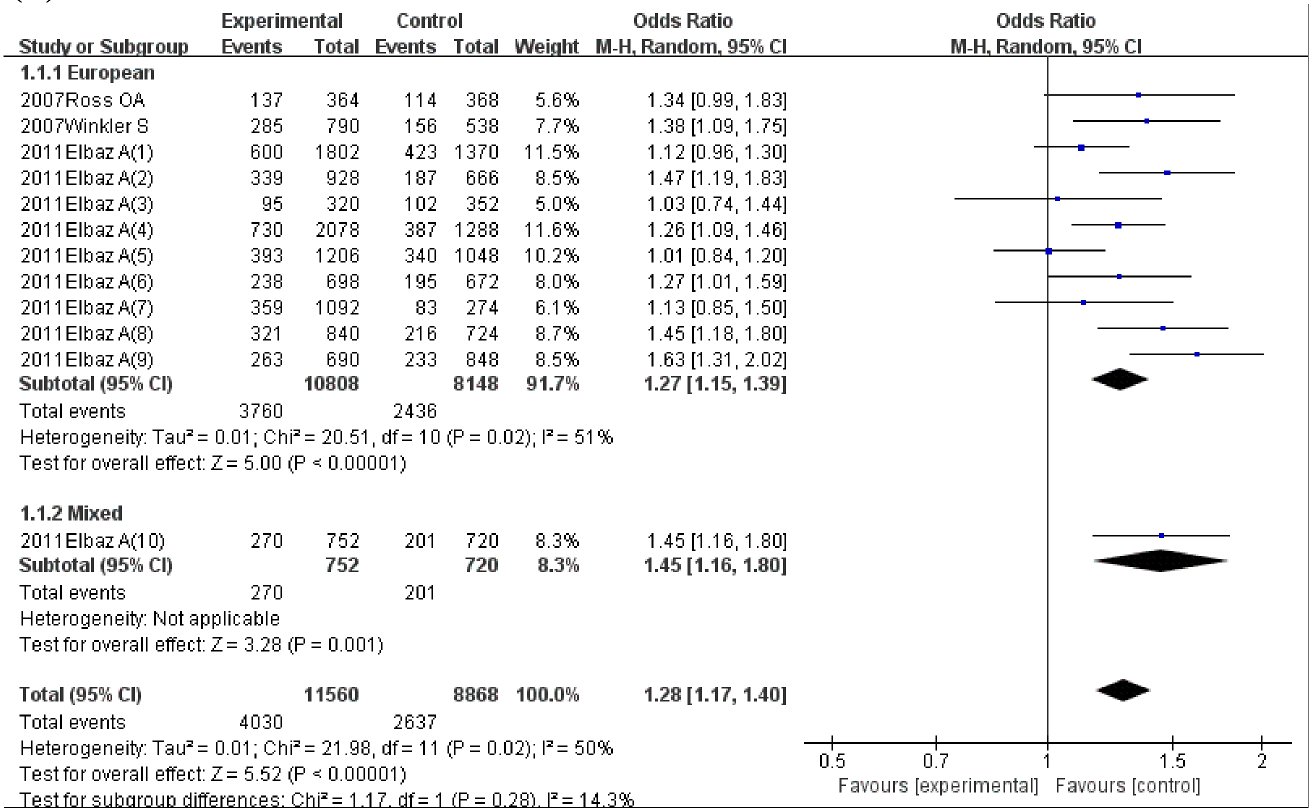

(B)

Study or Subgroup	Experimental Events	Experimental Total	Control Events	Control Total	Weight	Odds Ratio M-H, Random, 95% CI
**1.2.1 East Asian**						
2006Mizuta I	1157	1734	1060	1860	11.4%	1.51 [1.32, 1.73]
2010Hu FY	345	660	309	600	7.7%	1.03 [0.83, 1.29]
2013Liu B	121	232	110	190	3.8%	0.79 [0.54, 1.17]
2013Wu-Chou YH	730	1252	517	946	9.8%	1.16 [0.98, 1.38]
2015Guo JF	1184	2038	1072	2060	12.0%	1.28 [1.13, 1.45]
2017Chen WJ	253	420	312	502	6.3%	0.92 [0.71, 1.20]
**Subtotal (95% CI)**		**6336**		**6158**	**51.0%**	**1.14 [0.97, 1.34]**
Total events	3790		3380			
Heterogeneity: Tau² = 0.03; Chi² = 21.65, df = 5 (P = 0.0006); I² = 77%						
Test for overall effect: Z = 1.60 (P = 0.11)						
**1.2.2 European**						
2007Ross OA	168	360	137	362	5.5%	1.44 [1.07, 1.93]
2007Winkler S	327	782	180	538	7.5%	1.43 [1.14, 1.80]
2008Myhre R	190	420	160	434	6.1%	1.41 [1.08, 1.86]
2009Pankratz N	394	890	256	670	8.4%	1.28 [1.05, 1.58]
2012Cardo LF	856	2270	473	1544	11.3%	1.37 [1.19, 1.57]
**Subtotal (95% CI)**		**4722**		**3548**	**38.7%**	**1.37 [1.25, 1.50]**
Total events	1935		1206			
Heterogeneity: Tau² = 0.00; Chi² = 0.66, df = 4 (P = 0.96); I² = 0%						
Test for overall effect: Z = 6.83 (P < 0.00001)						
**1.2.3 Mixed**						
2014Lee PC	499	1106	557	1400	10.3%	1.24 [1.06, 1.46]
**Subtotal (95% CI)**		**1106**		**1400**	**10.3%**	**1.24 [1.06, 1.46]**
Total events	499		557			
Heterogeneity: Not applicable						
Test for overall effect: Z = 2.68 (P = 0.007)						
**Total (95% CI)**		**12164**		**11106**	**100.0%**	**1.25 [1.15, 1.37]**
Total events	6224		5143			
Heterogeneity: Tau² = 0.01; Chi² = 25.58, df = 11 (P = 0.007); I² = 57%						
Test for overall effect: Z = 5.17 (P < 0.00001)						
Test for subgroup differences: Chi² = 4.18, df = 2 (P = 0.12), I² = 52.2%						

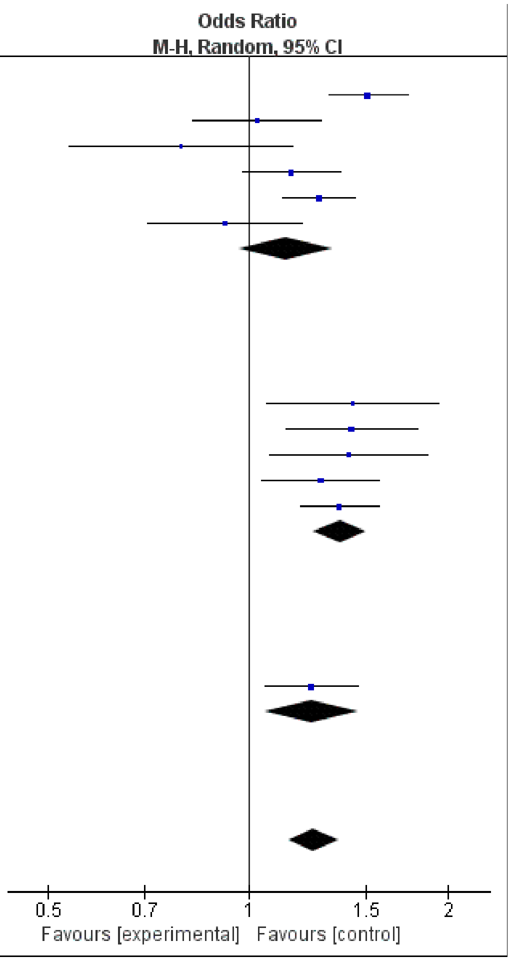

(C)

Study or Subgroup	Experimental Events	Experimental Total	Control Events	Control Total	Weight	Odds Ratio M-H, Fixed, 95% CI
**1.3.1 European**						
2007Ross OA	68	366	91	364	8.9%	0.68 [0.48, 0.98]
2007Winkler S	129	792	106	538	12.7%	0.79 [0.60, 1.05]
2009Chung SJ	302	1808	388	1782	39.0%	0.72 [0.61, 0.85]
2012Trotta L	337	2166	389	2166	39.4%	0.84 [0.72, 0.99]
**Subtotal (95% CI)**		**5132**		**4850**	**100.0%**	**0.77 [0.70, 0.86]**
Total events	836		974			
Heterogeneity: Chi² = 2.25, df = 3 (P = 0.52); I² = 0%						
Test for overall effect: Z = 4.90 (P < 0.00001)						
**Total (95% CI)**		**5132**		**4850**	**100.0%**	**0.77 [0.70, 0.86]**
Total events	836		974			
Heterogeneity: Chi² = 2.25, df = 3 (P = 0.52); I² = 0%						
Test for overall effect: Z = 4.90 (P < 0.00001)						
Test for subgroup differences: Not applicable						

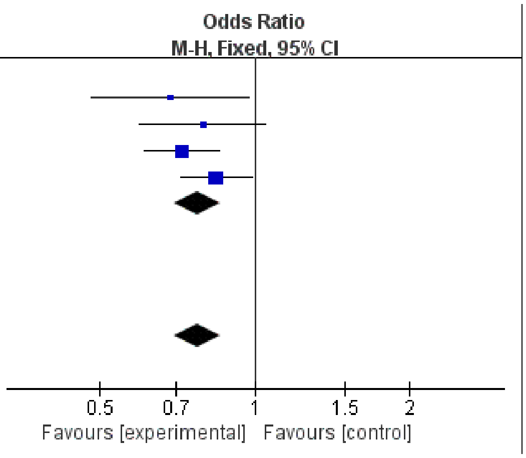

(D)

Study or Subgroup	Experimental Events	Experimental Total	Control Events	Control Total	Weight	Odds Ratio M-H, Random, 95% CI
**1.4.1 East Asian**						
2012Gan R	227	378	200	378	2.7%	1.34 [1.00, 1.79]
2012Li NN	925	1370	608	1138	4.8%	1.81 [1.54, 2.13]
2012Miyake Y	296	458	427	714	3.3%	1.23 [0.96, 1.57]
2012Pan F	519	806	346	630	3.8%	1.48 [1.20, 1.84]
2013Wu-Chou YH	752	1266	499	888	4.5%	1.14 [0.96, 1.36]
**Subtotal (95% CI)**		**4278**		**3748**	**19.1%**	**1.39 [1.15, 1.68]**
Total events	2719		2080			
Heterogeneity: Tau² = 0.03; Chi² = 16.48, df = 4 (P = 0.002); I² = 76%						
Test for overall effect: Z = 3.40 (P = 0.0007)						
**1.4.2 European**						
2007Goris A	514	1298	1531	4246	5.6%	1.16 [1.02, 1.32]
2008Myhre R	182	406	160	436	2.8%	1.40 [1.06, 1.85]
2008Westerlund M	198	490	183	500	3.1%	1.17 [0.91, 1.52]
2009Pankratz N	394	890	256	670	3.9%	1.28 [1.05, 1.58]
2010Mata IF	614	1364	467	1328	4.9%	1.51 [1.29, 1.76]
2011Botta-Orfila T	619	1514	465	1416	5.0%	1.41 [1.22, 1.65]
2011Elbaz A(1)	758	1820	541	1388	5.2%	1.12 [0.97, 1.29]
2011Elbaz A(2)	412	942	248	718	4.0%	1.47 [1.21, 1.80]
2011Elbaz A(3)	73	186	130	358	1.9%	1.13 [0.79, 1.63]
2011Elbaz A(4)	952	2154	485	1342	5.3%	1.40 [1.22, 1.61]
2011Elbaz A(5)	527	1202	430	1052	4.7%	1.13 [0.96, 1.34]
2011Elbaz A(6)	278	682	242	676	3.7%	1.23 [0.99, 1.54]
2011Elbaz A(7)	475	1124	110	282	2.9%	1.14 [0.88, 1.49]
2011Elbaz A(8)	388	900	308	808	4.1%	1.23 [1.01, 1.49]
2011Elbaz A(9)	333	706	407	856	4.0%	0.98 [0.81, 1.20]
2011Wider C	1092	2040	989	2190	5.7%	1.40 [1.24, 1.58]
2012Trotta L	746	1808	669	1782	5.4%	1.17 [1.02, 1.34]
2013Emelyanov A	167	448	182	616	3.1%	1.42 [1.09, 1.83]
**Subtotal (95% CI)**		**19974**		**20664**	**75.4%**	**1.26 [1.19, 1.34]**
Total events	8722		7803			
Heterogeneity: Tau² = 0.01; Chi² = 30.31, df = 17 (P = 0.02); I² = 44%						
Test for overall effect: Z = 8.01 (P < 0.00001)						
**1.4.3 Latino**						
2017Campêlo CL	126	208	93	196	1.7%	1.70 [1.15, 2.53]
**Subtotal (95% CI)**		**208**		**196**	**1.7%**	**1.70 [1.15, 2.53]**
Total events	126		93			
Heterogeneity: Not applicable						
Test for overall effect: Z = 2.64 (P = 0.008)						
**1.4.4 Mixed**						
2011Elbaz A(10)	339	750	248	712	3.8%	1.54 [1.25, 1.91]
**Subtotal (95% CI)**		**750**		**712**	**3.8%**	**1.54 [1.25, 1.91]**
Total events	339		248			
Heterogeneity: Not applicable						
Test for overall effect: Z = 4.03 (P < 0.0001)						
**Total (95% CI)**		**25210**		**25320**	**100.0%**	**1.30 [1.23, 1.38]**
Total events	11906		10224			
Heterogeneity: Tau² = 0.01; Chi² = 56.16, df = 24 (P = 0.0002); I² = 57%						
Test for overall effect: Z = 8.85 (P < 0.00001)						
Test for subgroup differences: Chi² = 5.75, df = 3 (P = 0.12), I² = 47.8%						

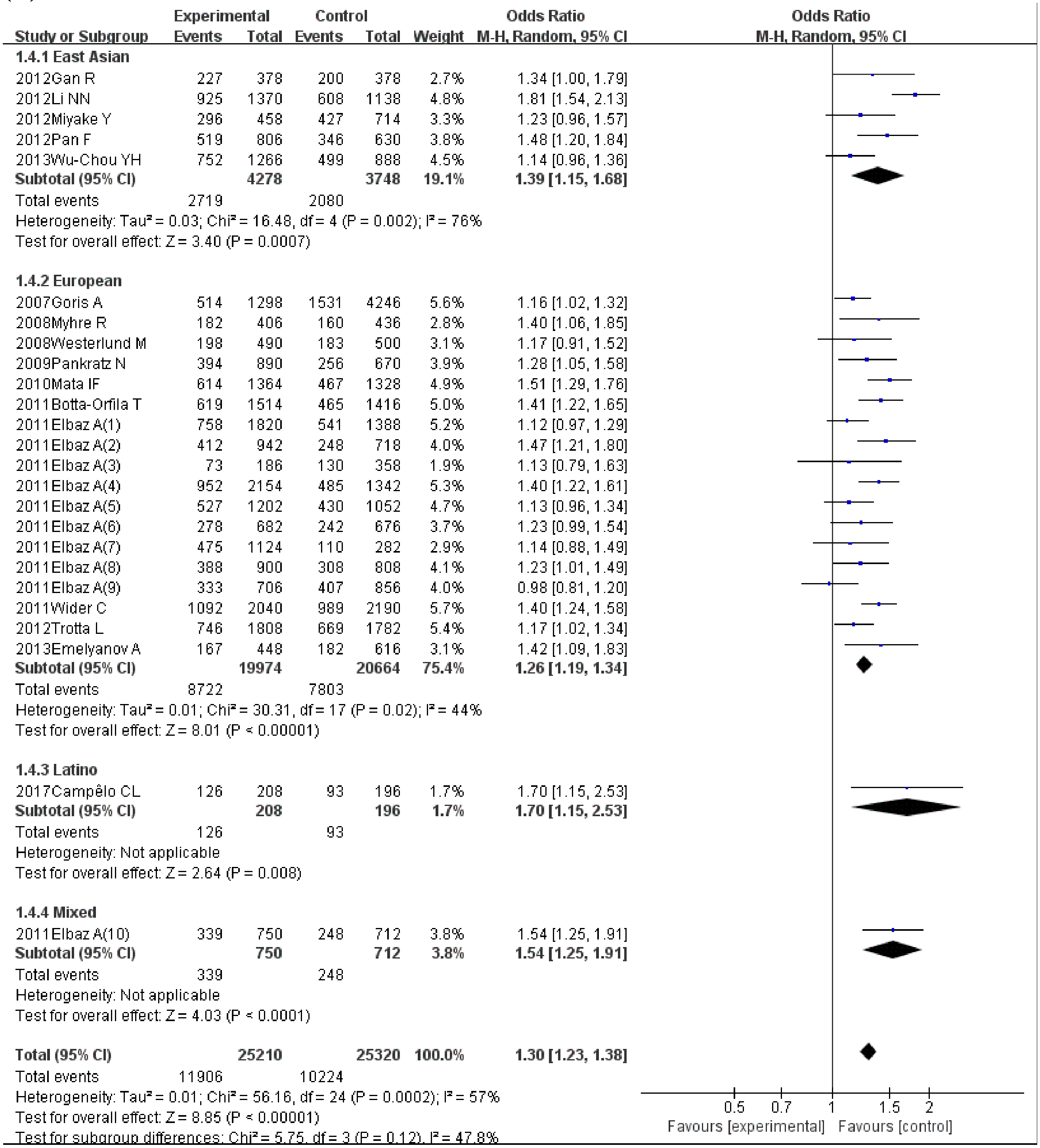

(E)

Study or Subgroup	Experimental Events	Experimental Total	Control Events	Control Total	Weight	Odds Ratio M-H, Fixed, 95% CI
**1.5.1 East Asian**						
2012Miyake Y	297	458	426	714	9.3%	1.25 [0.98, 1.59]
2014Guo XY	1168	2022	769	1442	30.1%	1.20 [1.04, 1.37]
**Subtotal (95% CI)**		**2480**		**2156**	**39.4%**	**1.21 [1.07, 1.36]**
Total events	1465		1195			
Heterogeneity: Chi² = 0.08, df = 1 (P = 0.77); I² = 0%						
Test for overall effect: Z = 3.13 (P = 0.002)						
**1.5.2 European**						
2009Pankratz N	394	890	256	670	12.9%	1.28 [1.05, 1.58]
2012Trotta L	734	1808	655	1782	31.1%	1.18 [1.03, 1.35]
**Subtotal (95% CI)**		**2698**		**2452**	**44.0%**	**1.21 [1.08, 1.35]**
Total events	1128		911			
Heterogeneity: Chi² = 0.50, df = 1 (P = 0.48); I² = 0%						
Test for overall effect: Z = 3.29 (P = 0.0010)						
**1.5.3 West Asian**						
2016Shahmohammadibeni N	432	1040	358	1040	16.6%	1.35 [1.13, 1.62]
**Subtotal (95% CI)**		**1040**		**1040**	**16.6%**	**1.35 [1.13, 1.62]**
Total events	432		358			
Heterogeneity: Not applicable						
Test for overall effect: Z = 3.34 (P = 0.0008)						
**Total (95% CI)**		**6218**		**5648**	**100.0%**	**1.23 [1.14, 1.33]**
Total events	3025		2464			
Heterogeneity: Chi² = 1.88, df = 4 (P = 0.76); I² = 0%						
Test for overall effect: Z = 5.53 (P < 0.00001)						
Test for subgroup differences: Chi² = 1.30, df = 2 (P = 0.52), I² = 0%						

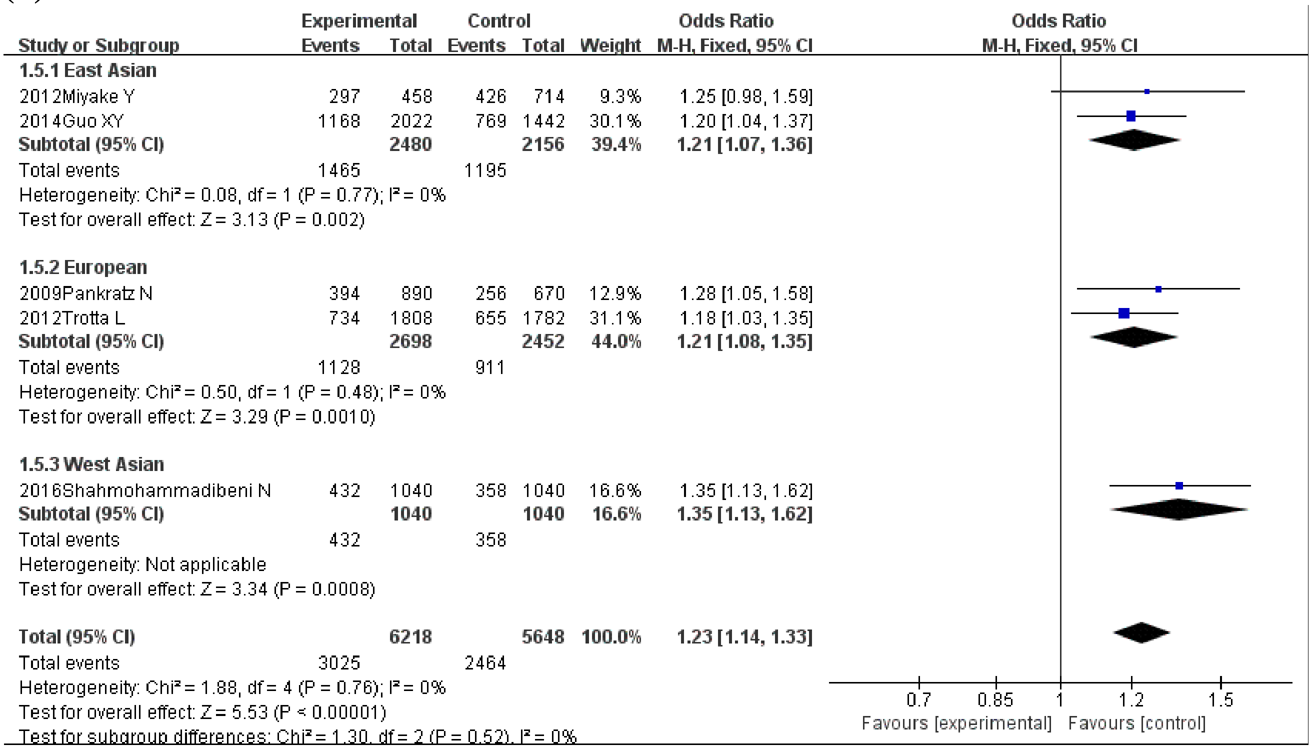

(F)

Study or Subgroup	Experimental Events	Experimental Total	Control Events	Control Total	Weight	Odds Ratio M-H, Random, 95% CI
**1.6.1 East Asian**						
2006Mizuta I	1231	1746	1169	1864	22.3%	1.42 [1.24, 1.63]
2013Wu-Chou YH	832	1252	584	946	21.0%	1.23 [1.03, 1.46]
2016Fang J	702	1166	706	1106	21.2%	0.86 [0.72, 1.02]
**Subtotal (95% CI)**		**4164**		**3916**	**64.5%**	**1.15 [0.85, 1.55]**
Total events	2765		2459			
Heterogeneity: Tau² = 0.06; Chi² = 20.67, df = 2 (P < 0.0001); I² = 90%						
Test for overall effect: Z = 0.90 (P = 0.37)						
**1.6.2 European**						
2007Ross OA	194	368	169	364	16.3%	1.29 [0.96, 1.72]
2007Winkler S	372	792	245	538	19.2%	1.06 [0.85, 1.32]
**Subtotal (95% CI)**		**1160**		**902**	**35.5%**	**1.14 [0.95, 1.37]**
Total events	566		414			
Heterogeneity: Tau² = 0.00; Chi² = 1.09, df = 1 (P = 0.30); I² = 9%						
Test for overall effect: Z = 1.39 (P = 0.17)						
**Total (95% CI)**		**5324**		**4818**	**100.0%**	**1.15 [0.94, 1.40]**
Total events	3331		2873			
Heterogeneity: Tau² = 0.04; Chi² = 21.89, df = 4 (P = 0.0002); I² = 82%						
Test for overall effect: Z = 1.40 (P = 0.16)						
Test for subgroup differences: Chi² = 0.00, df = 1 (P = 0.97), I² = 0%						

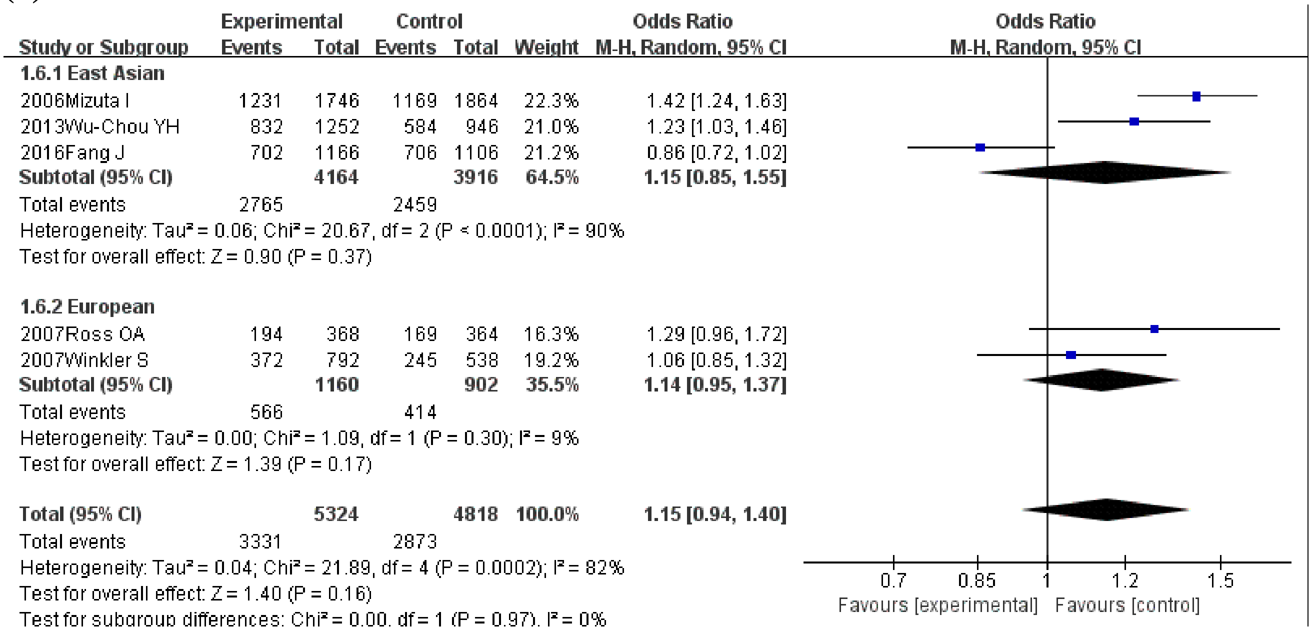

(G)

Study or Subgroup	Experimental Events	Experimental Total	Control Events	Control Total	Weight	Odds Ratio M-H, Random, 95% CI
**1.7.1 East Asian**						
2006Mizuta I	750	1738	675	1866	23.2%	1.34 [1.17, 1.53]
2010Chang XL	325	1008	485	1246	22.2%	0.75 [0.63, 0.89]
2013Liu B	97	232	59	190	15.5%	1.60 [1.07, 2.39]
2013Liu J	665	1706	493	1456	22.9%	1.25 [1.08, 1.44]
2015Wu GP	101	240	86	200	16.2%	0.96 [0.66, 1.41]
**Subtotal (95% CI)**		**4924**		**4958**	**100.0%**	**1.13 [0.87, 1.46]**
Total events	1938		1798			
Heterogeneity: Tau² = 0.07; Chi² = 33.09, df = 4 (P < 0.00001); I² = 88%						
Test for overall effect: Z = 0.90 (P = 0.37)						
**Total (95% CI)**		**4924**		**4958**	**100.0%**	**1.13 [0.87, 1.46]**
Total events	1938		1798			
Heterogeneity: Tau² = 0.07; Chi² = 33.09, df = 4 (P < 0.00001); I² = 88%						
Test for overall effect: Z = 0.90 (P = 0.37)						
Test for subgroup differences: Not applicable						

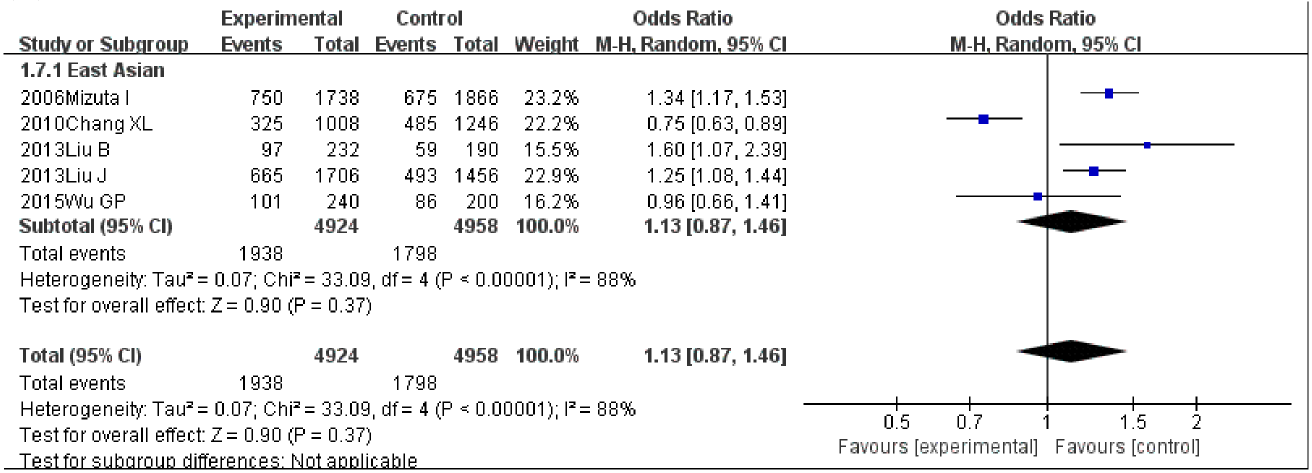

(H)

Study or Subgroup	Experimental Events	Experimental Total	Control Events	Control Total	Weight	Odds Ratio M-H, Random, 95% CI
**1.8.1 East Asian**						
2013Wu-Chou YH	141	1252	141	946	25.1%	0.72 [0.56, 0.93]
2016Fang J	160	1166	126	1106	25.1%	1.24 [0.96, 1.59]
**Subtotal (95% CI)**		**2418**		**2052**	**50.2%**	**0.95 [0.56, 1.60]**
Total events	301		267			
Heterogeneity: Tau² = 0.13; Chi² = 8.80, df = 1 (P = 0.003); I² = 89%						
Test for overall effect: Z = 0.20 (P = 0.84)						
**1.8.2 European**						
2007Ross OA	180	364	200	366	23.6%	0.81 [0.61, 1.09]
2007Winkler S	433	790	259	540	26.2%	1.32 [1.06, 1.64]
**Subtotal (95% CI)**		**1154**		**906**	**49.8%**	**1.04 [0.65, 1.67]**
Total events	613		459			
Heterogeneity: Tau² = 0.10; Chi² = 6.75, df = 1 (P = 0.009); I² = 85%						
Test for overall effect: Z = 0.18 (P = 0.86)						
**Total (95% CI)**		**3572**		**2958**	**100.0%**	**1.00 [0.74, 1.34]**
Total events	914		726			
Heterogeneity: Tau² = 0.08; Chi² = 17.00, df = 3 (P = 0.0007); I² = 82%						
Test for overall effect: Z = 0.03 (P = 0.97)						
Test for subgroup differences: Chi² = 0.07, df = 1 (P = 0.79), I² = 0%						

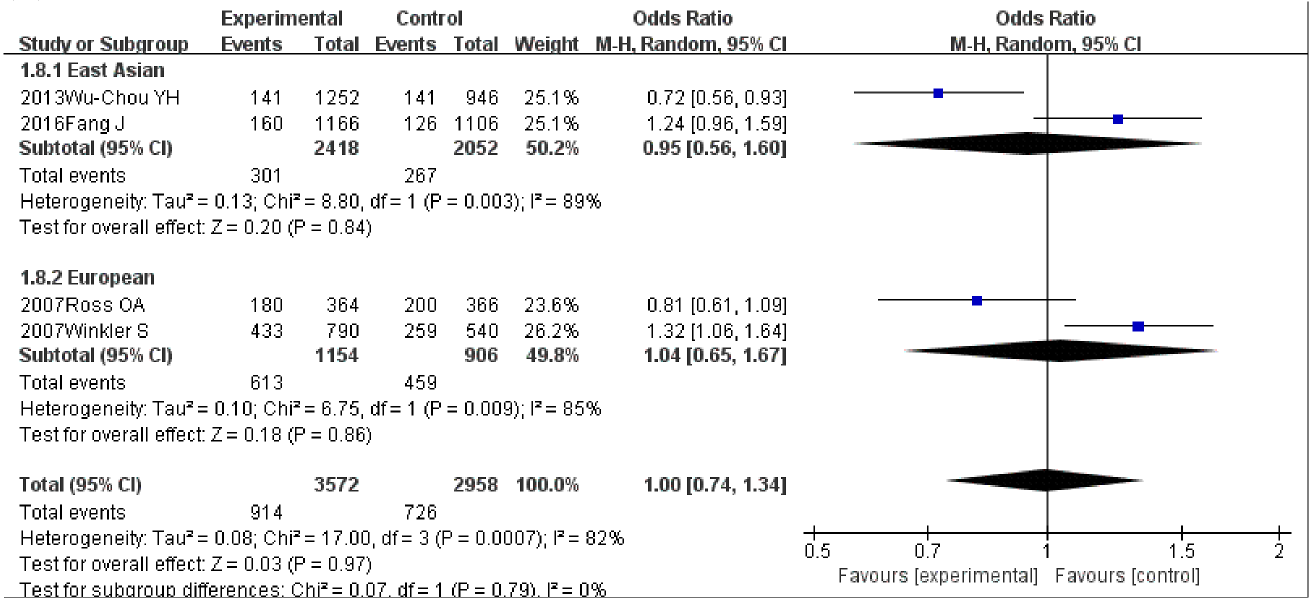

(I)

Study or Subgroup	Experimental Events	Experimental Total	Control Events	Control Total	Weight	Odds Ratio M-H, Random, 95% CI
**1.9.1 East Asian**						
2013Wu-Chou YH	133	1252	154	946	21.0%	0.61 [0.48, 0.78]
2016Fang J	78	1166	10	1106	12.8%	7.86 [4.05, 15.26]
**Subtotal (95% CI)**		**2418**		**2052**	**33.8%**	**2.15 [0.16, 28.27]**
Total events	211		164			
Heterogeneity: Tau² = 3.39; Chi² = 52.80, df = 1 (P < 0.00001); I² = 98%						
Test for overall effect: Z = 0.58 (P = 0.56)						
**1.9.2 European**						
2008Myhre R	252	466	243	472	20.9%	1.11 [0.86, 1.43]
2009Chung SJ	1132	2164	1122	2164	22.9%	1.02 [0.90, 1.15]
2010Mata IF	685	1358	627	1332	22.5%	1.14 [0.98, 1.33]
**Subtotal (95% CI)**		**3988**		**3968**	**66.2%**	**1.07 [0.98, 1.17]**
Total events	2069		1992			
Heterogeneity: Tau² = 0.00; Chi² = 1.49, df = 2 (P = 0.47); I² = 0%						
Test for overall effect: Z = 1.51 (P = 0.13)						
**Total (95% CI)**		**6406**		**6020**	**100.0%**	**1.24 [0.87, 1.76]**
Total events	2280		2156			
Heterogeneity: Tau² = 0.14; Chi² = 55.38, df = 4 (P < 0.00001); I² = 93%						
Test for overall effect: Z = 1.21 (P = 0.23)						
Test for subgroup differences: Chi² = 0.28, df = 1 (P = 0.60), I² = 0%						

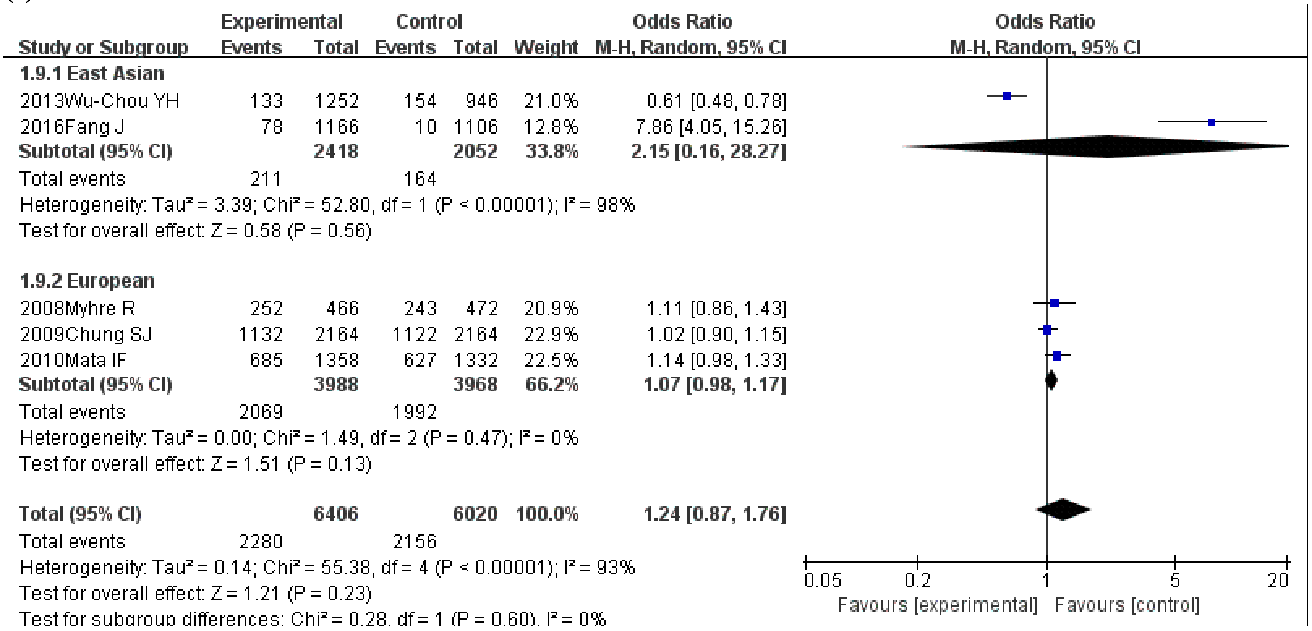

(J)

Study or Subgroup	Experimental Events	Experimental Total	Control Events	Control Total	Weight	Odds Ratio M-H, Random, 95% CI
**1.10.1 European**						
2007Ross OA	105	366	100	370	5.4%	1.09 [0.79, 1.50]
2007Winkler S	247	792	129	538	6.6%	1.44 [1.12, 1.84]
2008Myhre R	134	462	137	472	6.0%	1.00 [0.75, 1.33]
2011Elbaz A(1)	389	1704	388	1406	8.0%	0.78 [0.66, 0.91]
2011Elbaz A(2)	289	960	166	730	7.0%	1.46 [1.17, 1.83]
2011Elbaz A(3)	84	326	89	358	5.1%	1.05 [0.74, 1.48]
2011Elbaz A(4)	680	2172	356	1342	8.2%	1.26 [1.09, 1.47]
2011Elbaz A(5)	367	1204	333	1052	7.8%	0.95 [0.79, 1.13]
2011Elbaz A(6)	195	696	155	672	6.7%	1.30 [1.02, 1.66]
2011Elbaz A(7)	357	1124	77	286	5.9%	1.26 [0.95, 1.69]
2011Elbaz A(8)	280	896	222	800	7.2%	1.18 [0.96, 1.46]
2011Elbaz A(9)	234	692	223	866	7.1%	1.47 [1.18, 1.83]
2012Trotta L	557	1808	488	1782	8.3%	1.18 [1.02, 1.36]
**Subtotal (95% CI)**		**13202**		**10674**	**89.4%**	**1.16 [1.04, 1.31]**
Total events	3918		2863			
Heterogeneity: Tau² = 0.03; Chi² = 43.87, df = 12 (P < 0.0001); I² = 73%						
Test for overall effect: Z = 2.61 (P = 0.009)						
**1.10.2 Latino**						
2017Campêlo CL	60	208	38	196	3.7%	1.69 [1.06, 2.68]
**Subtotal (95% CI)**		**208**		**196**	**3.7%**	**1.69 [1.06, 2.68]**
Total events	60		38			
Heterogeneity: Not applicable						
Test for overall effect: Z = 2.21 (P = 0.03)						
**1.10.3 Mixed**						
2011Elbaz A(10)	246	756	168	722	6.9%	1.59 [1.26, 2.00]
**Subtotal (95% CI)**		**756**		**722**	**6.9%**	**1.59 [1.26, 2.00]**
Total events	246		168			
Heterogeneity: Not applicable						
Test for overall effect: Z = 3.95 (P < 0.0001)						
**Total (95% CI)**		**14166**		**11592**	**100.0%**	**1.21 [1.08, 1.35]**
Total events	4224		3069			
Heterogeneity: Tau² = 0.03; Chi² = 53.60, df = 14 (P < 0.00001); I² = 74%						
Test for overall effect: Z = 3.24 (P = 0.001)						
Test for subgroup differences: Chi² = 7.25, df = 2 (P = 0.03), I² = 72.4%						

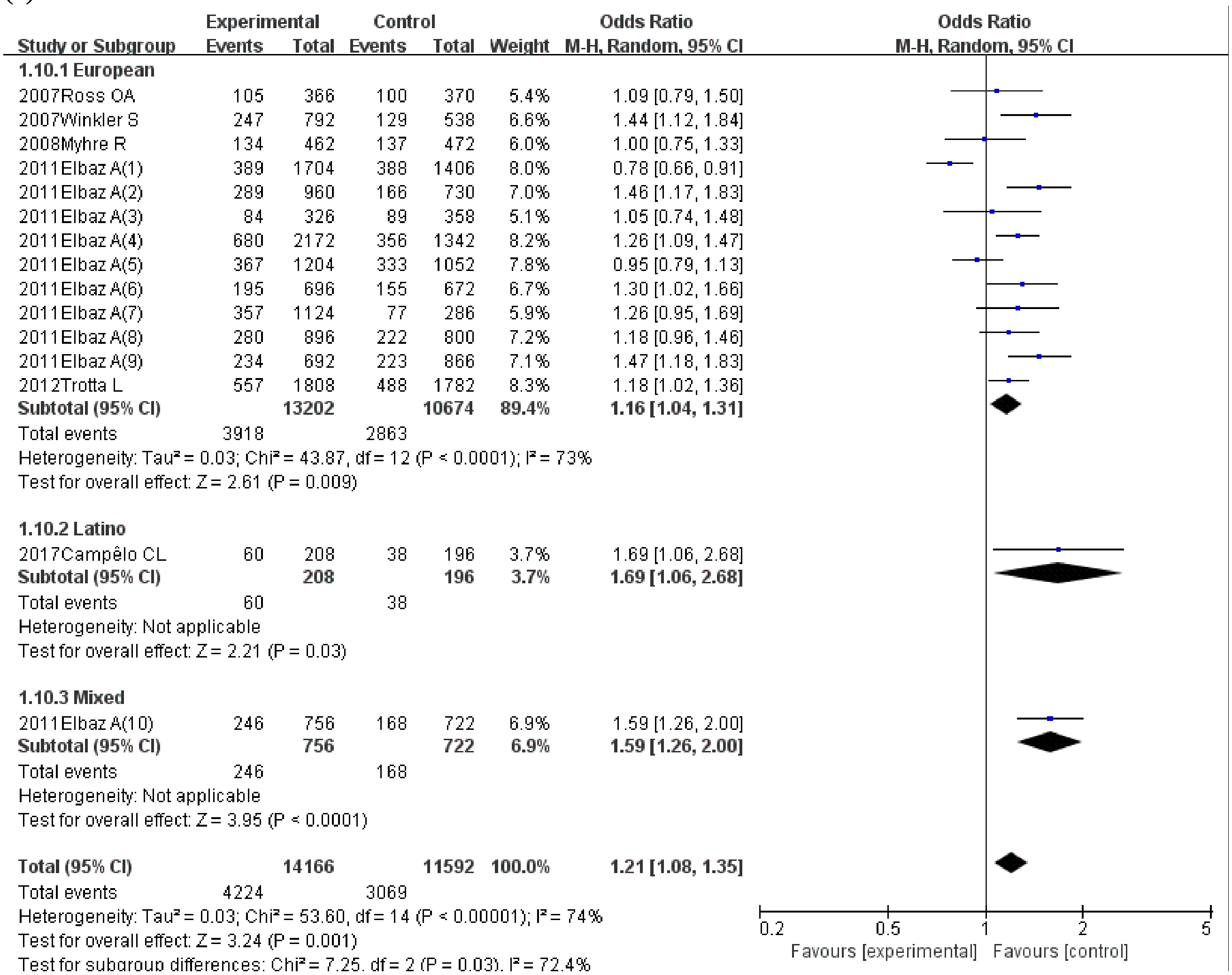

(K)

Study or Subgroup	Experimental Events	Experimental Total	Control Events	Control Total	Weight	Odds Ratio M-H, Fixed, 95% CI
**1.11.1 European**						
2007Parsian AJ	295	958	127	416	14.9%	1.01 [0.79, 1.30]
2007Ross OA	106	366	99	366	8.5%	1.10 [0.80, 1.52]
2007Winkler S	247	792	130	538	12.9%	1.42 [1.11, 1.82]
2008Myhre R	136	470	135	472	11.6%	1.02 [0.77, 1.35]
2009Chung SJ	667	2170	619	2170	52.0%	1.11 [0.98, 1.27]
**Subtotal (95% CI)**		**4756**		**3962**	**100.0%**	**1.13 [1.02, 1.24]**
Total events	1451		1110			
Heterogeneity: Chi² = 4.66, df = 4 (P = 0.32); I² = 14%						
Test for overall effect: Z = 2.46 (P = 0.01)						
**Total (95% CI)**		**4756**		**3962**	**100.0%**	**1.13 [1.02, 1.24]**
Total events	1451		1110			
Heterogeneity: Chi² = 4.66, df = 4 (P = 0.32); I² = 14%						
Test for overall effect: Z = 2.46 (P = 0.01)						
Test for subgroup differences: Not applicable						

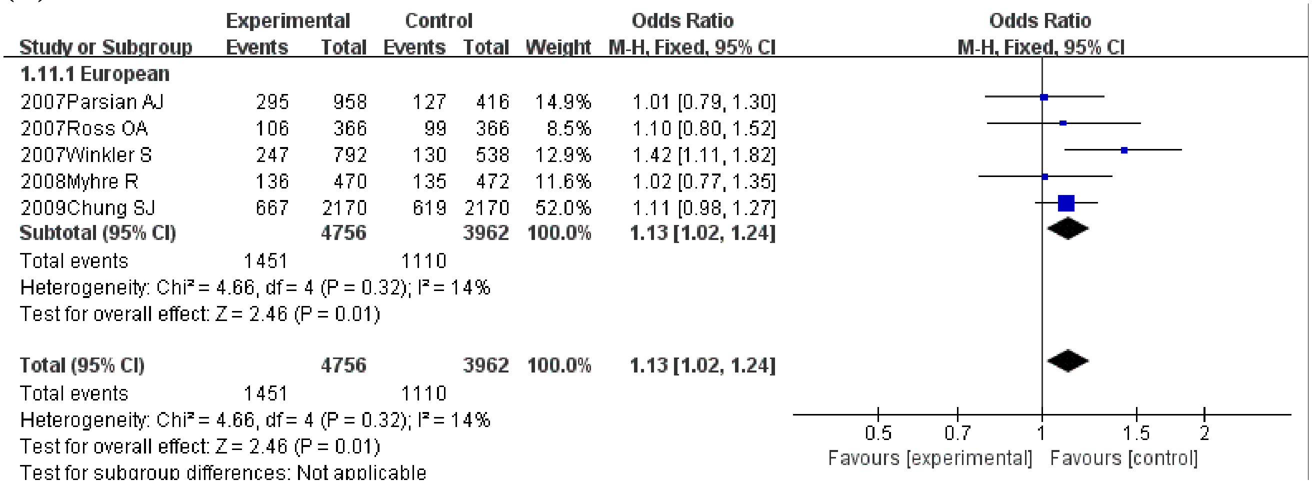

(L)

Study or Subgroup	Experimental Events	Experimental Total	Control Events	Control Total	Weight	Odds Ratio M-H, Fixed, 95% CI
**1.12.1 European**						
2007Ross OA	107	370	100	370	14.0%	1.10 [0.80, 1.51]
2007Winkler S	246	792	129	536	20.9%	1.42 [1.11, 1.82]
2008Myhre R	138	462	142	472	19.4%	0.99 [0.75, 1.31]
2010Mata IF	409	1356	328	1324	45.7%	1.31 [1.11, 1.56]
**Subtotal (95% CI)**		**2980**		**2702**	**100.0%**	**1.24 [1.11, 1.40]**
Total events	900		699			
Heterogeneity: Chi² = 4.61, df = 3 (P = 0.20); I² = 35%						
Test for overall effect: Z = 3.64 (P = 0.0003)						
**Total (95% CI)**		**2980**		**2702**	**100.0%**	**1.24 [1.11, 1.40]**
Total events	900		699			
Heterogeneity: Chi² = 4.61, df = 3 (P = 0.20); I² = 35%						
Test for overall effect: Z = 3.64 (P = 0.0003)						
Test for subgroup differences: Not applicable						

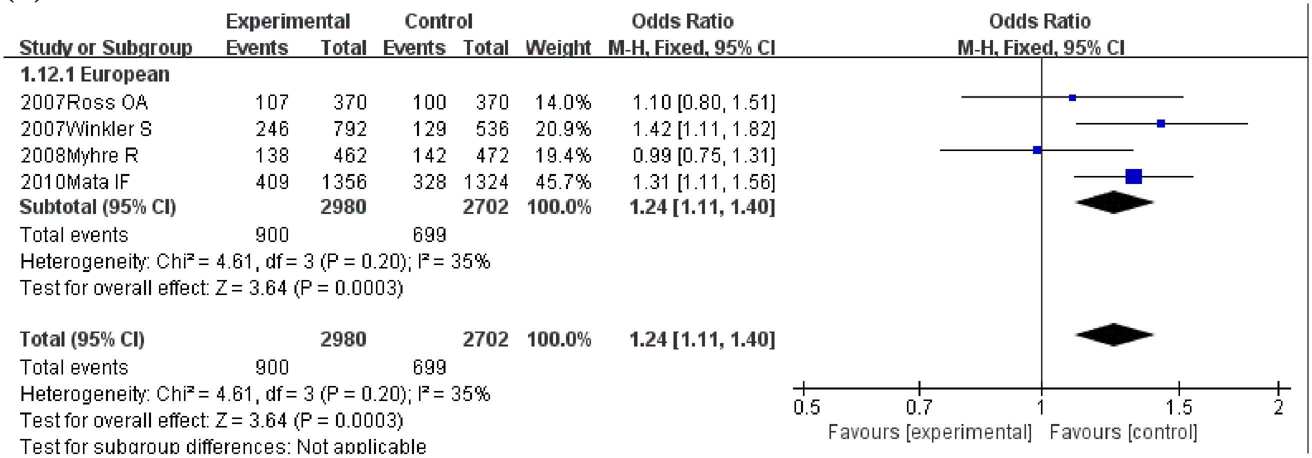

(M)

Study or Subgroup	Experimental Events	Experimental Total	Control Events	Control Total	Weight	Odds Ratio M-H, Fixed, 95% CI
**1.13.1 East Asian**						
2012Gan R	247	378	236	378	6.4%	1.13 [0.84, 1.53]
2012Miyake Y	319	458	460	714	8.5%	1.27 [0.99, 1.63]
2013Pan F	690	1030	556	900	15.3%	1.26 [1.04, 1.51]
2014Guo XY	1317	2022	874	1442	27.8%	1.21 [1.06, 1.40]
**Subtotal (95% CI)**		**3888**		**3434**	**58.1%**	**1.22 [1.11, 1.35]**
Total events	2573		2126			
Heterogeneity: Chi² = 0.41, df = 3 (P = 0.94); I² = 0%						
Test for overall effect: Z = 4.10 (P < 0.0001)						
**1.13.2 European**						
2009Chung SJ	1106	2168	1019	2168	39.1%	1.17 [1.04, 1.32]
**Subtotal (95% CI)**		**2168**		**2168**	**39.1%**	**1.17 [1.04, 1.32]**
Total events	1106		1019			
Heterogeneity: Not applicable						
Test for overall effect: Z = 2.64 (P = 0.008)						
**1.13.3 Latino**						
2017Campêlo CL	137	208	103	196	2.8%	1.74 [1.17, 2.60]
**Subtotal (95% CI)**		**208**		**196**	**2.8%**	**1.74 [1.17, 2.60]**
Total events	137		103			
Heterogeneity: Not applicable						
Test for overall effect: Z = 2.71 (P = 0.007)						
**Total (95% CI)**		**6264**		**5798**	**100.0%**	**1.22 [1.13, 1.31]**
Total events	3816		3248			
Heterogeneity: Chi² = 3.84, df = 5 (P = 0.57); I² = 0%						
Test for overall effect: Z = 5.27 (P < 0.00001)						
Test for subgroup differences: Chi² = 3.43, df = 2 (P = 0.18), I² = 41.7%						

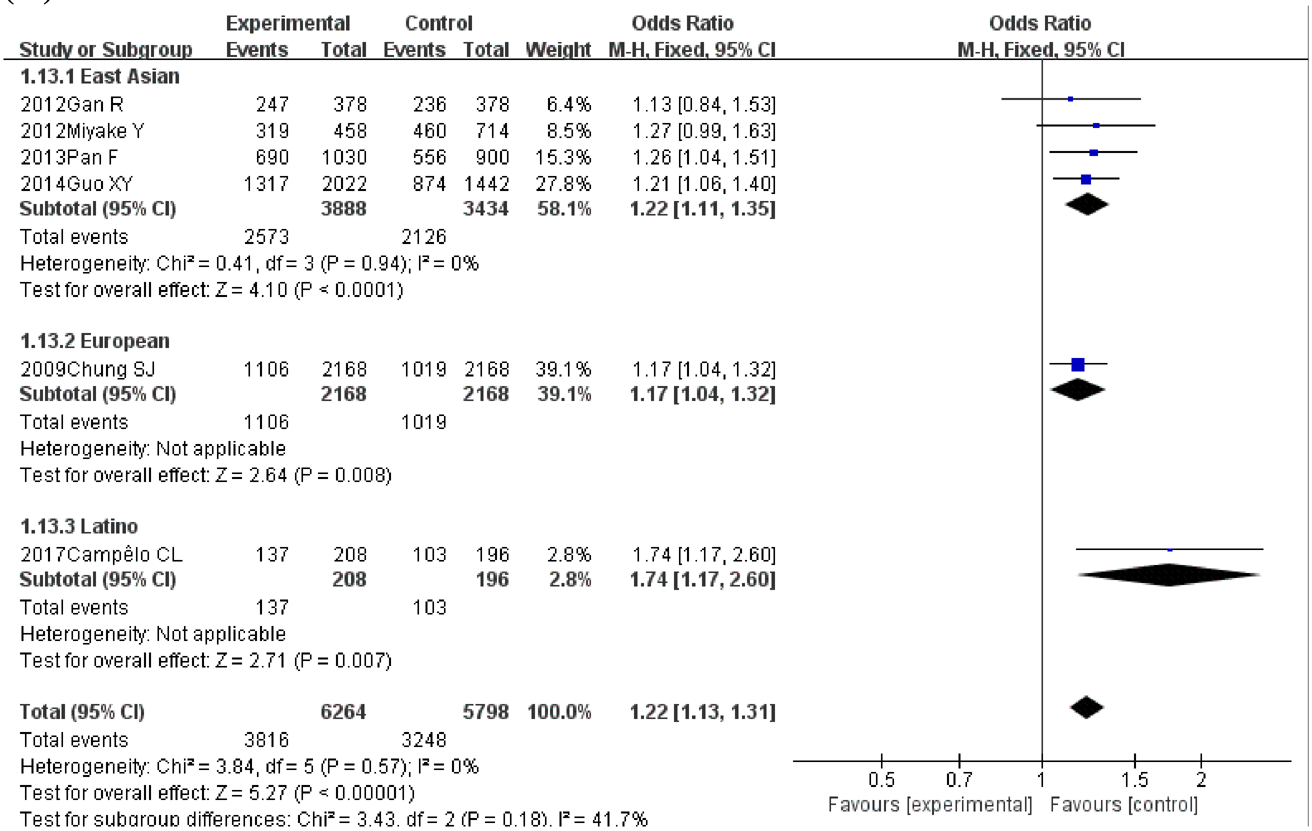

(N)

Study or Subgroup	Experimental Events	Experimental Total	Control Events	Control Total	Weight	Odds Ratio M-H, Random, 95% CI
**1.14.1 East Asian**						
2006Mizuta I	1181	1726	1074	1866	27.7%	1.60 [1.39, 1.83]
**Subtotal (95% CI)**		**1726**		**1866**	**27.7%**	**1.60 [1.39, 1.83]**
Total events	1181		1074			
Heterogeneity: Not applicable						
Test for overall effect: Z = 6.71 (P < 0.00001)						
**1.14.2 European**						
2008Westerlund M	253	576	212	600	18.3%	1.43 [1.13, 1.81]
2010Mata IF	618	1368	504	1342	25.9%	1.37 [1.18, 1.60]
2012Trotta L	799	1808	713	1782	28.1%	1.19 [1.04, 1.36]
**Subtotal (95% CI)**		**3752**		**3724**	**72.3%**	**1.30 [1.16, 1.45]**
Total events	1670		1429			
Heterogeneity: Tau² = 0.00; Chi² = 2.87, df = 2 (P = 0.24); I² = 30%						
Test for overall effect: Z = 4.47 (P < 0.00001)						
**Total (95% CI)**		**5478**		**5590**	**100.0%**	**1.38 [1.20, 1.59]**
Total events	2851		2503			
Heterogeneity: Tau² = 0.01; Chi² = 9.46, df = 3 (P = 0.02); I² = 68%						
Test for overall effect: Z = 4.54 (P < 0.00001)						
Test for subgroup differences: Chi² = 5.23, df = 1 (P = 0.02), I² = 80.9%						

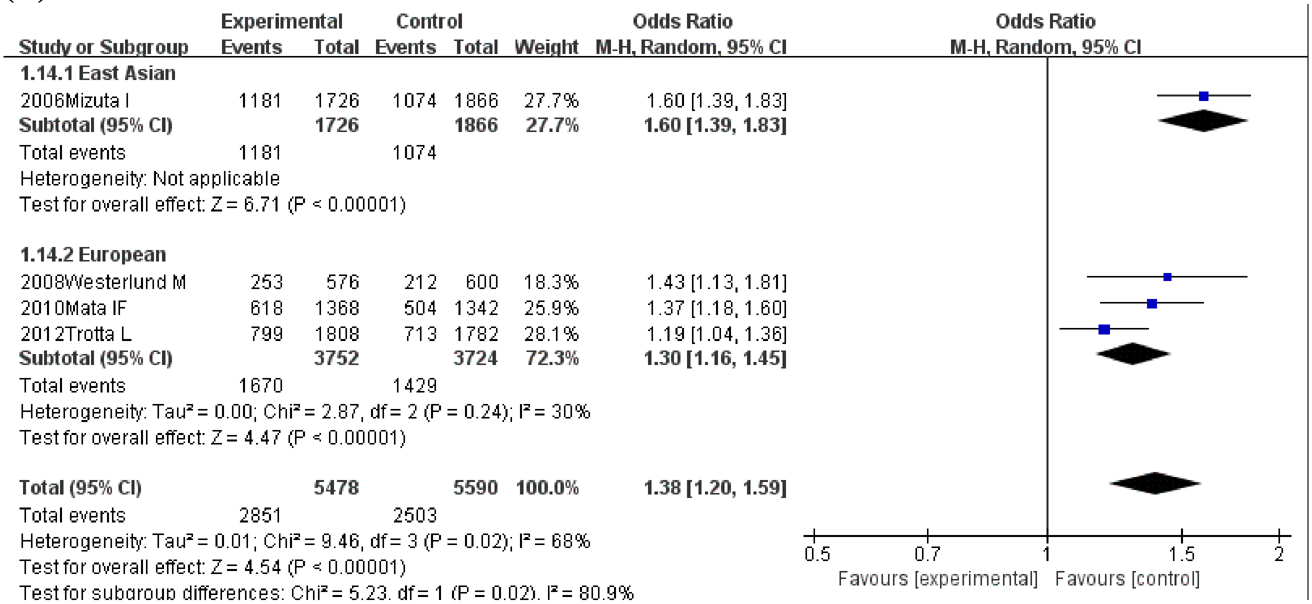

(O)

Study or Subgroup	Experimental Events	Experimental Total	Control Events	Control Total	Weight	Odds Ratio M-H, Random, 95% CI
2008Mizuta I	173	664	80	600	22.8%	2.29 [1.71, 3.07]
2010Yu L	297	458	427	714	25.8%	1.24 [0.97, 1.58]
2012Miyake Y	117	202	97	188	17.2%	1.29 [0.87, 1.93]
2013Liu B	1816	2740	2164	3792	34.2%	1.48 [1.34, 1.64]
**Total (95% CI)**		**4064**		**5294**	**100.0%**	**1.53 [1.22, 1.91]**
Total events	2403		2768			

Heterogeneity: Tau² = 0.04; Chi² = 11.01, df = 3 (P = 0.01); I² = 73%

Test for overall effect: Z = 3.64 (P = 0.0003)

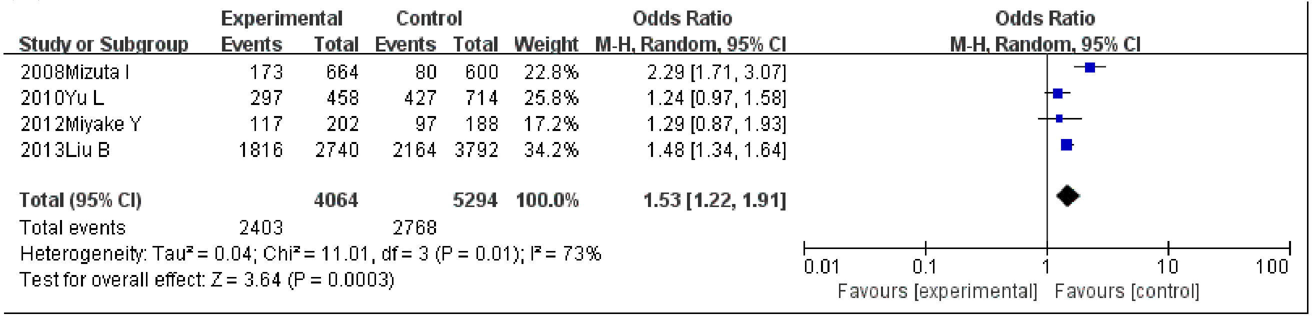

(P)

Study or Subgroup	Experimental Events	Experimental Total	Control Events	Control Total	Weight	Odds Ratio M-H, Fixed, 95% CI
**1.16.1 East Asian**						
2012Hu Y	139	220	147	272	2.0%	1.46 [1.01, 2.10]
2013Liu J	860	1414	722	1388	12.0%	1.43 [1.23, 1.66]
2013Wu-Chou YH	759	1266	486	888	9.7%	1.24 [1.04, 1.47]
2015Chen YP	1528	2538	887	1680	17.9%	1.35 [1.19, 1.53]
2015Guo JF	1214	2042	1069	2064	18.2%	1.36 [1.21, 1.54]
2015Wu GP	150	240	128	200	2.2%	0.94 [0.64, 1.38]
**Subtotal (95% CI)**		**7720**		**6492**	**62.1%**	**1.34 [1.26, 1.44]**
Total events	4650		3439			
Heterogeneity: Chi² = 5.09, df = 5 (P = 0.40); I² = 2%						
Test for overall effect: Z = 8.60 (P < 0.00001)						
**1.16.2 European**						
2007Ross OA	32	370	20	366	0.8%	1.64 [0.92, 2.92]
2007Winkler S	194	2176	85	1346	4.0%	1.45 [1.11, 1.89]
2011Elbaz A(1)	152	1838	102	1412	4.5%	1.16 [0.89, 1.50]
2011Elbaz A(2)	92	958	54	722	2.3%	1.31 [0.93, 1.87]
2011Elbaz A(3)	39	324	28	360	1.0%	1.62 [0.97, 2.70]
2011Elbaz A(4)	194	2176	85	1346	4.0%	1.45 [1.11, 1.89]
2011Elbaz A(5)	136	1184	82	1038	3.3%	1.51 [1.13, 2.02]
2011Elbaz A(6)	53	694	45	678	1.8%	1.16 [0.77, 1.76]
2011Elbaz A(7)	102	1122	20	286	1.2%	1.33 [0.81, 2.19]
2011Elbaz A(8)	79	902	62	810	2.5%	1.16 [0.82, 1.64]
2011Elbaz A(9)	69	716	55	888	1.9%	1.62 [1.12, 2.34]
**Subtotal (95% CI)**		**12460**		**9252**	**27.3%**	**1.37 [1.24, 1.52]**
Total events	1142		638			
Heterogeneity: Chi² = 5.55, df = 10 (P = 0.85); I² = 0%						
Test for overall effect: Z = 6.06 (P < 0.00001)						
**1.16.3 Latino**						
2017Campêlo CL	61	208	45	196	1.4%	1.39 [0.89, 2.18]
**Subtotal (95% CI)**		**208**		**196**	**1.4%**	**1.39 [0.89, 2.18]**
Total events	61		45			
Heterogeneity: Not applicable						
Test for overall effect: Z = 1.45 (P = 0.15)						
**1.16.4 Mixed**						
2011Elbaz A(10)	76	754	55	724	2.1%	1.36 [0.95, 1.96]
**Subtotal (95% CI)**		**754**		**724**	**2.1%**	**1.36 [0.95, 1.96]**
Total events	76		55			
Heterogeneity: Not applicable						
Test for overall effect: Z = 1.67 (P = 0.09)						
**1.16.5 West Asian**						
2016Shahmohammadibeni N	338	1040	250	1040	7.1%	1.52 [1.26, 1.84]
**Subtotal (95% CI)**		**1040**		**1040**	**7.1%**	**1.52 [1.26, 1.84]**
Total events	338		250			
Heterogeneity: Not applicable						
Test for overall effect: Z = 4.27 (P < 0.0001)						
**Total (95% CI)**		**22182**		**17704**	**100.0%**	**1.36 [1.29, 1.44]**
Total events	6267		4427			
Heterogeneity: Chi² = 12.11, df = 19 (P = 0.88); I² = 0%						
Test for overall effect: Z = 11.51 (P < 0.00001)						
Test for subgroup differences: Chi² = 1.47, df = 4 (P = 0.83), I² = 0%						

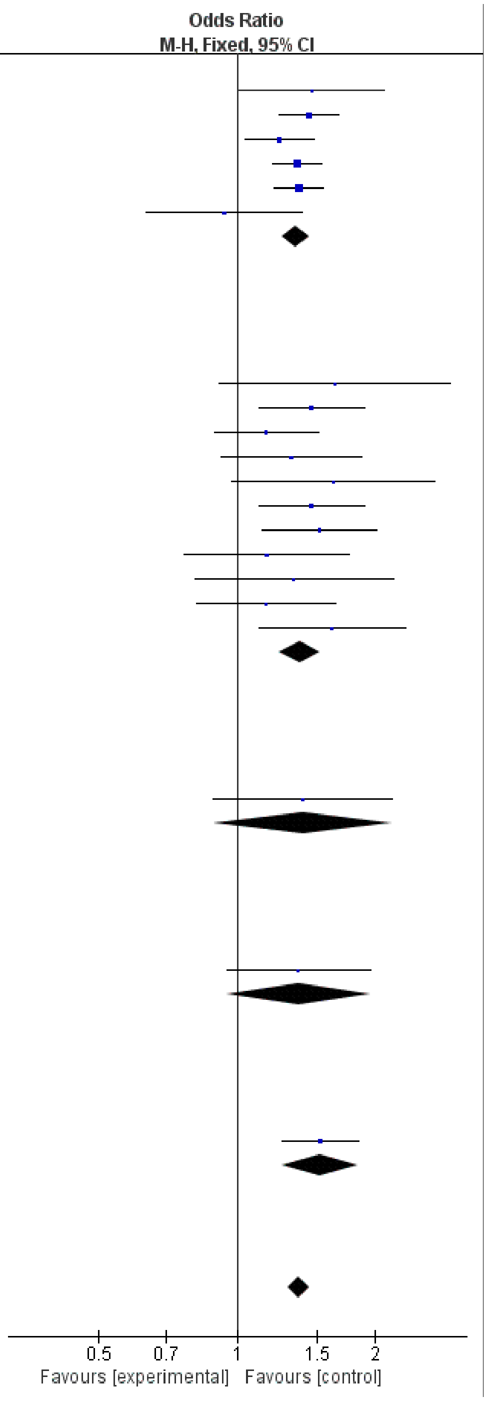

Supplementary Figure 1: Forest plots of the association between each *SNCA* variant (allele) included and PD risks in total and by ethnicity. (A)-(P) were individually responsive to the variants rs181489(T), rs356165(G), rs356186(A), rs356219(G), rs356220(T), rs356221(A), rs894278(G), rs2301134(A), rs2301135(G), rs2583988(T), rs2619363(T), rs2619364(G), rs2736990(G), rs2737029(G), rs7684318(C), rs11931074(T).
