# Supplementary material for: A Comprehensive Analysis of the Association Between SNCA Polymorphisms and the Risk of Parkinson's Disease
Source: Front Mol Neurosci. 2018 Oct 25;11:391. doi: 10.3389/fnmol.2018.00391 (PMC6209653; doi:10.3389/fnmol.2018.00391)
Supplement: Supplementary file 4 [file Image_2.PDF]

Supplementary Material

A comprehensive analysis of the association between *SNCA* polymorphisms with the risk of Parkinson's disease

Yuan Zhang[1 †], Li Shu[1 †], Qiying Sun[2,3,4], Hongxu Pan[1], Jifeng Guo[1,3,4,6,7,8], Beisha Tang[1, 2,3,4,5,6,7,8*]

[†] These authors have contributed equally to this work and are co-first authors.

[*] **Correspondence: Beisha Tang bstang7398@163.com**

(A)

Study or Subgroup	Experimental Events	Experimental Total	Control Events	Control Total	Weight	Odds Ratio M-H, Random, 95% CI
**1.1.1 European**						
2007Ross OA	109	182	91	184	5.5%	1.53 [1.01, 2.31]
2007Winkler S	215	395	135	269	7.8%	1.19 [0.87, 1.62]
2011Elbaz A(1)	501	901	365	685	11.5%	1.10 [0.90, 1.34]
2011Elbaz A(2)	280	464	161	333	8.6%	1.63 [1.22, 2.16]
2011Elbaz A(3)	80	160	85	176	5.2%	1.07 [0.70, 1.64]
2011Elbaz A(4)	587	1039	326	644	11.6%	1.27 [1.04, 1.54]
2011Elbaz A(5)	313	603	284	524	10.2%	0.91 [0.72, 1.15]
2011Elbaz A(6)	191	349	165	336	8.1%	1.25 [0.93, 1.69]
2011Elbaz A(7)	302	546	72	137	6.2%	1.12 [0.77, 1.63]
2011Elbaz A(8)	257	420	188	362	8.5%	1.46 [1.10, 1.94]
2011Elbaz A(9)	205	345	195	424	8.5%	1.72 [1.29, 2.29]
**Subtotal (95% CI)**		**5404**		**4074**	**91.7%**	**1.26 [1.12, 1.42]**
Total events	3040		2067			
Heterogeneity: $Tau^2 = 0.02$; $Chi^2 = 19.56$, df = 10 (P = 0.03); $I^2 = 49\%$						
Test for overall effect: Z = 3.80 (P = 0.0001)						
**1.1.2 Mixed**						
2011Elbaz A(10)	220	376	169	360	8.3%	1.59 [1.19, 2.13]
**Subtotal (95% CI)**		**376**		**360**	**8.3%**	**1.59 [1.19, 2.13]**
Total events	220		169			
Heterogeneity: Not applicable						
Test for overall effect: Z = 3.14 (P = 0.002)						
**Total (95% CI)**		**5780**		**4434**	**100.0%**	**1.29 [1.15, 1.45]**
Total events	3260		2236			
Heterogeneity: $Tau^2 = 0.02$; $Chi^2 = 22.11$, df = 11 (P = 0.02); $I^2 = 50\%$						
Test for overall effect: Z = 4.24 (P < 0.0001)						
Test for subgroup differences: $Chi^2 = 2.11$, df = 1 (P = 0.15), $I^2 = 52.5\%$						

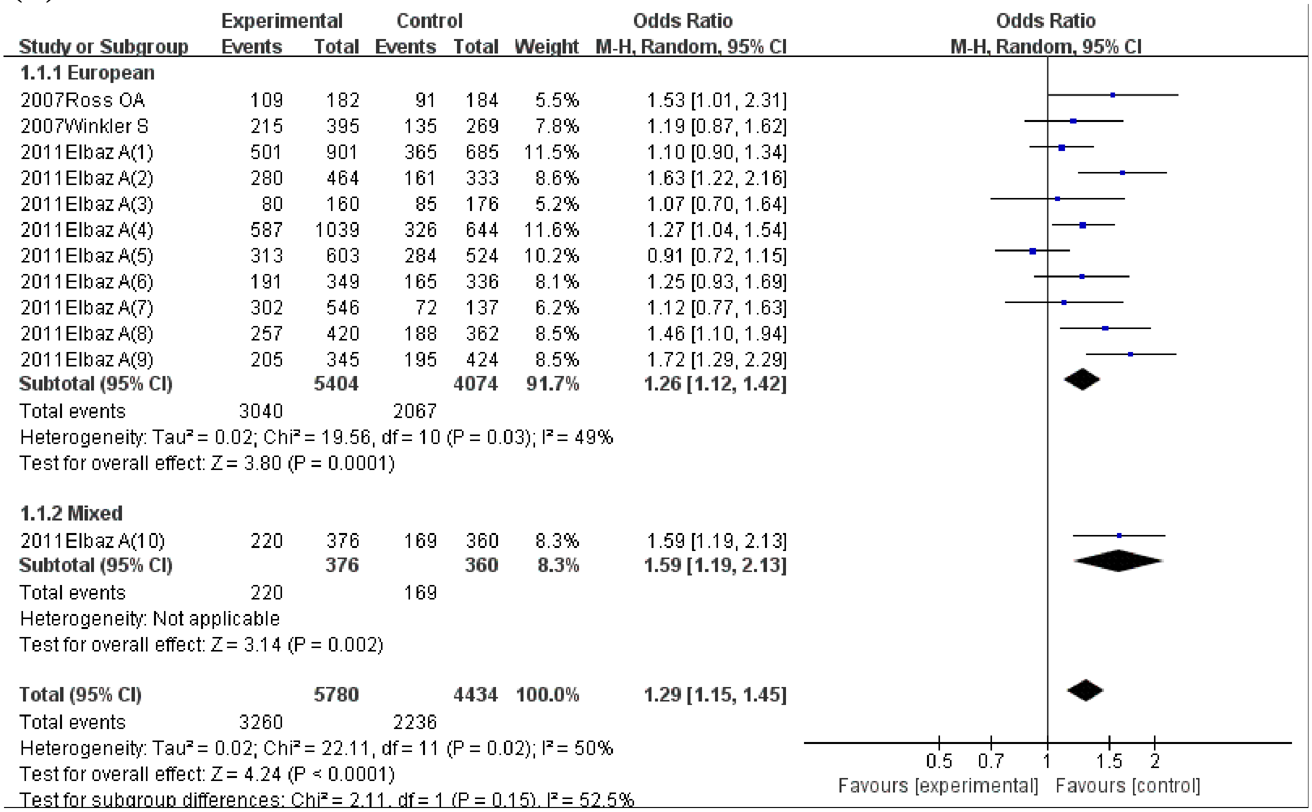

(B)

Study or Subgroup	Experimental Events	Experimental Total	Control Events	Control Total	Weight	Odds Ratio M-H, Random, 95% CI
**1.2.1 East Asian**						
2006Mizuta I	778	867	771	930	9.9%	1.80 [1.37, 2.38]
2010Hu FY	259	330	236	300	7.2%	0.99 [0.68, 1.45]
2013Liu B	85	116	79	95	3.2%	0.56 [0.28, 1.09]
2013Wu-Chou YH	526	626	396	473	8.5%	1.02 [0.74, 1.41]
2015Guo JF	854	1019	816	1030	11.6%	1.36 [1.08, 1.70]
2017Chen WJ	175	210	219	251	4.8%	0.73 [0.43, 1.23]
**Subtotal (95% CI)**		**3168**		**3079**	**45.1%**	**1.08 [0.81, 1.45]**
Total events	2677		2517			
Heterogeneity: Tau² = 0.09; Chi² = 19.68, df = 5 (P = 0.001); I² = 75%						
Test for overall effect: Z = 0.53 (P = 0.59)						
**1.2.2 European**						
2007Ross OA	127	180	107	181	6.1%	1.66 [1.07, 2.56]
2007Winkler S	249	391	151	269	8.7%	1.37 [1.00, 1.88]
2008Myhre R	143	210	131	217	6.8%	1.40 [0.94, 2.09]
2009Pankratz N	303	445	214	335	9.2%	1.21 [0.89, 1.63]
2012Cardo LF	685	1135	395	772	12.9%	1.45 [1.21, 1.75]
**Subtotal (95% CI)**		**2361**		**1774**	**43.8%**	**1.40 [1.24, 1.59]**
Total events	1507		998			
Heterogeneity: Tau² = 0.00; Chi² = 1.70, df = 4 (P = 0.79); I² = 0%						
Test for overall effect: Z = 5.22 (P < 0.00001)						
**1.2.3 Mixed**						
2014Lee PC	392	553	448	700	11.1%	1.37 [1.08, 1.74]
**Subtotal (95% CI)**		**553**		**700**	**11.1%**	**1.37 [1.08, 1.74]**
Total events	392		448			
Heterogeneity: Not applicable						
Test for overall effect: Z = 2.57 (P = 0.01)						
**Total (95% CI)**		**6082**		**5553**	**100.0%**	**1.27 [1.11, 1.46]**
Total events	4576		3963			
Heterogeneity: Tau² = 0.03; Chi² = 23.40, df = 11 (P = 0.02); I² = 53%						
Test for overall effect: Z = 3.55 (P = 0.0004)						
Test for subgroup differences: Chi² = 2.60, df = 2 (P = 0.27), I² = 23.2%						

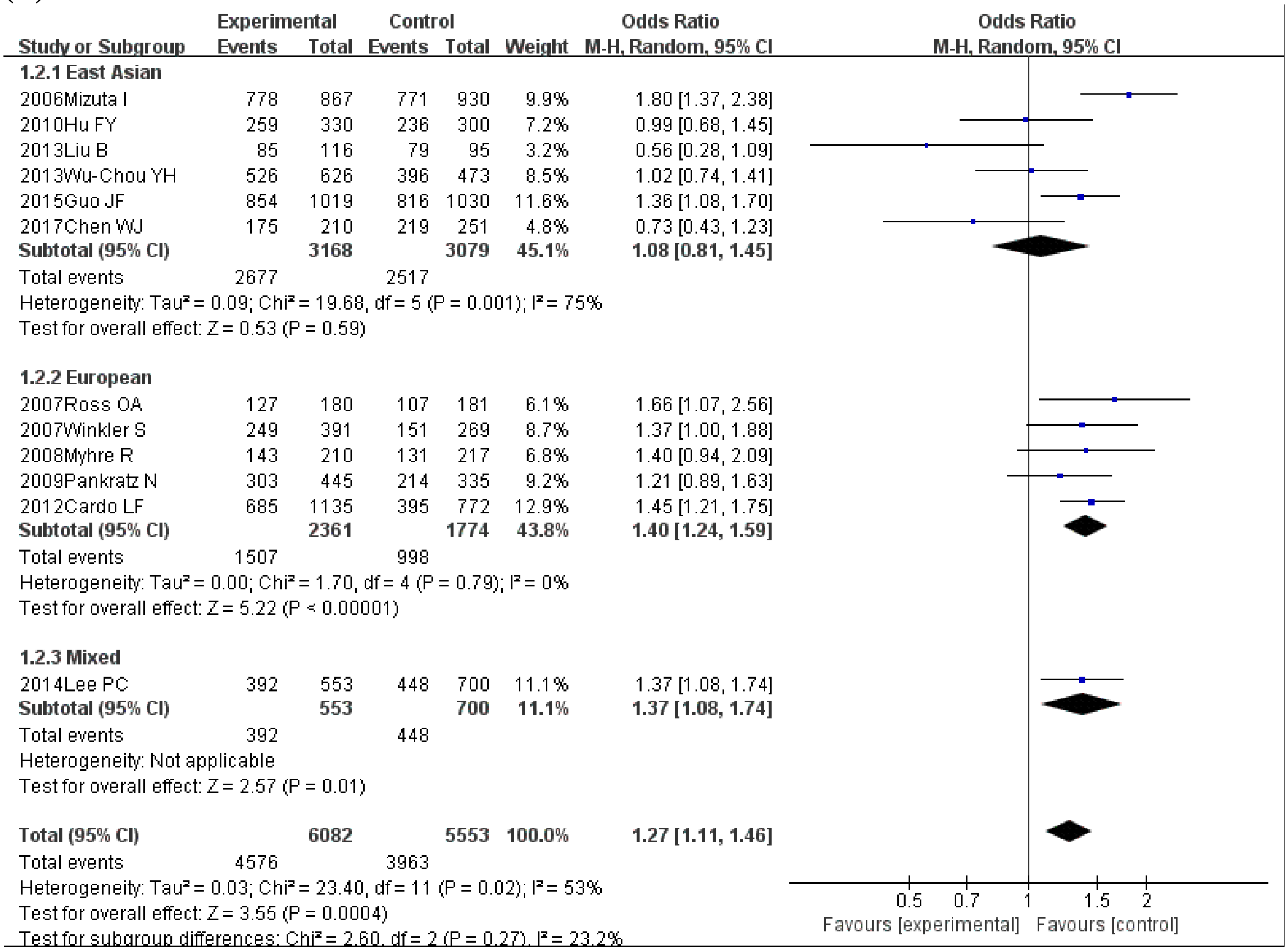

(C)

Study or Subgroup	Experimental Events	Experimental Total	Control Events	Control Total	Weight	Odds Ratio M-H, Fixed, 95% CI
**1.3.1 European**						
2007Ross OA	60	183	85	182	9.1%	0.56 [0.36, 0.85]
2007Winkler S	116	396	94	269	12.6%	0.77 [0.55, 1.07]
2009Chung SJ	277	904	343	891	38.0%	0.71 [0.58, 0.86]
2012Trotta L	302	1083	353	1083	40.4%	0.80 [0.67, 0.96]
**Subtotal (95% CI)**		**2566**		**2425**	**100.0%**	**0.74 [0.66, 0.83]**
Total events	755		875			
Heterogeneity: Chi² = 2.70, df = 3 (P = 0.44); I² = 0%						
Test for overall effect: Z = 4.99 (P < 0.00001)						
**Total (95% CI)**		**2566**		**2425**	**100.0%**	**0.74 [0.66, 0.83]**
Total events	755		875			
Heterogeneity: Chi² = 2.70, df = 3 (P = 0.44); I² = 0%						
Test for overall effect: Z = 4.99 (P < 0.00001)						
Test for subgroup differences: Not applicable						

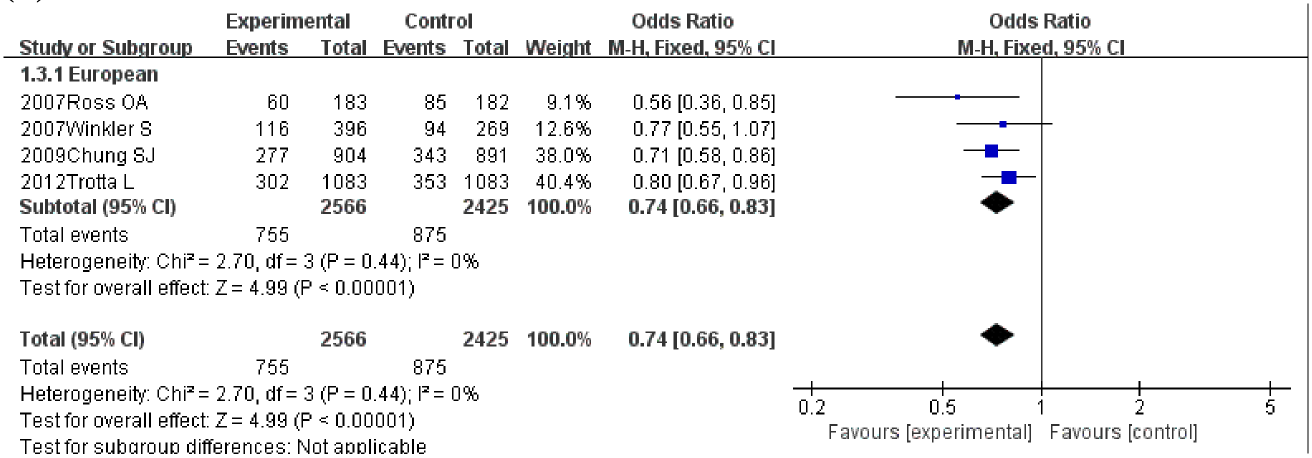

(D)

Study or Subgroup	Experimental Events	Experimental Total	Control Events	Control Total	Weight	Odds Ratio M-H, Fixed, 95% CI
**1.4.1 East Asian**						
2012Gan R	158	189	141	189	1.1%	1.74 [1.05, 2.88]
2012Li NN	613	685	460	569	2.4%	2.02 [1.46, 2.78]
2012Miyake Y	197	229	301	357	1.5%	1.15 [0.72, 1.83]
2012Pan F	349	403	244	315	1.7%	1.88 [1.27, 2.78]
2013Wu-Chou YH	526	633	360	444	3.3%	1.15 [0.84, 1.57]
**Subtotal (95% CI)**		**2139**		**1874**	**9.9%**	**1.55 [1.31, 1.83]**
Total events	1843		1506			
Heterogeneity: Chi² = 8.81, df = 4 (P = 0.07); I² = 55%						
Test for overall effect: Z = 5.05 (P < 0.00001)						
**1.4.2 European**						
2007Goris A	398	649	1254	2123	10.3%	1.10 [0.92, 1.32]
2008Myhre R	138	203	133	218	1.9%	1.36 [0.91, 2.03]
2008Westerlund M	156	245	149	250	2.4%	1.19 [0.83, 1.71]
2009Pankratz N	303	445	214	335	3.6%	1.21 [0.89, 1.63]
2010Mata IF	477	682	389	664	5.4%	1.64 [1.31, 2.06]
2011Botta-Orfila T	487	757	394	708	6.6%	1.44 [1.17, 1.77]
2011Elbaz A(1)	608	910	439	694	7.5%	1.17 [0.95, 1.44]
2011Elbaz A(2)	324	471	202	359	3.3%	1.71 [1.29, 2.28]
2011Elbaz A(3)	56	93	105	179	1.3%	1.07 [0.64, 1.78]
2011Elbaz A(4)	735	1077	399	671	7.1%	1.47 [1.20, 1.79]
2011Elbaz A(5)	403	601	346	526	5.5%	1.06 [0.83, 1.36]
2011Elbaz A(6)	214	341	202	338	3.4%	1.13 [0.83, 1.55]
2011Elbaz A(7)	379	562	90	141	2.1%	1.17 [0.80, 1.73]
2011Elbaz A(8)	298	450	252	404	4.1%	1.18 [0.89, 1.57]
2011Elbaz A(9)	256	353	301	428	3.4%	1.11 [0.81, 1.52]
2011Wider C	785	1020	748	1095	7.6%	1.55 [1.28, 1.88]
2012Trotta L	584	904	537	891	8.7%	1.20 [0.99, 1.46]
2013Emelyanov A	137	224	154	308	2.3%	1.57 [1.11, 2.23]
**Subtotal (95% CI)**		**9987**		**10332**	**86.6%**	**1.30 [1.22, 1.38]**
Total events	6738		6308			
Heterogeneity: Chi² = 25.46, df = 17 (P = 0.08); I² = 33%						
Test for overall effect: Z = 8.58 (P < 0.00001)						
**1.4.3 Latino**						
2017Campêlo CL	88	104	72	98	0.5%	1.99 [0.99, 3.98]
**Subtotal (95% CI)**		**104**		**98**	**0.5%**	**1.99 [0.99, 3.98]**
Total events	88		72			
Heterogeneity: Not applicable						
Test for overall effect: Z = 1.93 (P = 0.05)						
**1.4.4 Mixed**						
2011Elbaz A(10)	259	375	204	356	3.0%	1.66 [1.23, 2.25]
**Subtotal (95% CI)**		**375**		**356**	**3.0%**	**1.66 [1.23, 2.25]**
Total events	259		204			
Heterogeneity: Not applicable						
Test for overall effect: Z = 3.29 (P = 0.001)						
**Total (95% CI)**		**12605**		**12660**	**100.0%**	**1.34 [1.27, 1.41]**
Total events	8928		8090			
Heterogeneity: Chi² = 41.24, df = 24 (P = 0.02); I² = 42%						
Test for overall effect: Z = 10.33 (P < 0.00001)						
Test for subgroup differences: Chi² = 7.02, df = 3 (P = 0.07), I² = 57.3%						

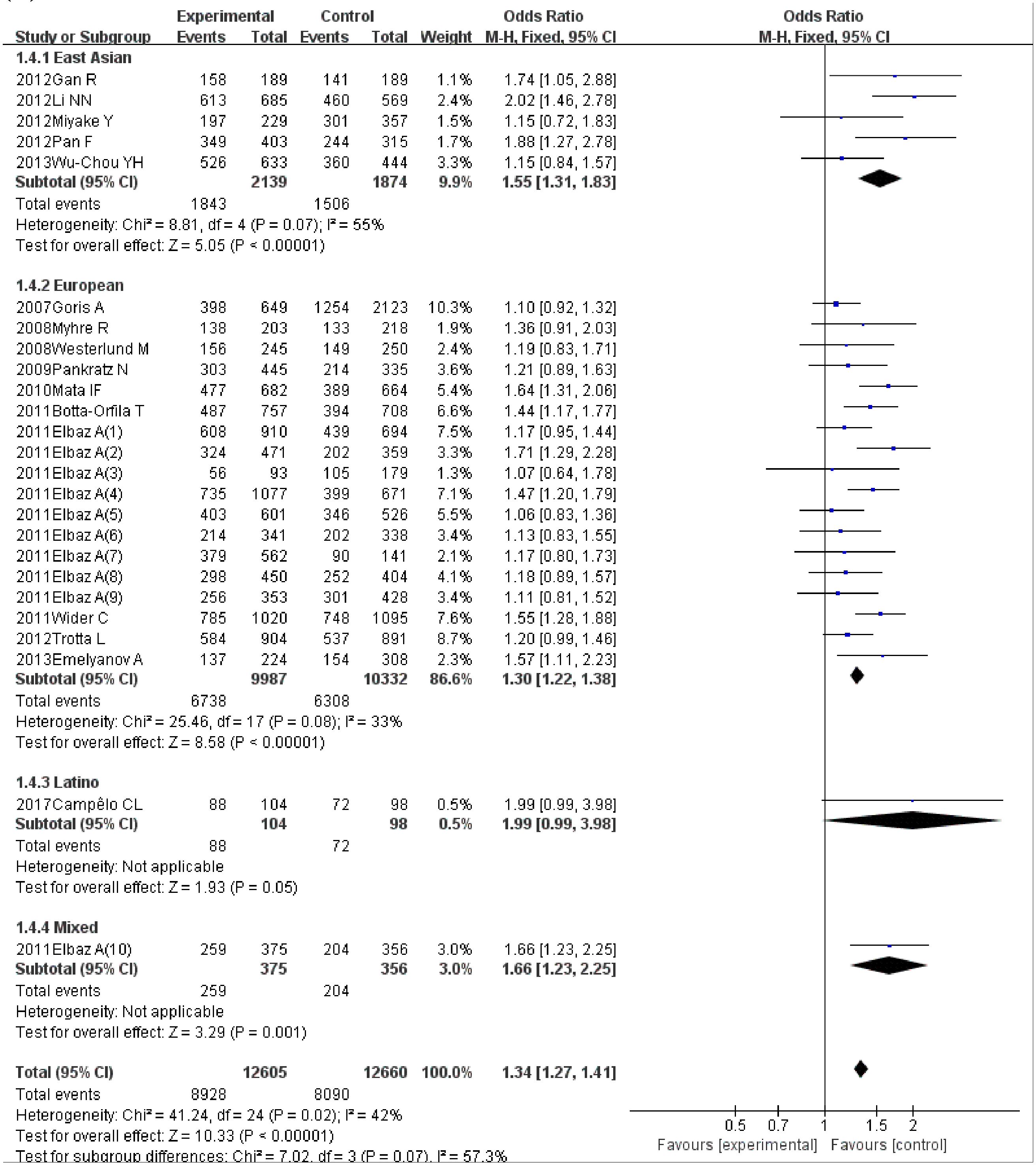

(E)

Study or Subgroup	Experimental Events	Experimental Total	Control Events	Control Total	Weight	Odds Ratio M-H, Fixed, 95% CI
**1.5.1 East Asian**						
2012Miyake Y	197	229	300	357	6.1%	1.17 [0.73, 1.87]
2014Guo XY	825	1011	576	721	23.2%	1.12 [0.88, 1.42]
**Subtotal (95% CI)**		**1240**		**1078**	**29.3%**	**1.13 [0.91, 1.40]**
Total events	1022		876			
Heterogeneity: Chi² = 0.03, df = 1 (P = 0.86); I² = 0%						
Test for overall effect: Z = 1.10 (P = 0.27)						
**1.5.2 European**						
2009Pankratz N	303	445	214	335	14.6%	1.21 [0.89, 1.63]
2012Trotta L	578	904	532	891	36.2%	1.20 [0.99, 1.45]
**Subtotal (95% CI)**		**1349**		**1226**	**50.8%**	**1.20 [1.02, 1.41]**
Total events	881		746			
Heterogeneity: Chi² = 0.00, df = 1 (P = 0.96); I² = 0%						
Test for overall effect: Z = 2.22 (P = 0.03)						
**1.5.3 West Asian**						
2016Shahmohammadibeni N	339	520	306	520	19.9%	1.31 [1.02, 1.68]
**Subtotal (95% CI)**		**520**		**520**	**19.9%**	**1.31 [1.02, 1.68]**
Total events	339		306			
Heterogeneity: Not applicable						
Test for overall effect: Z = 2.11 (P = 0.04)						
**Total (95% CI)**		**3109**		**2824**	**100.0%**	**1.20 [1.07, 1.35]**
Total events	2242		1928			
Heterogeneity: Chi² = 0.82, df = 4 (P = 0.94); I² = 0%						
Test for overall effect: Z = 3.13 (P = 0.002)						
Test for subgroup differences: Chi² = 0.79, df = 2 (P = 0.67), I² = 0%						

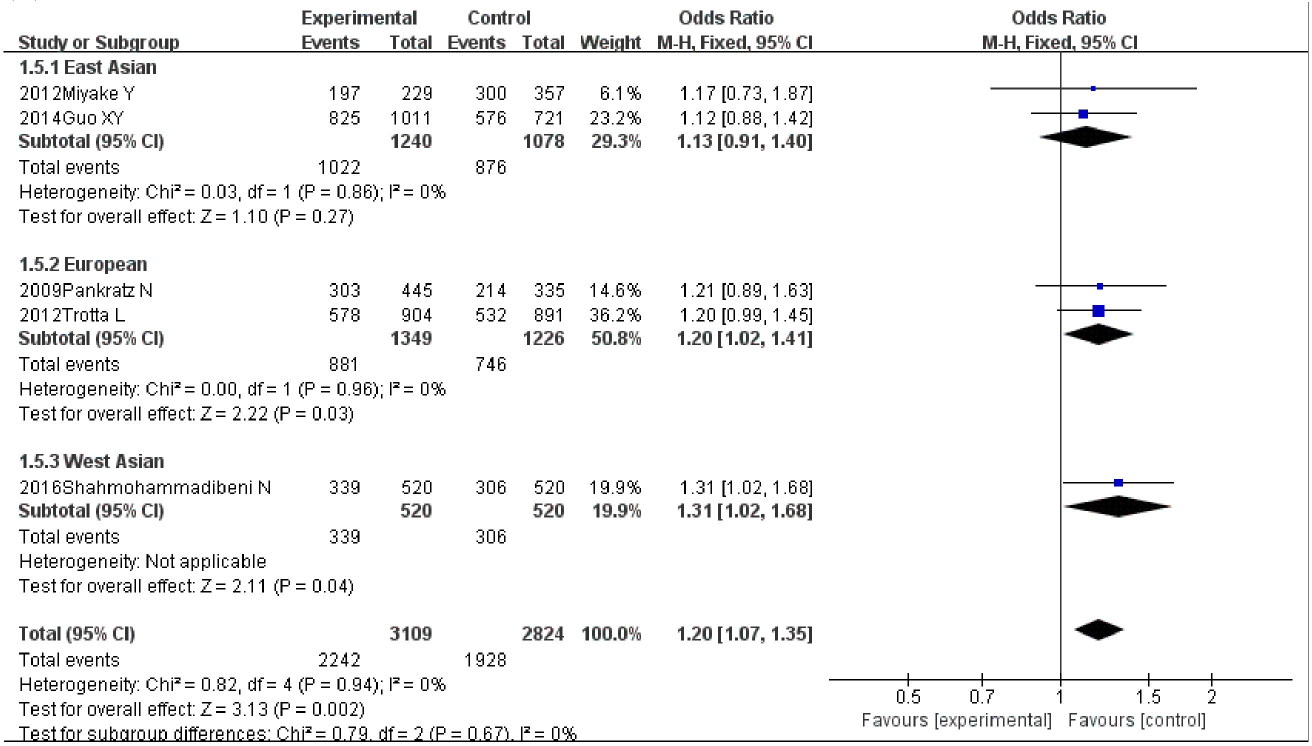

(F)

Study or Subgroup	Experimental Events	Experimental Total	Control Events	Control Total	Weight	Odds Ratio M-H, Fixed, 95% CI
**1.7.1 East Asian**						
2012Gan R	167	189	157	189	5.0%	1.55 [0.86, 2.78]
2012Miyake Y	208	229	311	357	6.0%	1.47 [0.85, 2.53]
2013Pan F	460	515	376	450	11.6%	1.65 [1.13, 2.39]
2014Guo XY	888	1011	615	721	23.7%	1.24 [0.94, 1.65]
**Subtotal (95% CI)**		**1944**		**1717**	**46.3%**	**1.41 [1.16, 1.71]**
Total events	1723		1459			
Heterogeneity: Chi² = 1.54, df = 3 (P = 0.67); I² = 0%						
Test for overall effect: Z = 3.43 (P = 0.0006)						
**1.7.2 European**						
2009Chung SJ	828	1084	792	1084	50.7%	1.19 [0.98, 1.45]
**Subtotal (95% CI)**		**1084**		**1084**	**50.7%**	**1.19 [0.98, 1.45]**
Total events	828		792			
Heterogeneity: Not applicable						
Test for overall effect: Z = 1.78 (P = 0.08)						
**1.7.3 Latino**						
2017Campêlo CL	90	104	79	98	3.0%	1.55 [0.73, 3.28]
**Subtotal (95% CI)**		**104**		**98**	**3.0%**	**1.55 [0.73, 3.28]**
Total events	90		79			
Heterogeneity: Not applicable						
Test for overall effect: Z = 1.13 (P = 0.26)						
**Total (95% CI)**		**3132**		**2899**	**100.0%**	**1.30 [1.14, 1.49]**
Total events	2641		2330			
Heterogeneity: Chi² = 3.11, df = 5 (P = 0.68); I² = 0%						
Test for overall effect: Z = 3.83 (P = 0.0001)						
Test for subgroup differences: Chi² = 1.59, df = 2 (P = 0.45), I² = 0%						

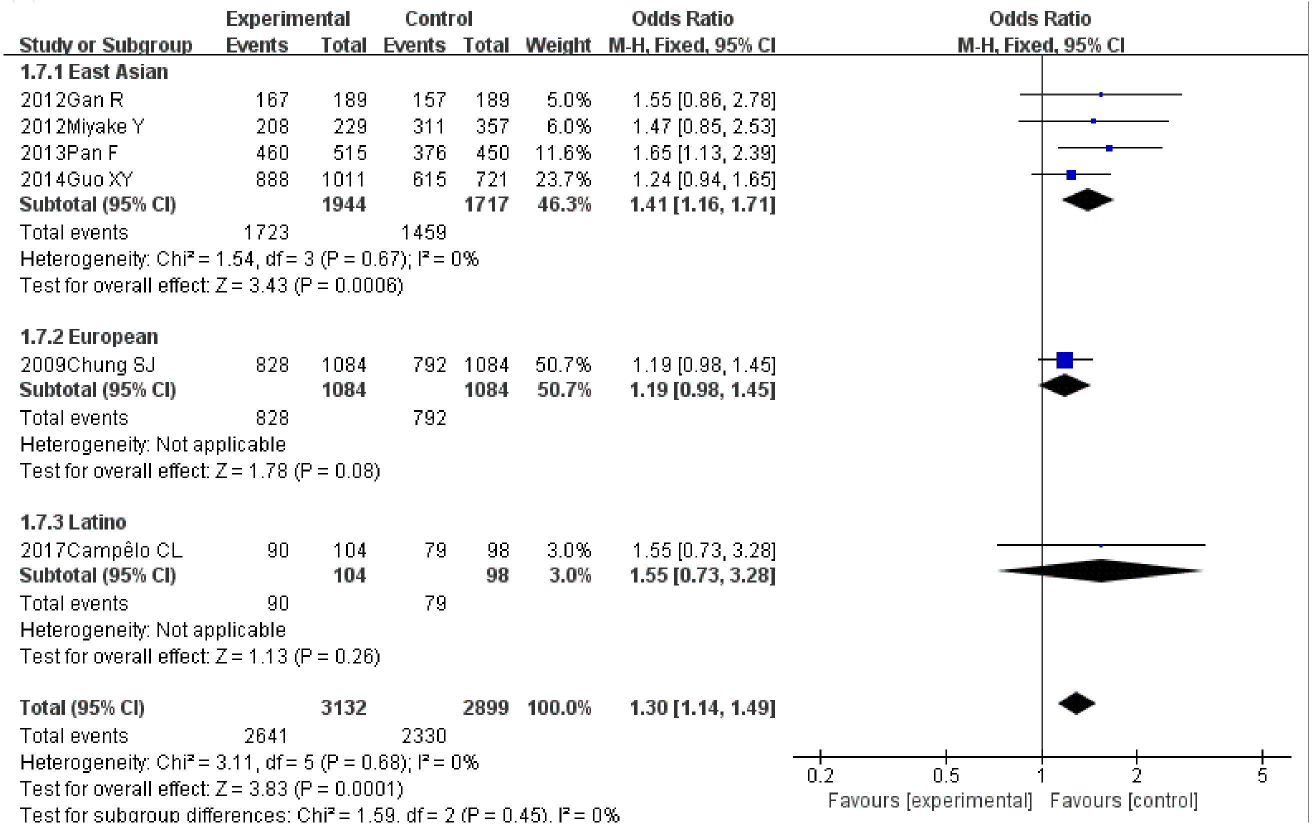

(G)

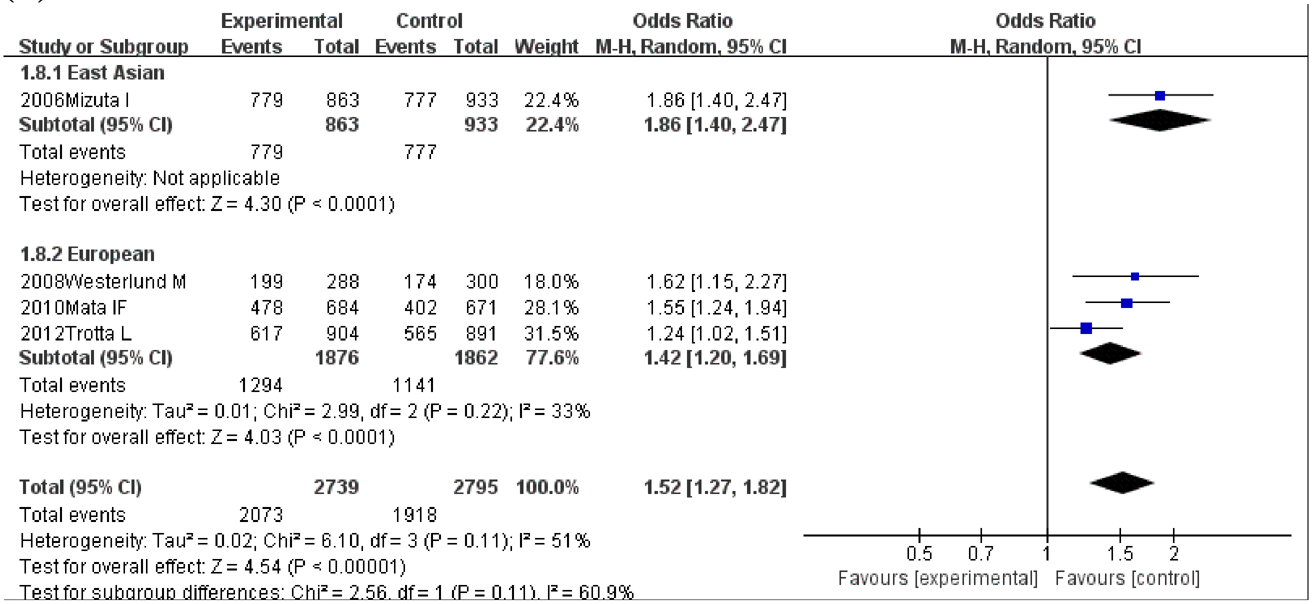
Experimental
Control
Odds Ratio
Odds Ratio
Study or Subgroup Events Total Events Total Weight M-H, Random, 95% CI
M-H, Random, 95% CI
1.8.1 East Asian
2006Mizuta I 779 863 777 933 22.4% 1.86 [1.40, 2.47]
Subtotal (95% CI) 863 933 22.4% 1.86 [1.40, 2.47]
Total events 779 777
Heterogeneity: Not applicable
Test for overall effect: Z = 4.30 (P < 0.0001)
1.8.2 European
2008Westerlund M 199 288 174 300 18.0% 1.62 [1.15, 2.27]
2010Mata IF 478 684 402 671 28.1% 1.55 [1.24, 1.94]
2012Trotta L 617 904 565 891 31.5% 1.24 [1.02, 1.51]
Subtotal (95% CI) 1876 1862 77.6% 1.42 [1.20, 1.69]
Total events 1294 1141
Heterogeneity: Tau² = 0.01; Chi² = 2.99, df = 2 (P = 0.22); I² = 33%
Test for overall effect: Z = 4.03 (P < 0.0001)
Total (95% CI) 2739 2795 100.0% 1.52 [1.27, 1.82]
Total events 2073 1918
Heterogeneity: Tau² = 0.02; Chi² = 6.10, df = 3 (P = 0.11); I² = 51%
Test for overall effect: Z = 4.54 (P < 0.00001)
Test for subgroup differences: Chi² = 2.56, df = 1 (P = 0.11), I² = 60.9%
0.5
0.7
1
1.5
2
Favours [experimental]
Favours [control]

(H)

Study or Subgroup	Experimental Events	Experimental Total	Control Events	Control Total	Weight	Odds Ratio M-H, Fixed, 95% CI
**1.9.1 East Asian**						
2012Hu Y	90	110	115	136	1.6%	0.82 [0.42, 1.61]
2013Liu J	592	707	532	694	7.5%	1.57 [1.20, 2.05]
2013Wu-Chou YH	530	633	357	444	5.9%	1.25 [0.91, 1.72]
2015Chen YP	1064	1269	662	840	11.1%	1.40 [1.12, 1.74]
2015Guo JF	873	1021	807	1032	10.0%	1.64 [1.31, 2.07]
2015Wu GP	101	120	85	100	1.3%	0.94 [0.45, 1.96]
**Subtotal (95% CI)**		**3860**		**3246**	**37.5%**	**1.43 [1.27, 1.62]**
Total events	3250		2558			
Heterogeneity: Chi² = 6.48, df = 5 (P = 0.26); I² = 23%						
Test for overall effect: Z = 5.83 (P < 0.00001)						
**1.9.2 European**						
2007Ross OA	31	185	18	183	1.3%	1.85 [0.99, 3.43]
2007Winkler S	179	1088	81	673	7.2%	1.44 [1.09, 1.91]
2011Elbaz A(1)	146	919	98	706	8.0%	1.17 [0.89, 1.55]
2011Elbaz A(2)	87	479	53	361	4.3%	1.29 [0.89, 1.87]
2011Elbaz A(3)	37	162	28	180	1.8%	1.61 [0.93, 2.77]
2011Elbaz A(4)	179	1088	81	673	7.2%	1.44 [1.09, 1.91]
2011Elbaz A(5)	131	592	80	519	5.7%	1.56 [1.15, 2.12]
2011Elbaz A(6)	51	347	42	339	3.1%	1.22 [0.79, 1.89]
2011Elbaz A(7)	98	561	19	143	2.2%	1.38 [0.81, 2.35]
2011Elbaz A(8)	77	451	60	405	4.5%	1.18 [0.82, 1.71]
2011Elbaz A(9)	67	358	52	444	3.3%	1.74 [1.17, 2.57]
**Subtotal (95% CI)**		**6230**		**4626**	**48.6%**	**1.39 [1.25, 1.55]**
Total events	1083		612			
Heterogeneity: Chi² = 5.65, df = 10 (P = 0.84); I² = 0%						
Test for overall effect: Z = 5.97 (P < 0.00001)						
**1.9.3 Latino**						
2017Campêlo CL	50	104	37	98	1.7%	1.53 [0.87, 2.68]
**Subtotal (95% CI)**		**104**		**98**	**1.7%**	**1.53 [0.87, 2.68]**
Total events	50		37			
Heterogeneity: Not applicable						
Test for overall effect: Z = 1.48 (P = 0.14)						
**1.9.4 Mixed**						
2011Elbaz A(10)	73	377	51	362	3.6%	1.46 [0.99, 2.17]
**Subtotal (95% CI)**		**377**		**362**	**3.6%**	**1.46 [0.99, 2.17]**
Total events	73		51			
Heterogeneity: Not applicable						
Test for overall effect: Z = 1.91 (P = 0.06)						
**1.9.5 West Asian**						
2016Shahmohammadibeni N	282	520	218	520	8.6%	1.64 [1.28, 2.10]
**Subtotal (95% CI)**		**520**		**520**	**8.6%**	**1.64 [1.28, 2.10]**
Total events	282		218			
Heterogeneity: Not applicable						
Test for overall effect: Z = 3.96 (P < 0.0001)						
**Total (95% CI)**		**11091**		**8852**	**100.0%**	**1.43 [1.33, 1.55]**
Total events	4738		3476			
Heterogeneity: Chi² = 13.65, df = 19 (P = 0.80); I² = 0%						
Test for overall effect: Z = 9.45 (P < 0.00001)						
Test for subgroup differences: Chi² = 1.51, df = 4 (P = 0.83), I² = 0%						

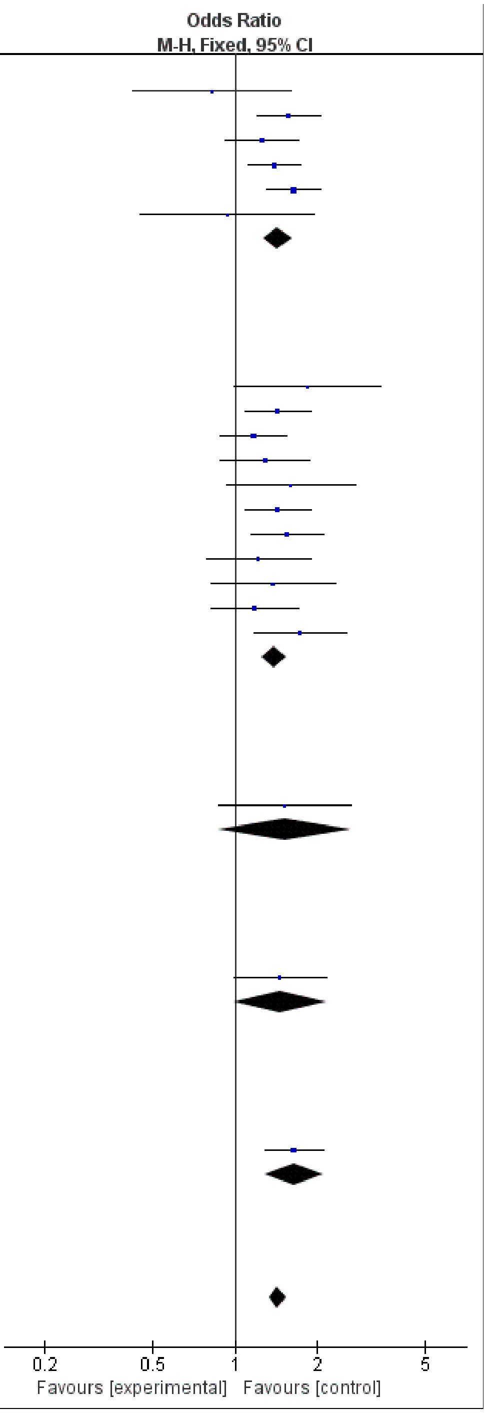

Supplementary Figure 2: Forest plots of the association between each *SNCA* variant (heterozygote) included and PD risks in total and by ethnicity. (A)-(H) were individually responsive to the variants rs181489(TT+TC), rs356165(GG+GA), rs356186(AA+AG), rs356219(GG+GA), rs356220(TT+TC), rs2736990(GG+GA), rs2737029(GG+GA), rs11931074(TT+TG).
