# Supplementary material for: A Comprehensive Analysis of the Association Between SNCA Polymorphisms and the Risk of Parkinson's Disease
Source: Front Mol Neurosci. 2018 Oct 25;11:391. doi: 10.3389/fnmol.2018.00391 (PMC6209653; doi:10.3389/fnmol.2018.00391)
Supplement: Supplementary file 5 [file Image_3.PDF]

Supplementary Material

A comprehensive analysis of the association between *SNCA* polymorphisms with the risk of Parkinson's disease

Yuan Zhang[1 †], Li Shu[1 †], Qiying Sun[2,3,4], Hongxu Pan[1], Jifeng Guo[1,3,4,6,7,8], Beisha Tang[1, 2,3,4,5,6,7,8*]

[†] These authors have contributed equally to this work and are co-first authors.

[*] **Correspondence: Beisha Tang bstang7398@163.com**

(A)

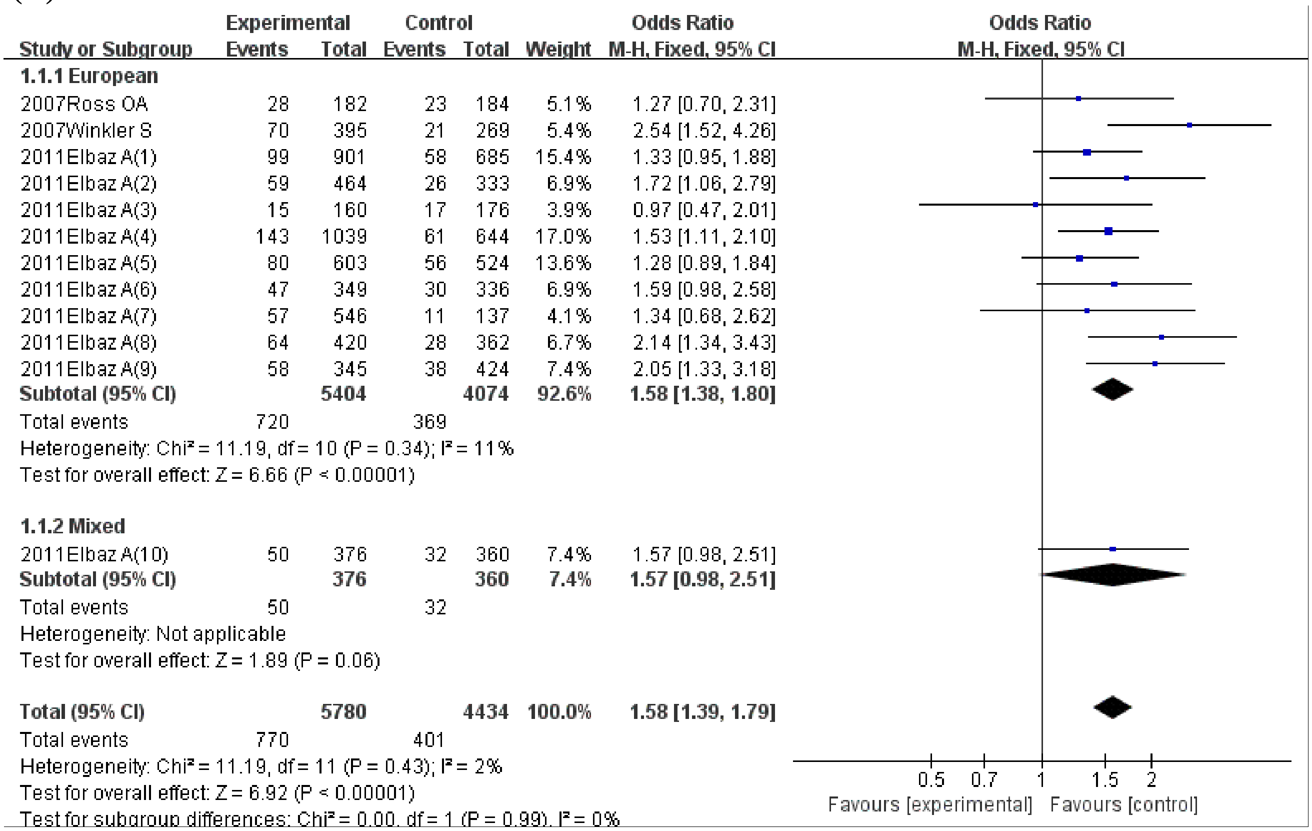

(B)

Study or Subgroup	Experimental Events	Experimental Total	Control Events	Control Total	Weight	Odds Ratio M-H, Fixed, 95% CI
**1.2.1 East Asian**						
2006Mizuta I	379	867	289	930	19.1%	1.72 [1.42, 2.09]
2010Hu FY	86	330	73	300	6.9%	1.10 [0.76, 1.57]
2013Liu B	36	116	31	95	2.9%	0.93 [0.52, 1.66]
2013Wu-Chou YH	204	626	121	473	11.3%	1.41 [1.08, 1.83]
2015Guo JF	330	1019	256	1030	20.9%	1.45 [1.19, 1.76]
2017Chen WJ	78	210	93	251	6.5%	1.00 [0.69, 1.47]
**Subtotal (95% CI)**		**3168**		**3079**	**67.5%**	**1.42 [1.27, 1.58]**
Total events	1113		863			
Heterogeneity: Chi² = 11.12, df = 5 (P = 0.05); I² = 55%						
Test for overall effect: Z = 6.34 (P < 0.00001)						
**1.2.2 European**						
2007Ross OA	41	180	30	181	2.8%	1.48 [0.88, 2.51]
2007Winkler S	78	391	29	269	3.3%	2.06 [1.30, 3.26]
2008Myhre R	47	210	29	217	2.7%	1.87 [1.12, 3.11]
2009Pankratz N	91	445	42	335	4.6%	1.79 [1.21, 2.67]
2012Cardo LF	171	1135	78	772	9.6%	1.58 [1.19, 2.10]
**Subtotal (95% CI)**		**2361**		**1774**	**23.1%**	**1.71 [1.43, 2.05]**
Total events	428		208			
Heterogeneity: Chi² = 1.40, df = 4 (P = 0.84); I² = 0%						
Test for overall effect: Z = 5.88 (P < 0.00001)						
**1.2.3 Mixed**						
2014Lee PC	107	553	109	700	9.4%	1.30 [0.97, 1.74]
**Subtotal (95% CI)**		**553**		**700**	**9.4%**	**1.30 [0.97, 1.74]**
Total events	107		109			
Heterogeneity: Not applicable						
Test for overall effect: Z = 1.75 (P = 0.08)						
**Total (95% CI)**		**6082**		**5553**	**100.0%**	**1.48 [1.35, 1.61]**
Total events	1648		1180			
Heterogeneity: Chi² = 16.36, df = 11 (P = 0.13); I² = 33%						
Test for overall effect: Z = 8.65 (P < 0.00001)						
Test for subgroup differences: Chi² = 3.90, df = 2 (P = 0.14), I² = 48.7%						

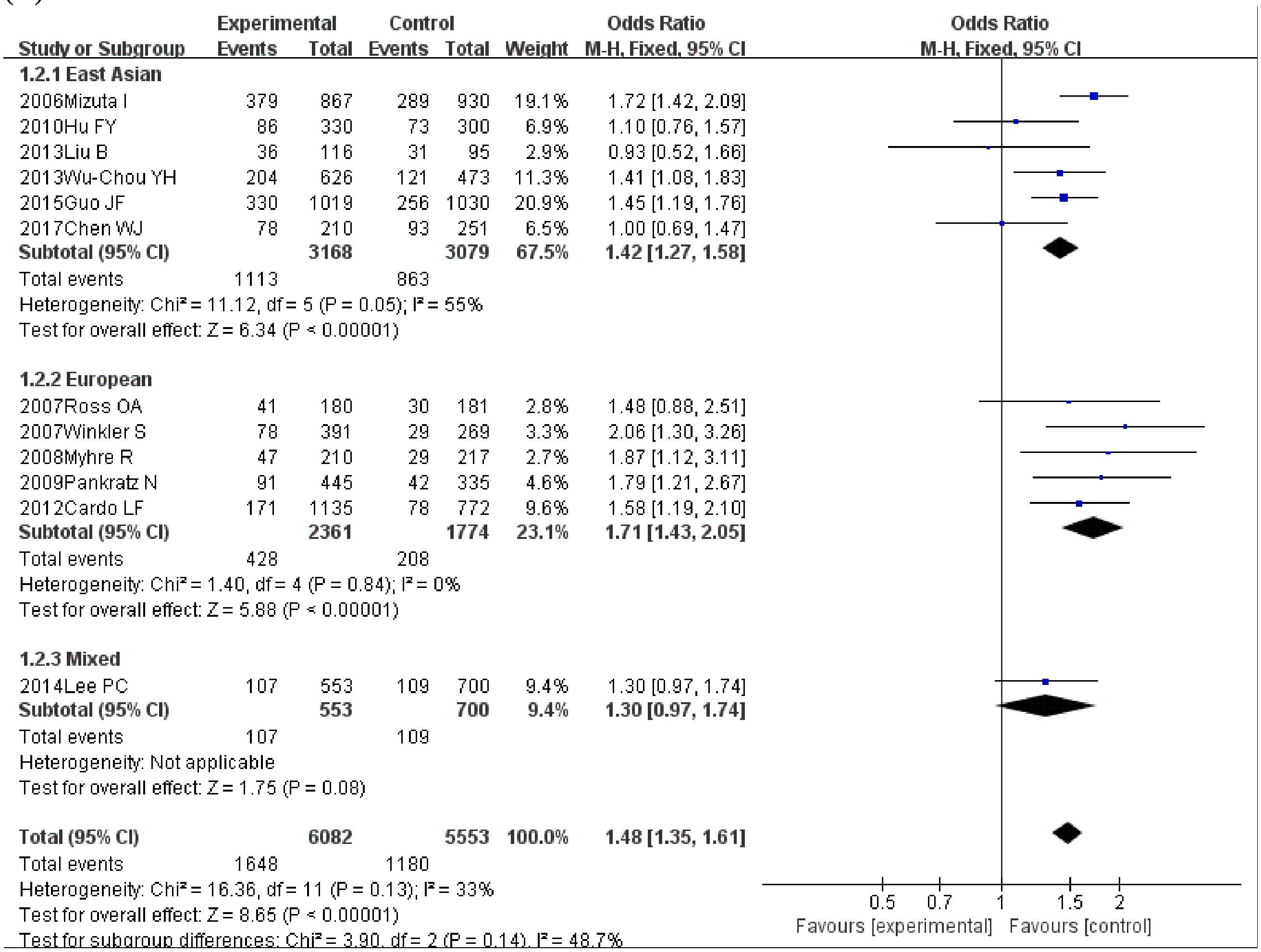

(C)

Study or Subgroup	Experimental Events	Experimental Total	Control Events	Control Total	Weight	Odds Ratio M-H, Fixed, 95% CI
**1.3.1 European**						
2007Ross OA	8	183	6	182	5.8%	1.34 [0.46, 3.94]
2007Winkler S	13	396	12	269	14.0%	0.73 [0.33, 1.62]
2009Chung SJ	25	904	45	891	44.8%	0.53 [0.32, 0.88]
2012Trotta L	35	1083	36	1083	35.4%	0.97 [0.61, 1.56]
**Subtotal (95% CI)**		**2566**		**2425**	**100.0%**	**0.76 [0.57, 1.03]**
Total events	81		99			
Heterogeneity: Chi² = 4.02, df = 3 (P = 0.26); I² = 25%						
Test for overall effect: Z = 1.77 (P = 0.08)						
**Total (95% CI)**		**2566**		**2425**	**100.0%**	**0.76 [0.57, 1.03]**
Total events	81		99			
Heterogeneity: Chi² = 4.02, df = 3 (P = 0.26); I² = 25%						
Test for overall effect: Z = 1.77 (P = 0.08)						
Test for subgroup differences: Not applicable						

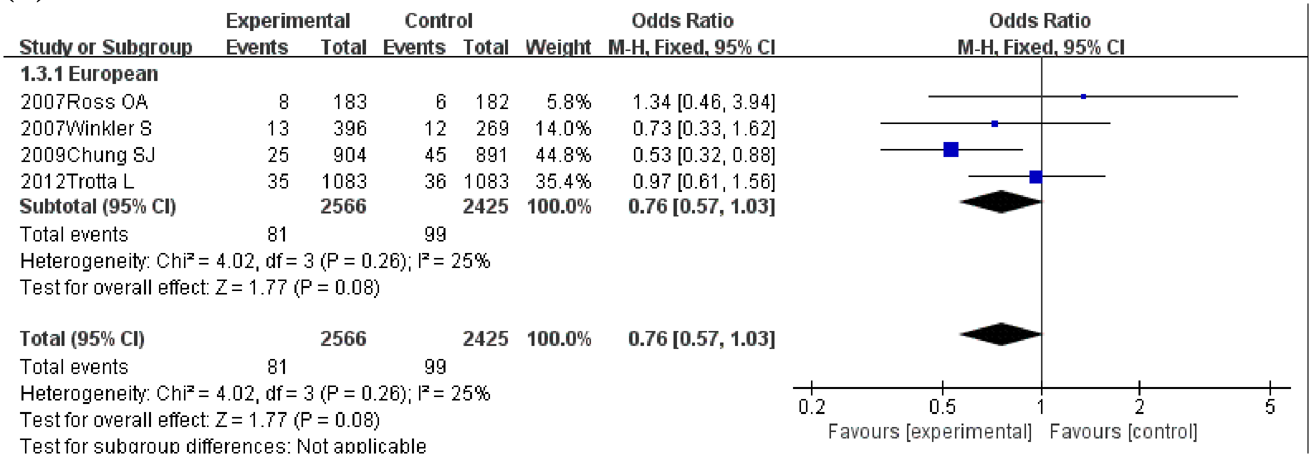

(D)

Study or Subgroup	Experimental Events	Experimental Total	Control Events	Control Total	Weight	Odds Ratio M-H, Fixed, 95% CI
**1.4.1 East Asian**						
2012Gan R	69	189	59	189	2.5%	1.27 [0.83, 1.94]
2012Li NN	312	685	148	569	5.9%	2.38 [1.87, 3.03]
2012Miyake Y	99	229	126	357	3.7%	1.40 [0.99, 1.96]
2012Pan F	170	403	102	315	4.4%	1.52 [1.12, 2.07]
2013Wu-Chou YH	226	633	139	444	7.0%	1.22 [0.94, 1.58]
**Subtotal (95% CI)**		**2139**		**1874**	**23.5%**	**1.60 [1.40, 1.82]**
Total events	876		574			
Heterogeneity: Chi² = 16.62, df = 4 (P = 0.002); I² = 76%						
Test for overall effect: Z = 6.98 (P < 0.00001)						
**1.4.2 European**						
2007Goris A	116	649	277	2123	7.1%	1.45 [1.14, 1.84]
2008Myhre R	44	203	27	218	1.4%	1.96 [1.16, 3.30]
2008Westerlund M	42	245	34	250	1.9%	1.31 [0.80, 2.15]
2009Pankratz N	91	445	42	335	2.5%	1.79 [1.21, 2.67]
2010Mata IF	137	682	78	664	4.2%	1.89 [1.40, 2.55]
2011Botta-Orfila T	132	757	71	708	4.0%	1.89 [1.39, 2.58]
2011Elbaz A(1)	150	910	102	694	6.4%	1.15 [0.87, 1.51]
2011Elbaz A(2)	88	471	46	359	2.8%	1.56 [1.06, 2.30]
2011Elbaz A(3)	17	93	25	179	0.9%	1.38 [0.70, 2.71]
2011Elbaz A(4)	217	1077	86	671	5.6%	1.72 [1.31, 2.25]
2011Elbaz A(5)	124	601	84	526	4.7%	1.37 [1.01, 1.86]
2011Elbaz A(6)	64	341	40	338	2.2%	1.72 [1.12, 2.64]
2011Elbaz A(7)	96	562	20	141	1.8%	1.25 [0.74, 2.10]
2011Elbaz A(8)	90	450	56	404	3.1%	1.55 [1.08, 2.24]
2011Elbaz A(9)	77	353	106	428	5.0%	0.85 [0.61, 1.18]
2011Wider C	307	1020	241	1095	10.8%	1.53 [1.25, 1.86]
2012Trotta L	162	904	132	891	7.3%	1.26 [0.98, 1.61]
2013Emelyanov A	30	224	28	308	1.4%	1.55 [0.90, 2.67]
**Subtotal (95% CI)**		**9987**		**10332**	**73.2%**	**1.47 [1.36, 1.59]**
Total events	1984		1495			
Heterogeneity: Chi² = 25.46, df = 17 (P = 0.08); I² = 33%						
Test for overall effect: Z = 9.90 (P < 0.00001)						
**1.4.3 Latino**						
2017Campêlo CL	38	104	21	98	0.9%	2.11 [1.13, 3.95]
**Subtotal (95% CI)**		**104**		**98**	**0.9%**	**2.11 [1.13, 3.95]**
Total events	38		21			
Heterogeneity: Not applicable						
Test for overall effect: Z = 2.34 (P = 0.02)						
**1.4.4 Mixed**						
2011Elbaz A(10)	80	375	44	356	2.4%	1.92 [1.29, 2.87]
**Subtotal (95% CI)**		**375**		**356**	**2.4%**	**1.92 [1.29, 2.87]**
Total events	80		44			
Heterogeneity: Not applicable						
Test for overall effect: Z = 3.20 (P = 0.001)						
**Total (95% CI)**		**12605**		**12660**	**100.0%**	**1.52 [1.42, 1.62]**
Total events	2978		2134			
Heterogeneity: Chi² = 45.77, df = 24 (P = 0.005); I² = 48%						
Test for overall effect: Z = 12.61 (P < 0.00001)						
Test for subgroup differences: Chi² = 3.69, df = 3 (P = 0.30), I² = 18.7%						

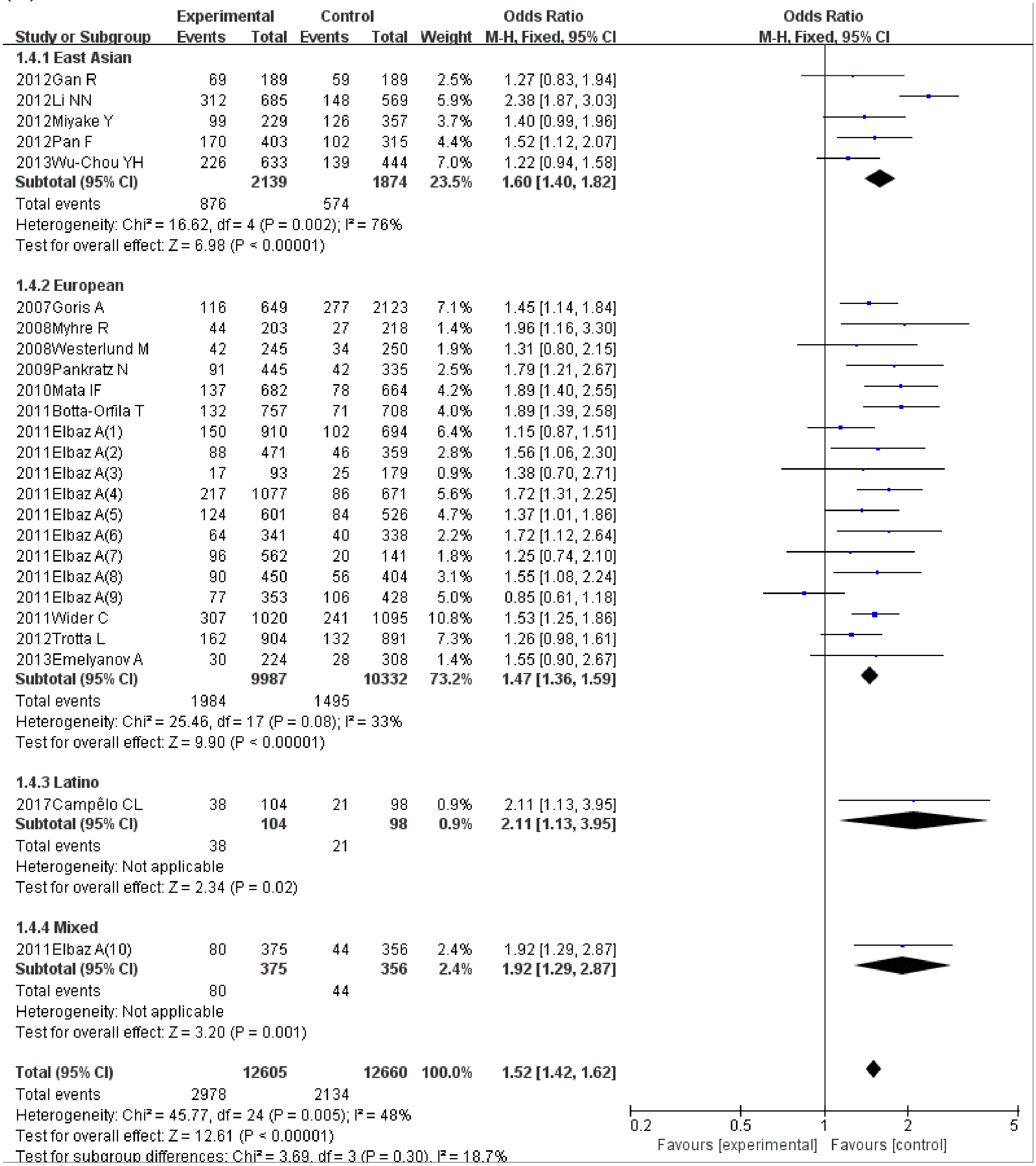

(E)

Study or Subgroup	Experimental Events	Experimental Total	Control Events	Control Total	Weight	Odds Ratio M-H, Fixed, 95% CI
**1.5.1 East Asian**						
2012Miyake Y	100	229	126	357	14.3%	1.42 [1.01, 2.00]
2014Guo XY	343	1011	193	721	38.4%	1.40 [1.14, 1.73]
**Subtotal (95% CI)**		**1240**		**1078**	**52.7%**	**1.41 [1.18, 1.68]**
Total events	443		319			
Heterogeneity: Chi² = 0.00, df = 1 (P = 0.95); I² = 0%						
Test for overall effect: Z = 3.76 (P = 0.0002)						
**1.5.2 European**						
2009Pankratz N	91	445	42	335	9.8%	1.79 [1.21, 2.67]
2012Trotta L	156	904	123	891	26.4%	1.30 [1.01, 1.68]
**Subtotal (95% CI)**		**1349**		**1226**	**36.3%**	**1.44 [1.16, 1.78]**
Total events	247		165			
Heterogeneity: Chi² = 1.76, df = 1 (P = 0.18); I² = 43%						
Test for overall effect: Z = 3.29 (P = 0.0010)						
**1.5.3 West Asian**						
2016Shahmohammadibeni N	93	520	52	520	11.0%	1.96 [1.36, 2.82]
**Subtotal (95% CI)**		**520**		**520**	**11.0%**	**1.96 [1.36, 2.82]**
Total events	93		52			
Heterogeneity: Not applicable						
Test for overall effect: Z = 3.63 (P = 0.0003)						
**Total (95% CI)**		**3109**		**2824**	**100.0%**	**1.48 [1.30, 1.68]**
Total events	783		536			
Heterogeneity: Chi² = 4.43, df = 4 (P = 0.35); I² = 10%						
Test for overall effect: Z = 5.98 (P < 0.00001)						
Test for subgroup differences: Chi² = 2.66, df = 2 (P = 0.26), I² = 24.8%						

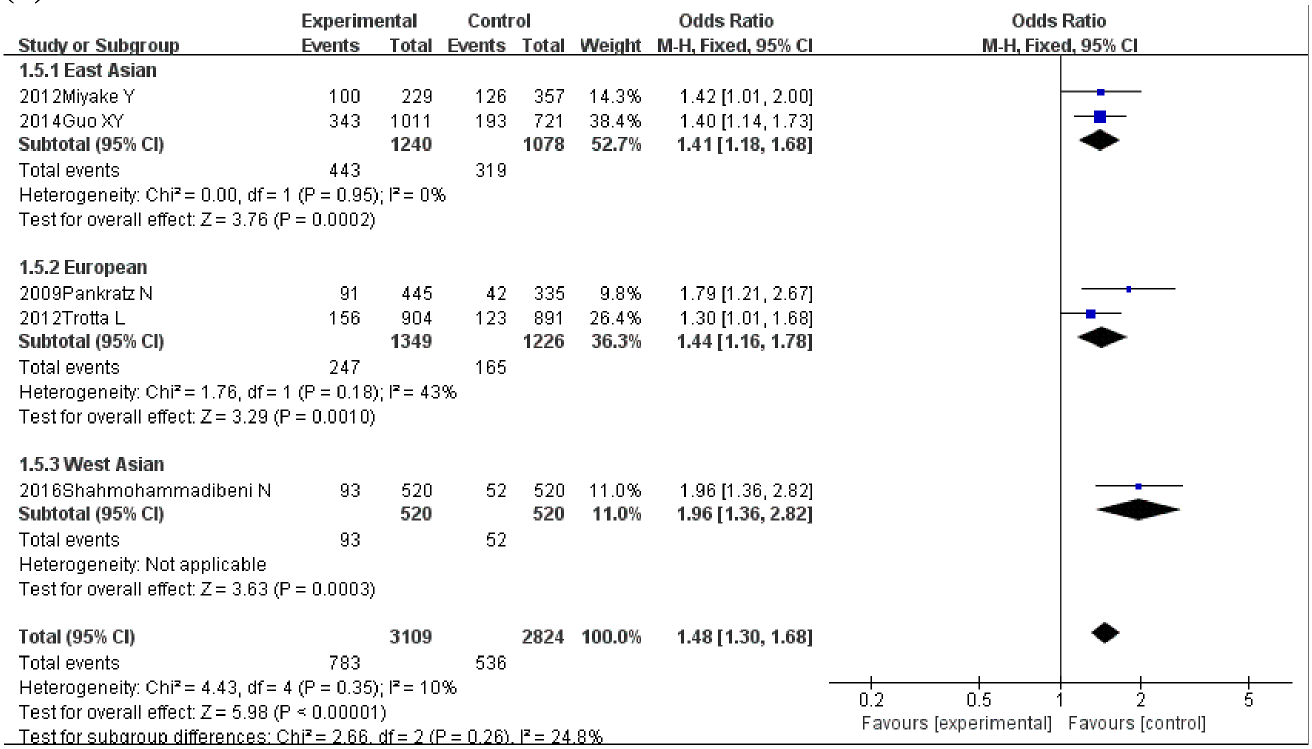

(F)

Study or Subgroup	Experimental Events	Experimental Total	Control Events	Control Total	Weight	Odds Ratio M-H, Fixed, 95% CI
**1.7.1 East Asian**						
2012Gan R	80	189	79	189	8.0%	1.02 [0.68, 1.54]
2012Miyake Y	111	229	149	357	10.6%	1.31 [0.94, 1.83]
2013Pan F	230	515	180	450	18.7%	1.21 [0.94, 1.56]
2014Guo XY	429	1011	259	721	30.6%	1.31 [1.08, 1.60]
**Subtotal (95% CI)**		**1944**		**1717**	**67.9%**	**1.25 [1.09, 1.43]**
Total events	850		667			
Heterogeneity: Chi² = 1.33, df = 3 (P = 0.72); I² = 0%						
Test for overall effect: Z = 3.29 (P = 0.001)						
**1.7.2 European**						
2009Chung SJ	278	1084	227	1084	29.7%	1.30 [1.07, 1.59]
**Subtotal (95% CI)**		**1084**		**1084**	**29.7%**	**1.30 [1.07, 1.59]**
Total events	278		227			
Heterogeneity: Not applicable						
Test for overall effect: Z = 2.59 (P = 0.010)						
**1.7.3 Latino**						
2017Campêlo CL	47	104	24	98	2.4%	2.54 [1.39, 4.64]
**Subtotal (95% CI)**		**104**		**98**	**2.4%**	**2.54 [1.39, 4.64]**
Total events	47		24			
Heterogeneity: Not applicable						
Test for overall effect: Z = 3.04 (P = 0.002)						
**Total (95% CI)**		**3132**		**2899**	**100.0%**	**1.30 [1.16, 1.45]**
Total events	1175		918			
Heterogeneity: Chi² = 6.43, df = 5 (P = 0.27); I² = 22%						
Test for overall effect: Z = 4.67 (P < 0.00001)						
Test for subgroup differences: Chi² = 5.10, df = 2 (P = 0.08), I² = 60.8%						

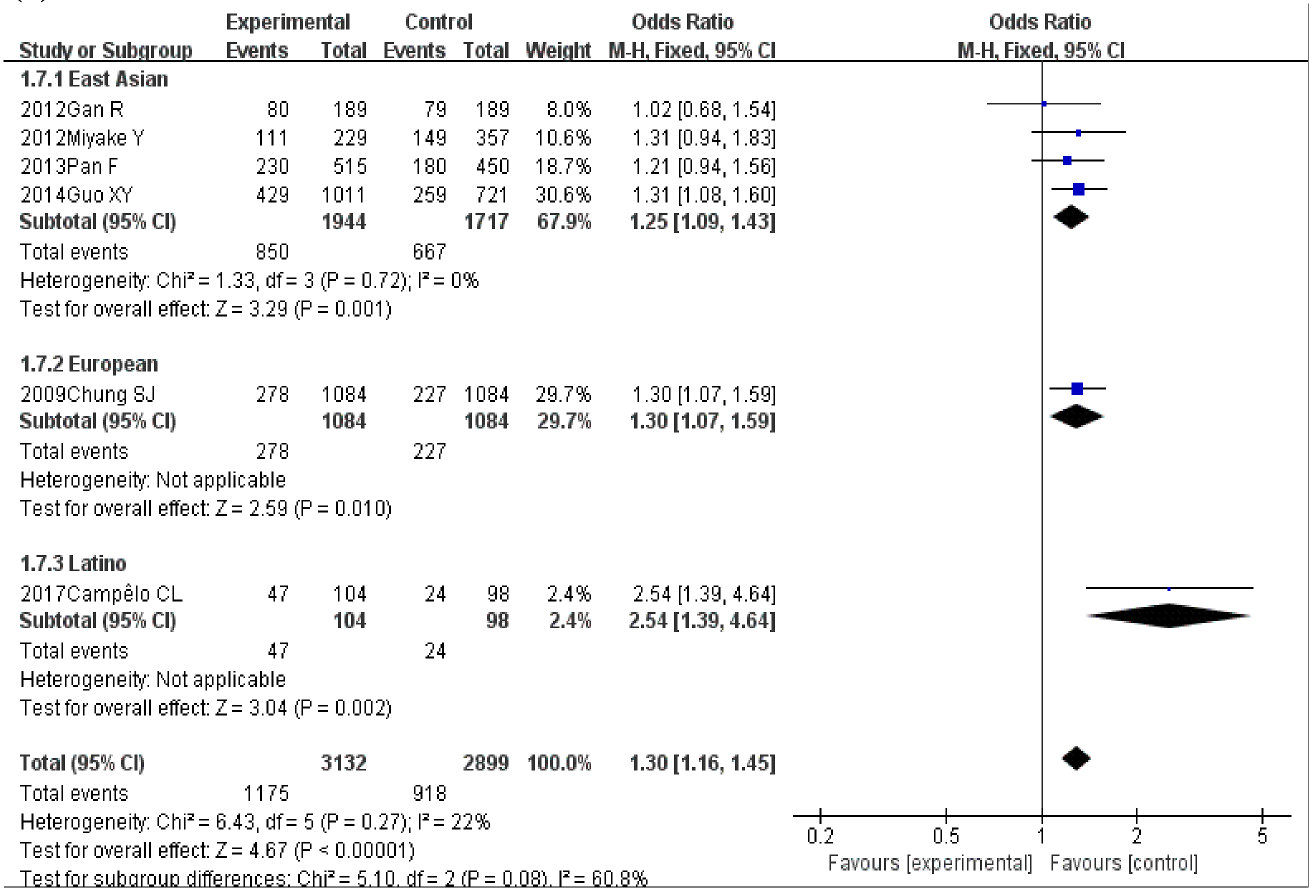

(G)

Study or Subgroup	Experimental Events	Experimental Total	Control Events	Control Total	Weight	Odds Ratio M-H, Random, 95% CI
**1.8.1 East Asian**						
2006Mizuta I	402	863	297	933	33.1%	1.87 [1.54, 2.26]
**Subtotal (95% CI)**		**863**		**933**	**33.1%**	**1.87 [1.54, 2.26]**
Total events	402		297			
Heterogeneity: Not applicable						
Test for overall effect: Z = 6.38 (P < 0.00001)						
**1.8.2 European**						
2008Westerlund M	54	288	38	300	14.0%	1.59 [1.01, 2.50]
2010Mata IF	140	684	102	671	24.6%	1.44 [1.08, 1.90]
2012Trotta L	182	904	148	891	28.3%	1.27 [1.00, 1.61]
**Subtotal (95% CI)**		**1876**		**1862**	**66.9%**	**1.37 [1.16, 1.62]**
Total events	376		288			
Heterogeneity: Tau² = 0.00; Chi² = 0.95, df = 2 (P = 0.62); I² = 0%						
Test for overall effect: Z = 3.63 (P = 0.0003)						
**Total (95% CI)**		**2739**		**2795**	**100.0%**	**1.53 [1.25, 1.87]**
Total events	778		585			
Heterogeneity: Tau² = 0.02; Chi² = 6.63, df = 3 (P = 0.08); I² = 55%						
Test for overall effect: Z = 4.16 (P < 0.0001)						
Test for subgroup differences: Chi² = 5.68, df = 1 (P = 0.02), I² = 82.4%						

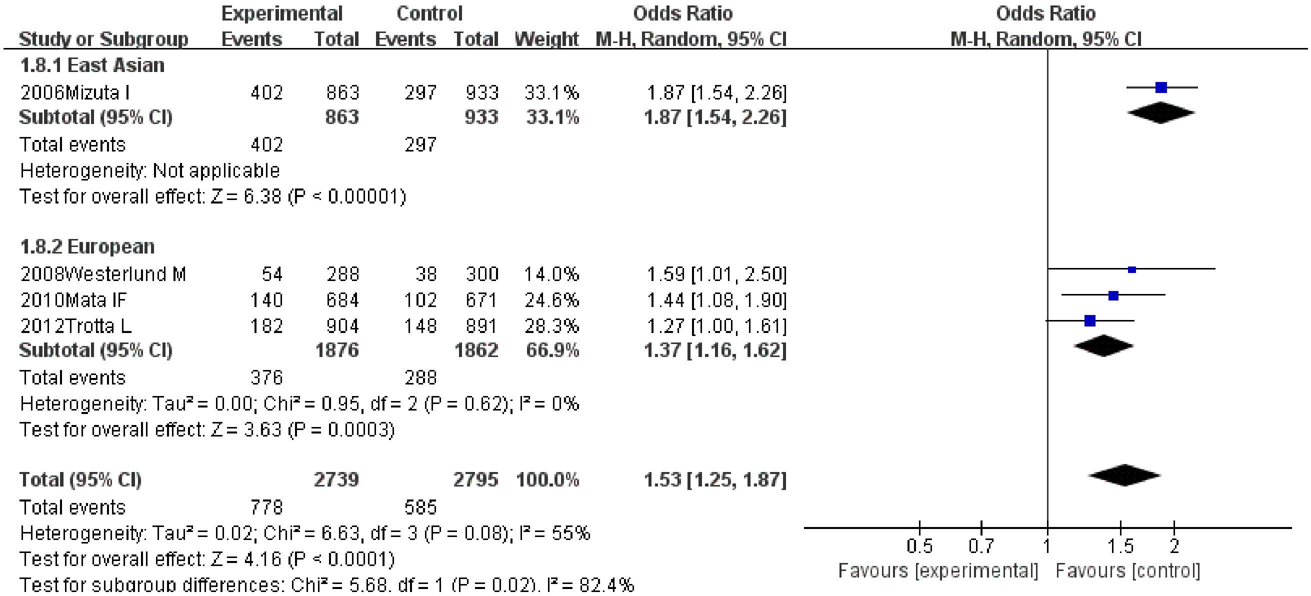

(H)

Study or Subgroup	Experimental Events	Experimental Total	Control Events	Control Total	Weight	Odds Ratio M-H, Fixed, 95% CI
**1.9.1 East Asian**						
2012Hu Y	49	110	32	136	2.4%	2.61 [1.51, 4.51]
2013Liu J	268	707	190	694	17.7%	1.62 [1.29, 2.03]
2013Wu-Chou YH	229	633	129	444	14.4%	1.38 [1.07, 1.80]
2015Chen YP	464	1269	225	840	25.5%	1.58 [1.30, 1.91]
2015Guo JF	341	1021	262	1032	25.7%	1.47 [1.22, 1.78]
2015Wu GP	49	120	43	100	4.1%	0.91 [0.53, 1.57]
**Subtotal (95% CI)**		**3860**		**3246**	**89.7%**	**1.52 [1.37, 1.68]**
Total events	1400		881			
Heterogeneity: Chi² = 8.22, df = 5 (P = 0.14); I² = 39%						
Test for overall effect: Z = 8.06 (P < 0.00001)						
**1.9.2 European**						
2007Ross OA	1	185	2	183	0.3%	0.49 [0.04, 5.47]
2007Winkler S	15	1088	4	673	0.7%	2.34 [0.77, 7.07]
2011Elbaz A(1)	6	919	4	706	0.7%	1.15 [0.32, 4.10]
2011Elbaz A(2)	5	479	1	361	0.2%	3.80 [0.44, 32.65]
2011Elbaz A(3)	2	162	0	180	0.1%	5.62 [0.27, 118.00]
2011Elbaz A(4)	15	1088	4	673	0.7%	2.34 [0.77, 7.07]
2011Elbaz A(5)	5	592	2	519	0.3%	2.20 [0.43, 11.40]
2011Elbaz A(6)	2	347	3	339	0.4%	0.65 [0.11, 3.91]
2011Elbaz A(7)	4	561	1	143	0.2%	1.02 [0.11, 9.19]
2011Elbaz A(8)	2	451	2	405	0.3%	0.90 [0.13, 6.40]
2011Elbaz A(9)	2	358	3	444	0.4%	0.83 [0.14, 4.97]
**Subtotal (95% CI)**		**6230**		**4626**	**4.3%**	**1.64 [1.03, 2.62]**
Total events	59		26			
Heterogeneity: Chi² = 5.51, df = 10 (P = 0.85); I² = 0%						
Test for overall effect: Z = 2.09 (P = 0.04)						
**1.9.3 Latino**						
2017Campêlo CL	11	104	8	98	1.1%	1.33 [0.51, 3.46]
**Subtotal (95% CI)**		**104**		**98**	**1.1%**	**1.33 [0.51, 3.46]**
Total events	11		8			
Heterogeneity: Not applicable						
Test for overall effect: Z = 0.59 (P = 0.56)						
**1.9.4 Mixed**						
2011Elbaz A(10)	3	377	4	362	0.6%	0.72 [0.16, 3.23]
**Subtotal (95% CI)**		**377**		**362**	**0.6%**	**0.72 [0.16, 3.23]**
Total events	3		4			
Heterogeneity: Not applicable						
Test for overall effect: Z = 0.43 (P = 0.67)						
**1.9.5 West Asian**						
2016Shahmohammadibeni N	56	520	32	520	4.2%	1.84 [1.17, 2.89]
**Subtotal (95% CI)**		**520**		**520**	**4.2%**	**1.84 [1.17, 2.89]**
Total events	56		32			
Heterogeneity: Not applicable						
Test for overall effect: Z = 2.64 (P = 0.008)						
**Total (95% CI)**		**11091**		**8852**	**100.0%**	**1.53 [1.39, 1.69]**
Total events	1529		951			
Heterogeneity: Chi² = 15.42, df = 19 (P = 0.70); I² = 0%						
Test for overall effect: Z = 8.68 (P < 0.00001)						
Test for subgroup differences: Chi² = 1.80, df = 4 (P = 0.77), I² = 0%						

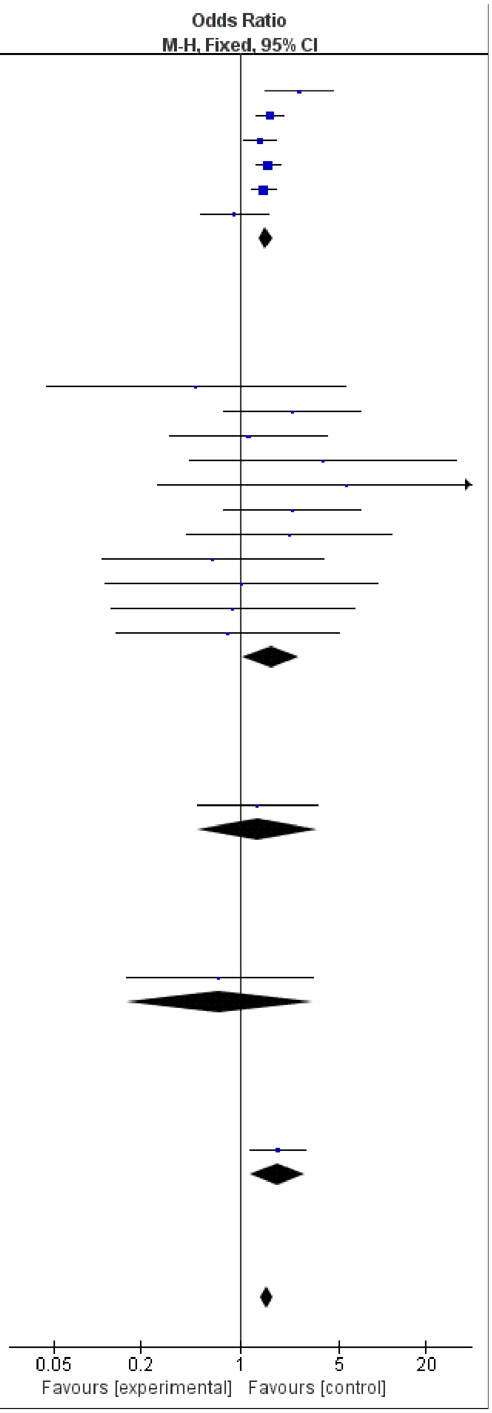

Supplementary Figure 3: Forest plots of the association between each *SNCA* variant (homozygote) included and PD risks in total and by ethnicity. (A)-(H) were individually responsive to the variants rs181489(TT), rs356165(GG), rs356186(AA), rs356219(GG), rs356220(TT), rs2736990(GG), rs2737029(GG), rs11931074(TT).
