# Supplementary material for: A Comprehensive Analysis of the Association Between SNCA Polymorphisms and the Risk of Parkinson's Disease
Source: Front Mol Neurosci. 2018 Oct 25;11:391. doi: 10.3389/fnmol.2018.00391 (PMC6209653; doi:10.3389/fnmol.2018.00391)
Supplement: Supplementary file 6 [file Image_4.PDF]

Supplementary Material

A comprehensive analysis of the association between *SNCA* polymorphisms with the risk of Parkinson's disease

Yuan Zhang[1] †, Li Shu[1] †, Qiying Sun[2,3,4], Hongxu Pan[1], Jifeng Guo[1,3,4,6,7,8], Beisha Tang[1, 2,3,4,5,6,7,8*]

† These authors have contributed equally to this work and are co-first authors.

* **Correspondence: Beisha Tang** bstang7398@163.com

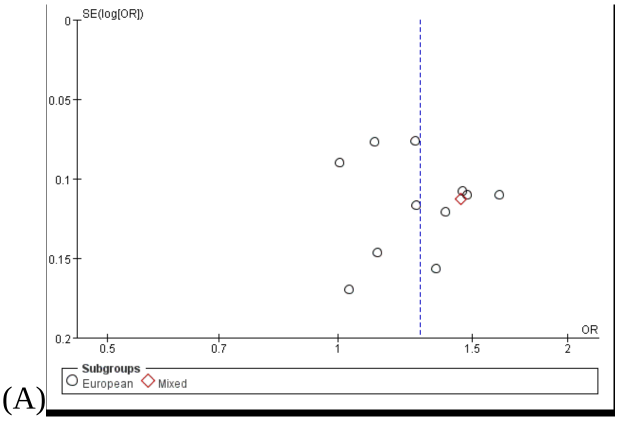

(A)

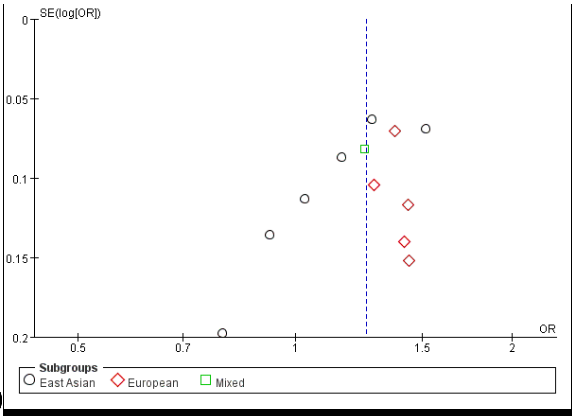

(B)

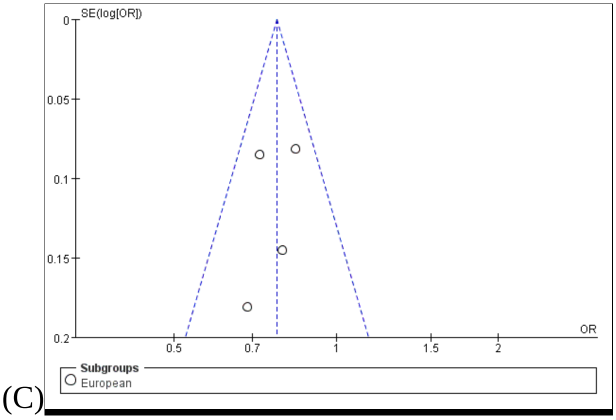

(C)

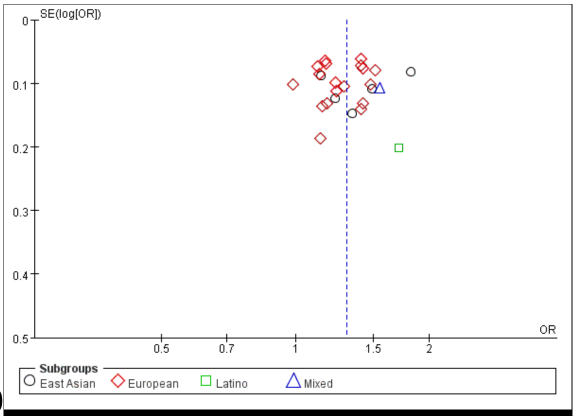

(D)

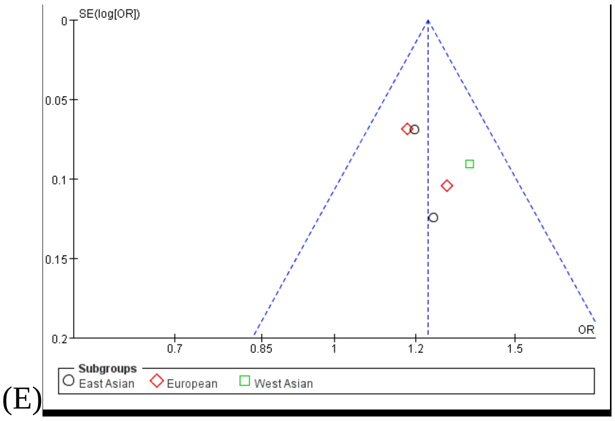
SE(log[OR])
0
0.05
0.1
0.15
0.2
0.7
0.85
1
1.2
1.5
OR
Subgroups
East Asian
European
West Asian

(E)

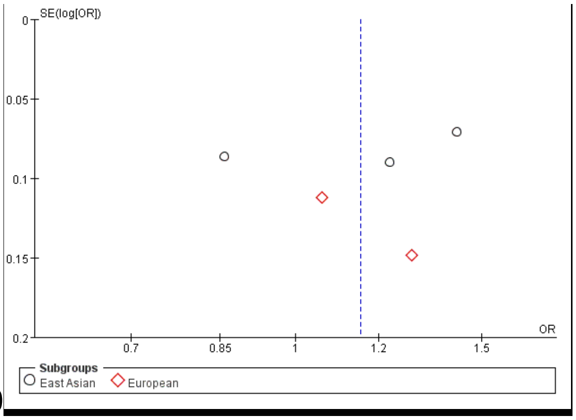
SE(log[OR])
0
0.05
0.1
0.15
0.2
0.7
0.85
1
1.2
1.5
OR
Subgroups
East Asian
European

(F)

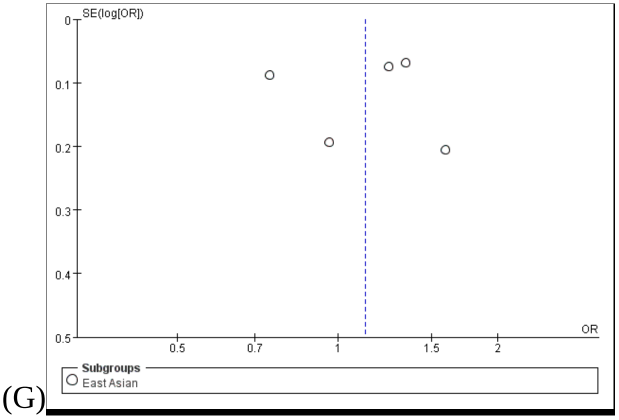
SE(log[OR])
0
0.1
0.2
0.3
0.4
0.5
0.5
0.7
1
1.5
2
OR
Subgroups
East Asian

(G)

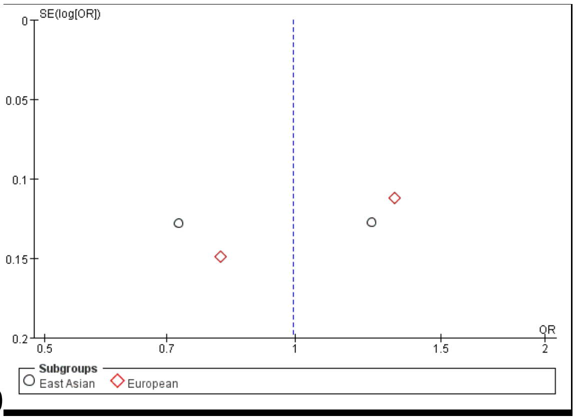
SE(log[OR])
0
0.05
0.1
0.15
0.2
0.5
0.7
1
1.5
2
OR
Subgroups
East Asian
European

(H)

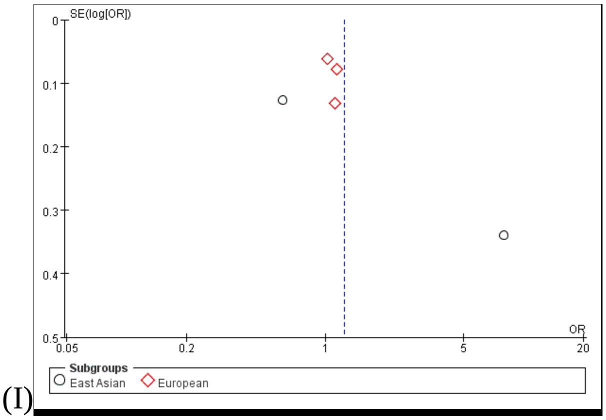
SE(log[OR])
0
0.1
0.2
0.3
0.4
0.5
0.05
0.2
1
5
20
OR
Subgroups
East Asian
European

(I)

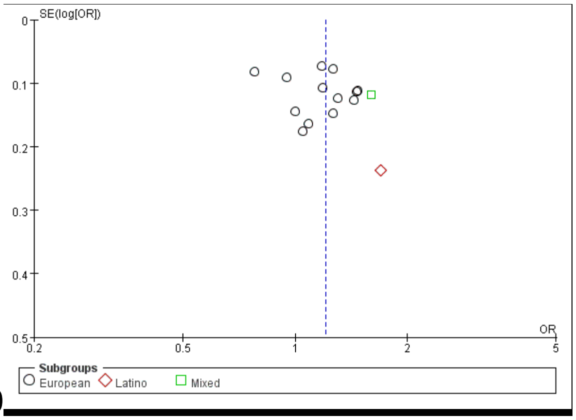
SE(log[OR])
0
0.1
0.2
0.3
0.4
0.5
0.2
0.5
1
2
5
OR
Subgroups
European
Latino
Mixed

(J)

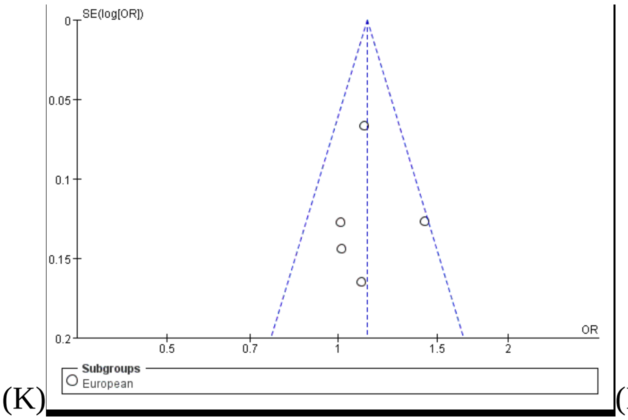
SE(log[OR])
0
0.05
0.1
0.15
0.2
0.5
0.7
1
1.5
2
OR
Subgroups
European

(K)

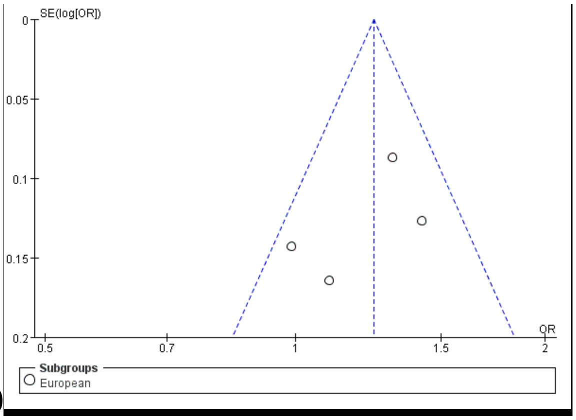
SE(log[OR])
0
0.05
0.1
0.15
0.2
0.5
0.7
1
1.5
2
OR
Subgroups
European

(L)

(M)
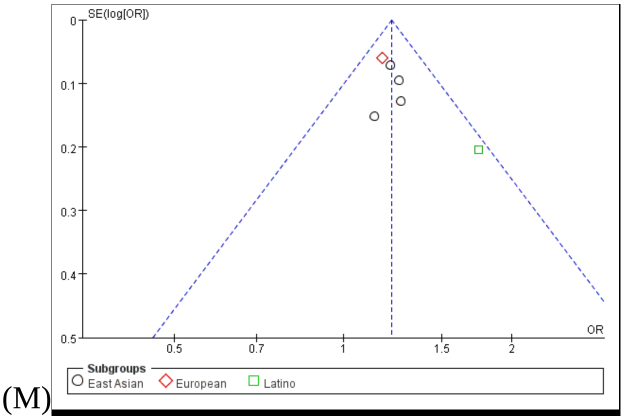

(N)
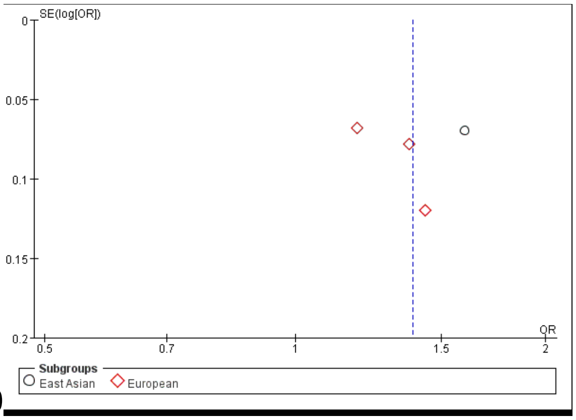

(O)
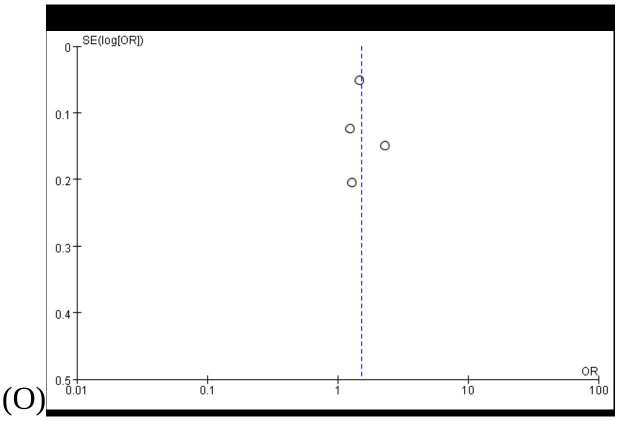

(P)
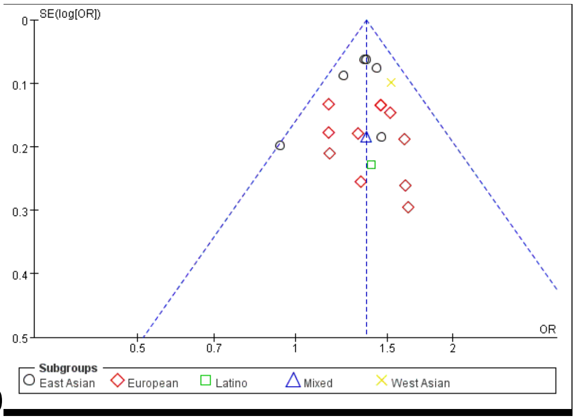

Supplementary Figure 4: Funnel plots of the association between each *SNCA* variant (allele) included and PD risks in total and by ethnicity. (A)-(P) were individually responsive to the variants rs181489(T), rs356165(G), rs356186(A), rs356219(G), rs356220(T), rs356221(A), rs894278(G), rs2301134(A), rs2301135(G), rs2583988(T), rs2619363(T), rs2619364(G), rs2736990(G), rs2737029(G), rs7684318(C), rs11931074(T).
