# Supplementary material for: A Comprehensive Analysis of the Association Between SNCA Polymorphisms and the Risk of Parkinson's Disease
Source: Front Mol Neurosci. 2018 Oct 25;11:391. doi: 10.3389/fnmol.2018.00391 (PMC6209653; doi:10.3389/fnmol.2018.00391)
Supplement: Supplementary file 7 [file Image_5.PDF]

Supplementary Material

A comprehensive analysis of the association between *SNCA* polymorphisms with the risk of Parkinson's disease

Yuan Zhang[1 †], Li Shu[1 †], Qiying Sun[2,3,4], Hongxu Pan[1], Jifeng Guo[1,3,4,6,7,8], Beisha Tang[1, 2,3,4,5,6,7,8*]

[†] These authors have contributed equally to this work and are co-first authors.

[*] **Correspondence: Beisha Tang bstang7398@163.com**

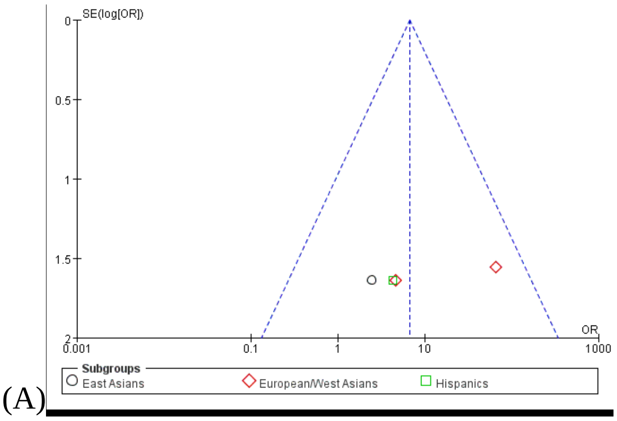

(A)

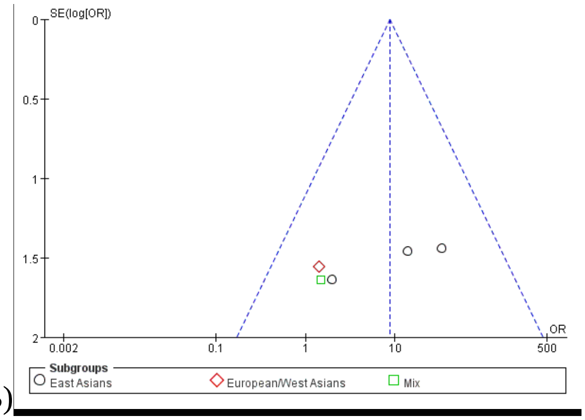

(B)

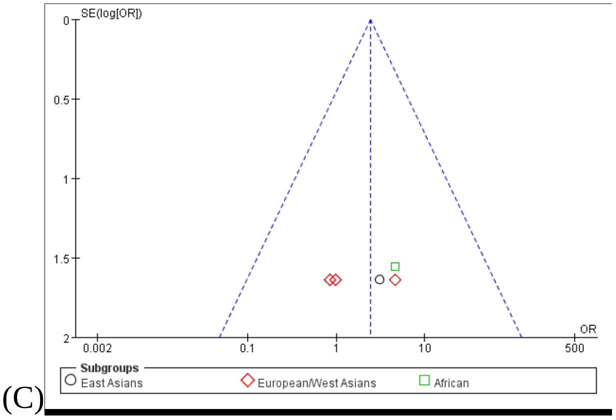

(C)

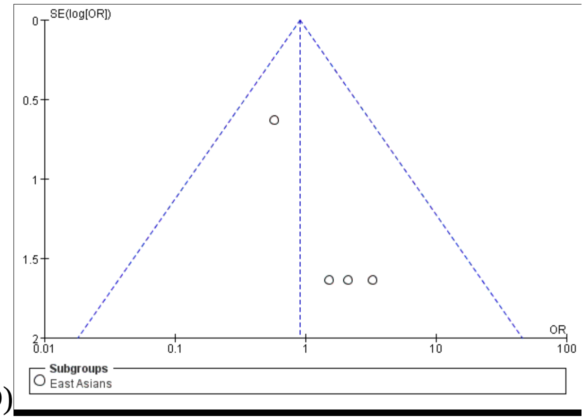

(D)

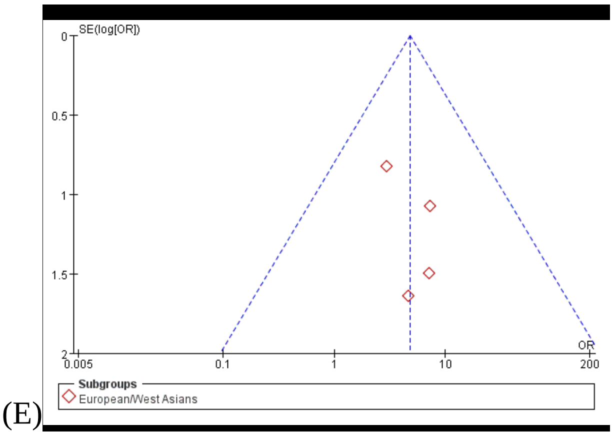
SE(log[OR])
0
0.5
1
1.5
2
0.005
0.1
1
10
200
OR
Subgroups
European/West Asians

(E)

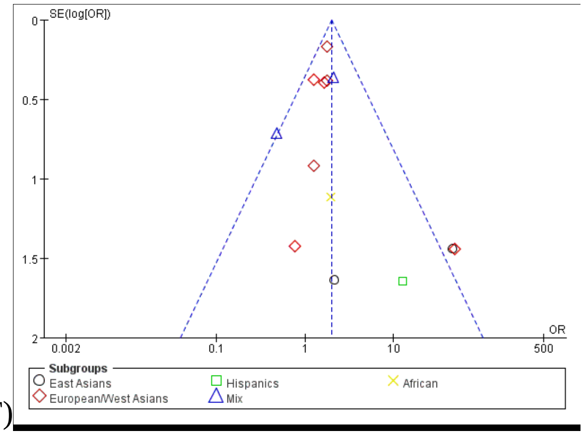
SE(log[OR])
0
0.5
1
1.5
2
0.002
0.1
1
10
500
OR
Subgroups
East Asians
European/West Asians
Hispanics
Mix
African

(F)

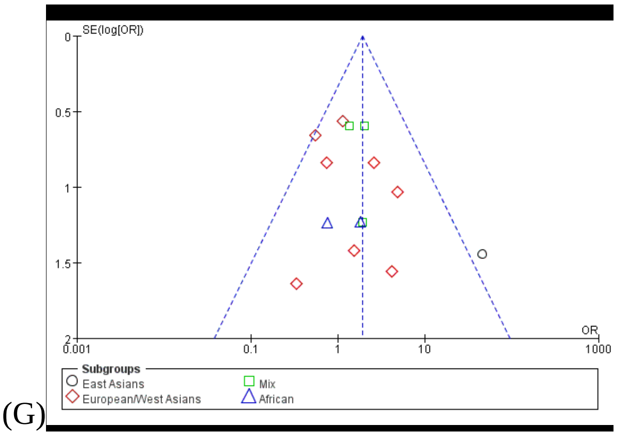
SE(log[OR])
0
0.5
1
1.5
2
0.001
0.1
1
10
1000
OR
Subgroups
East Asians
European/West Asians
Mix
African

(G)

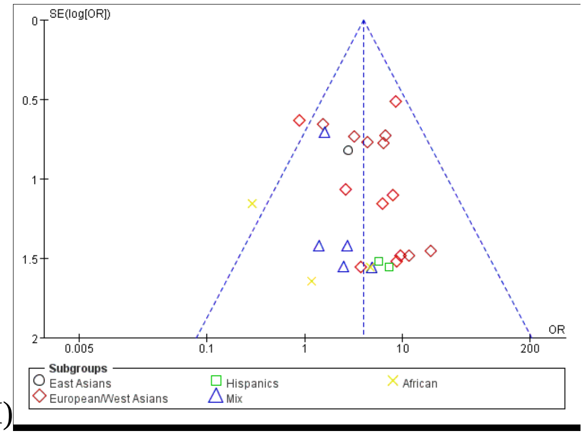
SE(log[OR])
0
0.5
1
1.5
2
0.005
0.1
1
10
200
OR
Subgroups
East Asians
European/West Asians
Hispanics
Mix
African

(H)

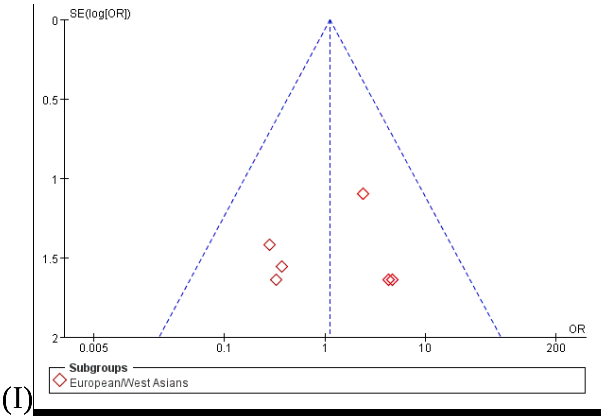
SE(log[OR])
0
0.5
1
1.5
2
0.005
0.1
1
10
200
OR
Subgroups
European/West Asians

(I)

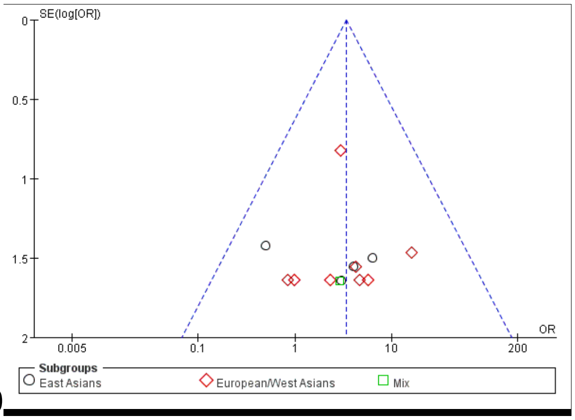
SE(log[OR])
0
0.5
1
1.5
2
0.005
0.1
1
10
200
OR
Subgroups
East Asians
European/West Asians
Mix

(J)

(K)
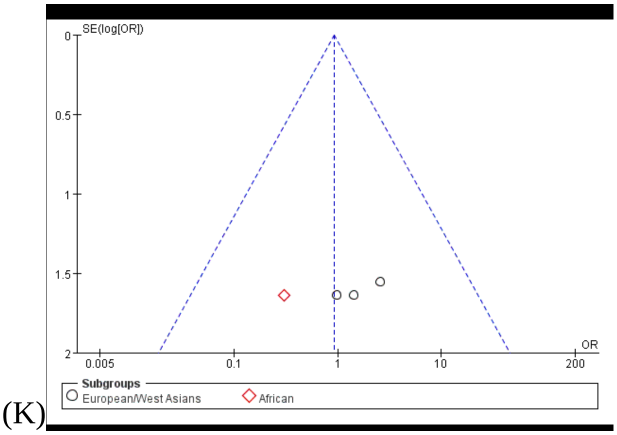

(L)
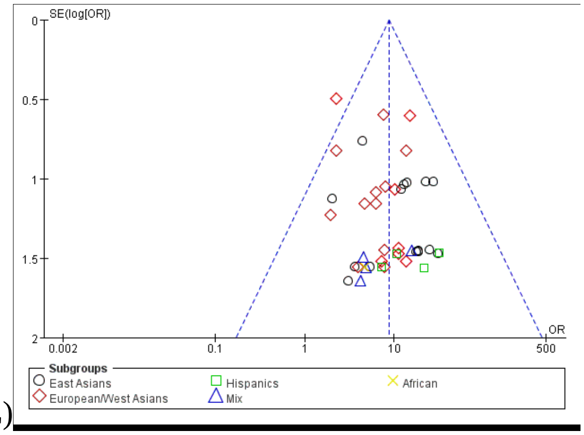

(M)
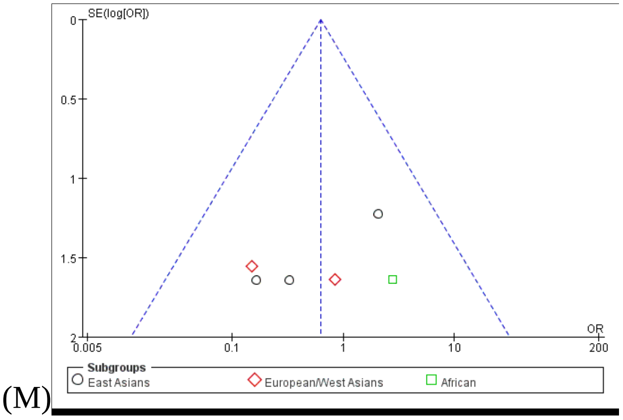

(N)
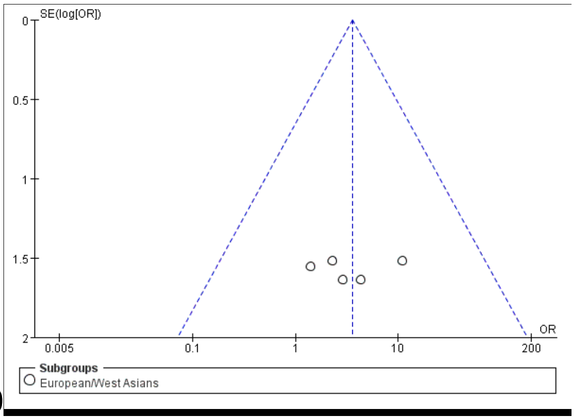

(O)
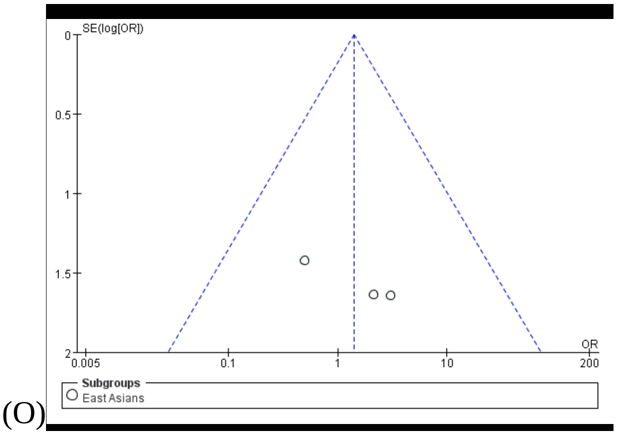

(P)
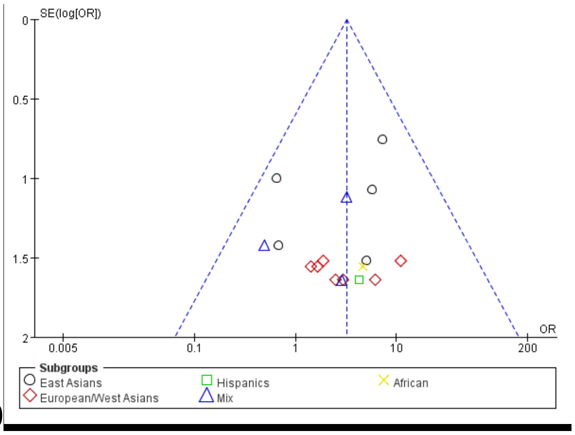

Supplementary Figure 5: Funnel plots of the association between each *SNCA* variant (heterozygote) included and PD risks in total and by ethnicity. (A)-(H) were individually responsive to the variants rs181489(TT+TC), rs356165(GG+GA), rs356186(AA+AG), rs356219(GG+GA), rs356220(TT+TC), rs2736990(GG+GA), rs2737029(GG+GA), rs11931074(TT+TG).
