# Supplementary material for: A Comprehensive Analysis of the Association Between SNCA Polymorphisms and the Risk of Parkinson's Disease
Source: Front Mol Neurosci. 2018 Oct 25;11:391. doi: 10.3389/fnmol.2018.00391 (PMC6209653; doi:10.3389/fnmol.2018.00391)
Supplement: Supplementary file 8 [file Image_6.PDF]

Supplementary Material

A comprehensive analysis of the association between *SNCA* polymorphisms with the risk of Parkinson's disease

Yuan Zhang[1 †], Li Shu[1 †], Qiying Sun[2,3,4], Hongxu Pan[1], Jifeng Guo[1,3,4,6,7,8], Beisha Tang[1, 2,3,4,5,6,7,8*]

[†] These authors have contributed equally to this work and are co-first authors.

[*] **Correspondence: Beisha Tang bstang7398@163.com**

(A)
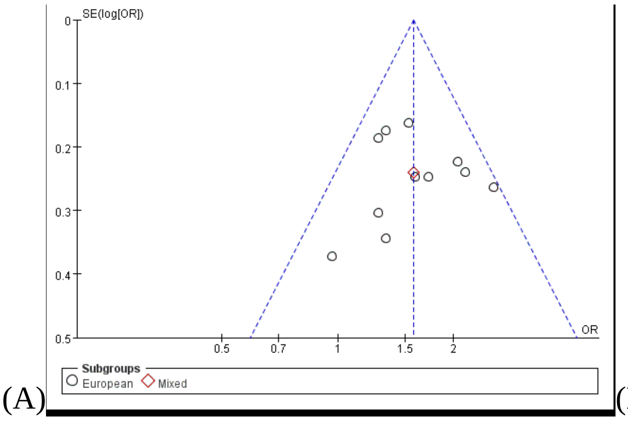

(B)
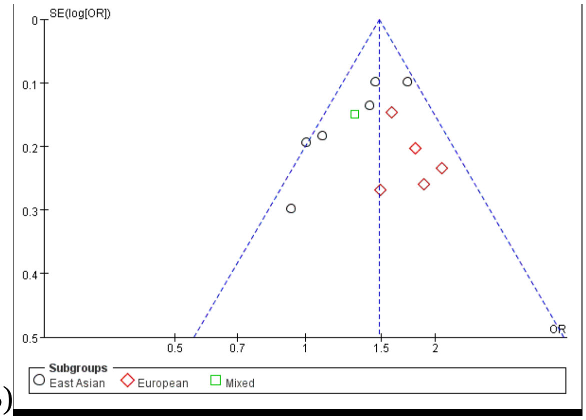

(C)
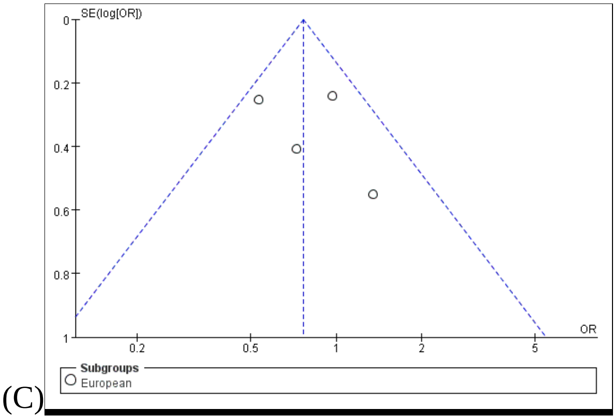

(D)
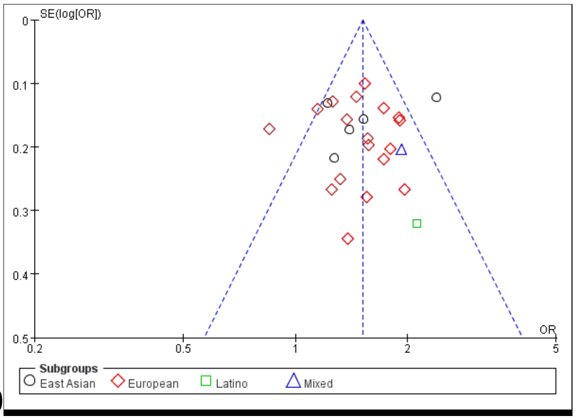

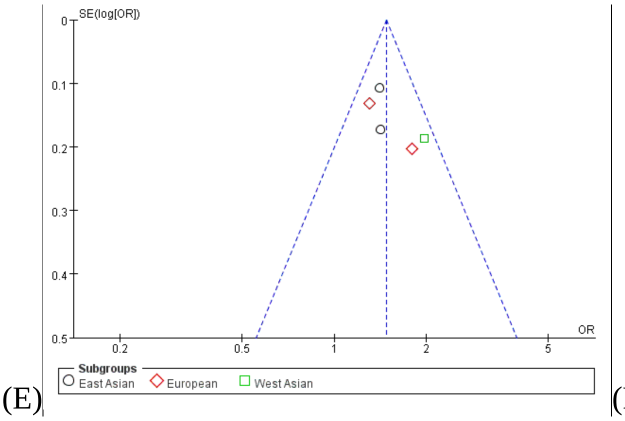

(E)

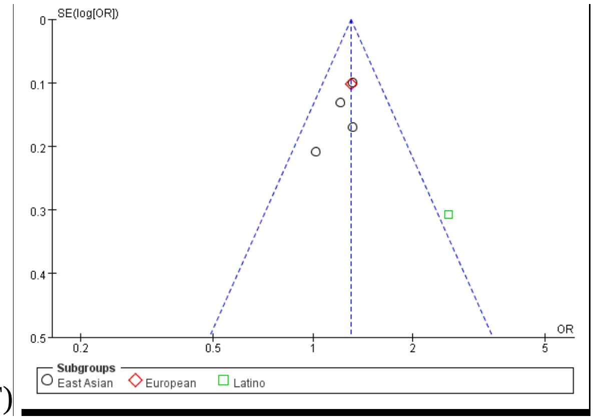

(F)

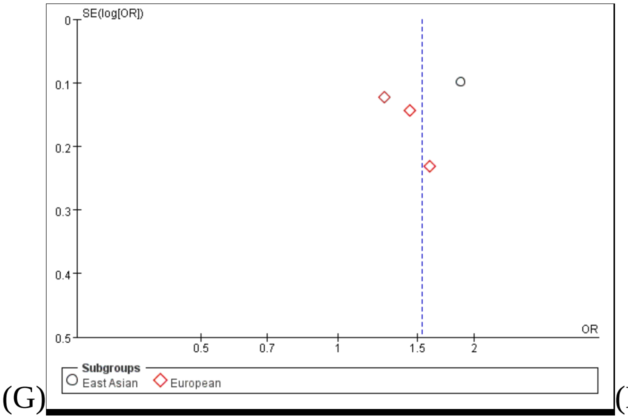

(G)

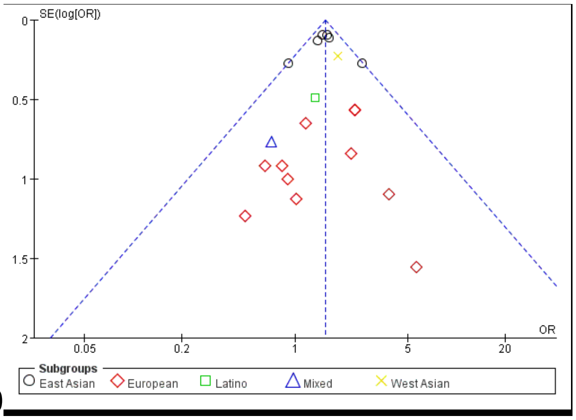

(H)

Supplementary Figure 6: Funnel plots of the association between each *SNCA* variant (homozygote) included and PD risks in total and by ethnicity. (A)-(H) were individually responsive to the variants rs181489(TT), rs356165(GG), rs356186(AA), rs356219(GG), rs356220(TT), rs2736990(GG), rs2737029(GG), rs11931074(TT).
